# Organization of residential space, site function variability, and seasonality of activities among MIS 5 Iberian Neandertals

**DOI:** 10.1038/s41598-022-24430-z

**Published:** 2022-11-23

**Authors:** Marianne Deschamps, Ignacio Martín-Lerma, Gonzalo Linares-Matás, João Zilhão

**Affiliations:** 1 UNIARQ, Centro de Arqueologia da Universidade de Lisboa, Alameda da Universidade, 1600-214 Lisbon, Portugal; 2 UMR 5608-TRACES, Maison de la recherche, 5 allées Antonio Machado, 31058 Toulouse Cedex 9, France; 3 Área de Prehistoria, Facultad de Letras, Universidad de Murcia, Calle Santo Cristo, 1, 30001 Murcia, Spain; 4 St. Hugh’s College, University of Oxford, St. Margaret’s Road, Oxford, OX2 6LE UK; 5 Present Address: Emmanuel College, University of Cambridge, St Andrew’s Street, Cambridge, CB2 3AP UK; 6 ICREA, Passeig de Lluís Companys, 23, 08010 Barcelona, Spain; 7 Department of History and Archaeology, University of Barcelona, Carrer de Montalegre, 6-8, 08001 Barcelona, Spain

**Keywords:** Archaeology, Anthropology

## Abstract

Whether ethnoarcheological models of hunter-gatherer mobility, landscape use, and structuration of the inhabited space are relevant to the archeology of Neandertals and the Middle Paleolithic remains controversial. The thin lenses of hearth-associated stone tools and faunal remains excavated in sub-complex AS5 of Cueva Antón (Murcia, Spain) significantly advance these debates. Dated to 77.8–85.1 ka, these living floors are interstratified in river-accumulated sands and were buried shortly after abandonment by low-energy inundation events, with minimal disturbance and negligible palimpsest formation. Stone tools were made and ergonomically modified to fit tasks; their spatial distributions and use-wear reveal hearth-focused activities and a division of the inhabited space into resting and working areas. Site function varied with season of the year: units III-i/j1 and III-i/j2-3 record winter visits focused on filleting and hide processing, while woodworking predominated in unit III-b/d, which subsumes visits to the site over the course of at least one winter, one spring, and one summer. These snapshots of Neandertal behavior match expectations derived from the ethnographic and Upper Paleolithic records for the lifeways of hunter-gatherers inhabiting temperate regions with a markedly seasonal climate.

## Introduction

It is becoming increasingly clear that, some 200,000 years ago, human behavior began to change in a profound manner. In Africa, by the end of Marine Isotope Stage (MIS) 5, the record of archeologically visible innovations had grown to include the increased exploitation of marine resources, the heat pretreatment of siliceous rocks, the manufacture of composite tools, the use of colorants, the wearing of shell beads, and the production of geometric signs^1,2^. These innovations were once thought to be causally linked to the emergence of anatomical modernity^3,4^, but we now know that similar developments—fishing and shell-fishing, stone tool hafting, use of bivalve shells as raw material for tools and body decoration, employment of large raptor talons as personal ornaments, intentional burial, and symbolic use of the underground world—were occurring at broadly the same time among Eurasian Neandertals^5–14^. Potentially underpinning this “Middle Paleolithic Revolution”^15^ and the patterns of convergence revealed by the archeological record, gene flow between the two continental reservoirs increased at this time; in Eurasia, such a process is revealed by the paleogenetic evidence for the replacement of an ancestral, Denisovan-related Y chromosome lineage by the modern human-related lineage seen among Upper Pleistocene Neandertals^16^.

In southern Africa, the MIS 5 behavioral watershed has been associated with demographic growth and shifts in settlement and subsistence, leading to the emergence of territoriality and economies reliant on dense and predictable resources^17,18^. The same is likely to have occurred in at least those parts of Europe that featured a comparable climate, landscape, and resource base, namely the coastal regions of southern and western Iberia—as shown by the Portuguese site of Figueira Brava, where the range of plant and marine foods procured and the scale of their exploitation by MIS 5 Neandertals is on a par with regional Mesolithic patterns^11^. Tantalizing evidence from the German site of Neumark-Nord even suggests that Neandertals at this time had already mastered the use of fire as a tool for landscape management^19^.

Seasonality plays a key role in hunter-gatherer mobility, while the structuration of the inhabited space has been considered an important archeological proxy of “modern” behavior. Alongside issues of symbolic thinking and subsistence, whether these features of ethnographically documented hunter-gatherer lifeways can be observed among Neandertals has therefore also been the subject of much debate.

In the 1990s, a view emerged, and eventually grew into predominance among scholars, that the Neandertals’ daily life and social organization were fundamentally distinct^20,21^. An extreme but by no means heterodox example is Lewis Binford’s interpretation of the Combe-Grenal rock-shelter, in France. Based on his analysis of spatial patterning across that site’s sequence of Middle Paleolithic occupations, Binford proposed that male and female Neandertals led year-round separate lives, with males visiting the sites inhabited by females and dependent children only occasionally and for brief episodes of mating. This behavior would explain the characteristic distributions of stone tools and faunal remains repetitively found at Combe Grenal: peripheral, male areas containing mostly imported raw materials and animal remains reflecting heavy-duty butchery surrounding a central, female area (the “nest”) containing mostly local raw materials and animal remains reflecting intensive extraction of bone marrow and the processing of plant resources^22,23^.

Over the last decades, ethnoarcheological research has provided eloquent illustration of the extent to which settlement-subsistence systems are conditioned by the cyclical succession of the different seasons and attendant unevenness in the year-round availability of key resources (e.g., prey animals, edible plant parts) and raw materials (e.g., wood)^24–26^. With the advent of cementochronology, much progress has been made in the investigation of seasonality in the archeological record, and a growing number of case studies illustrate the application of the method to the Middle Paleolithic of western Europe (e.g.,^27–29^).

However, inferring spatial structuration and season of occupation from archeological data remains a thorny issue because most Paleolithic sites were used in recurrent manner. In the palimpsests thereby formed, even the stratigraphically better-resolved units of analysis subsume an indeterminate number of individual events spread over time intervals of no less indeterminate, potentially significant length. As a result, the specific spatial and seasonal signatures of individual events are erased to a considerable extent, if not completely. Where the Middle Paleolithic is concerned, these complications are compounded by the fact that our radiometric dating methods lack the precision required to constrain the documented activities to timeframes shorter than the duration—typically, no more than a couple of millennia—of the rather abrupt, stadial/interstadial climate oscillations revealed by available high-resolution records, namely the Greenland ice cores^30^.

The interpretation of seasonality signals in terms of adaptation is particularly hampered by these factors. For instance, when more than one season is represented in the palimpsest, no direct link can be established between a given season and a given set of activities (as revealed by e.g., the use wear analysis of the stone tools). Even when the analysis of the faunal remains only identifies one and the same season of occupation, the chronological data may imply that the accumulation of the remains spanned one or more stadial/interstadial cycles, raising difficult issues of equifinality that make site function analysis problematic. One might for instance be led to infer long-term stability of the settlement-subsistence system under study, irrespective of climactic oscillations; would one though be able to ascertain that the tasks carried out at the site during the identified season remained the same throughout (for instance, would one be able to exclude, e.g., cold season use for logistical hunting during stadials versus cold season use for long-term residence during interstadials)? In your ordinary cave or rock-shelter site one clearly would not, because stone tools typically demonstrate a wide range of activities and individual stratigraphic units typically represent long-term accumulations.

**Figure 1:**
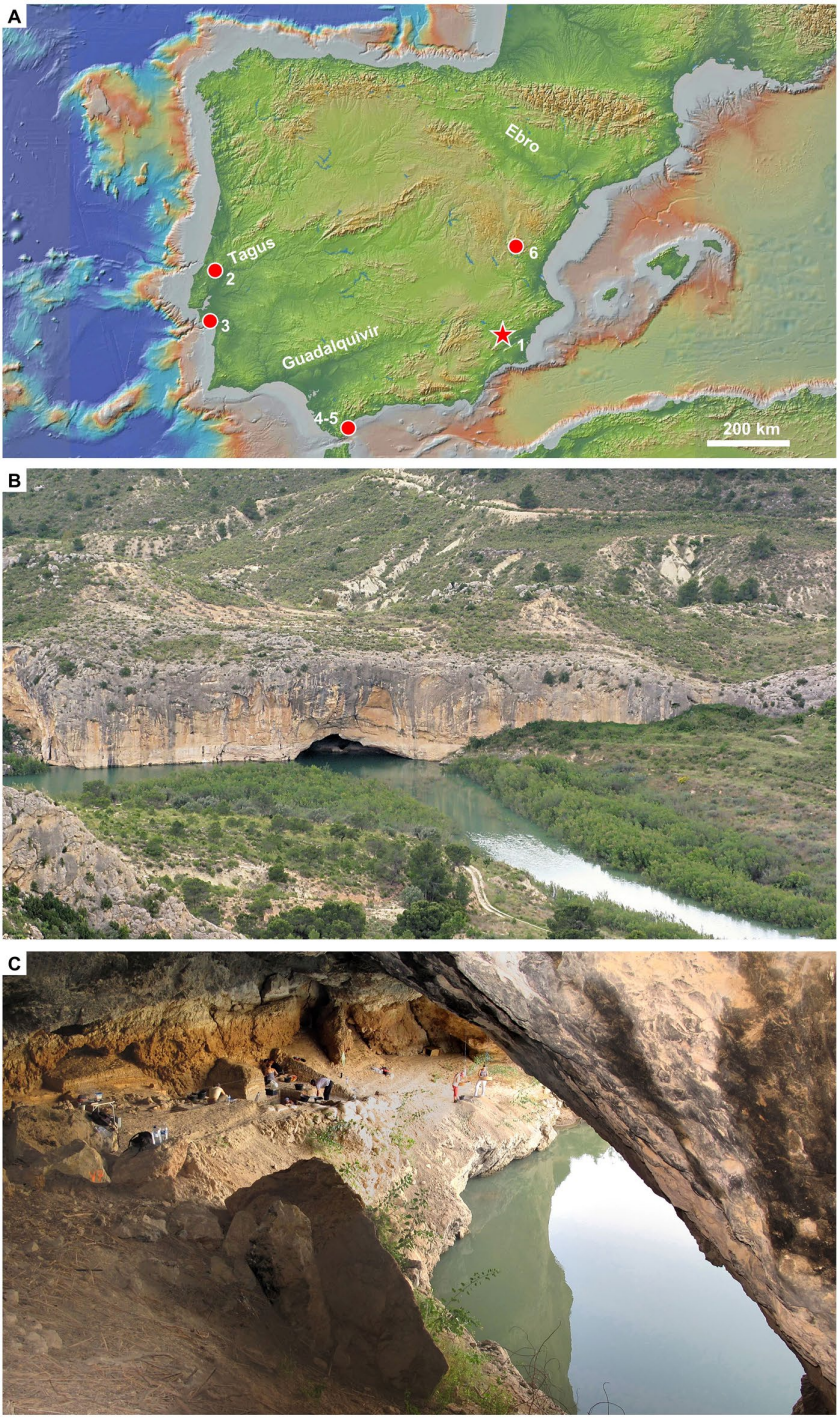
Location. (**A**) MIS 5 Iberian Middle Paleolithic sites mentioned in the text. **1.** Cueva Antón; **2.** Gruta da Oliveira; **3.** Gruta da Figueira Brava; **4.** Gorham’s Cave; **5.** Vanguard Cave; **6.** Abrigo de la Quebrada. Base map: Global Multi-Resolution Topography Synthesis (https://www.gmrt.org/GMRTMapTool/) (^70^). (**B**) Exterior view of Cueva Antón from the North (2009). (**C**) Interior view of Cueva Antón from the East (2012 field season).

To assess daily life and seasonal variability in activity patterns or mode of site usage among prehistoric hunter-gatherers, we therefore need single-event occupation records where, in addition, organic remains are well preserved and so is the use wear evidence borne by stone tools—the classical example being the Magdalenian site of Pincevent (France)^31^. Due to taphonomic biases, such contexts are rare and become increasingly so as we go back in time. The cave/rock-shelter of Cueva Antón (Mula, Murcia, Spain; 38° 3′ 51.84″ N; 1° 29′ 47.20″ W; Fig. 1) is, in this regard, exceptional: a Middle Paleolithic site where such conditions are satisfied.

Cueva Antón opens on the right bank of the Mula river, between 351 and 359 m asl (above modern sea level), *c.* 3–4 m above the stream bed and at the gates of the El Corcovado gorge, which construction of the La Cierva dam submerged in the 1940s (Supplementary Fig. S1). The site was first excavated in 1991, in the context of environmental impact assessment work carried out in relation to a projected raising of the dam, and more extensively in 2007–2012. This work revealed a sedimentary succession whose basal reaches harbor a late MIS 5, high-resolution archeological stratigraphy preserving a near-pristine record of brief, separate human occupation events.

A number of publications have already addressed Cueva Antón’s site formation process, chronology and stratigraphic configuration, the micromorphological analysis of the succession and of the hearth features therein preserved, the archeology and radiocarbon dating of the Late Mousterian occupation in layer I-k, and the composition of the assemblages of plant and faunal remains (taphonomy, mode of accumulation, paleoenvironmental significance, human subsistence, and seasonality)^32–39^. Here, building on that knowledge base, we use stone tool refitting coupled with lithic technology, use-wear analysis, and the study of spatial distributions to carry out a holistic assessment of the living floors preserved in sub-complex AS5, addressing site function and the spatial organization of activities in relation to the season of occupation. We focus on our key findings and their implications; additional detail and more extensive descriptions and interpretations of the living floors are provided in Supplementary Materials.

## Results

### Assemblage integrity

**Figure 2:**
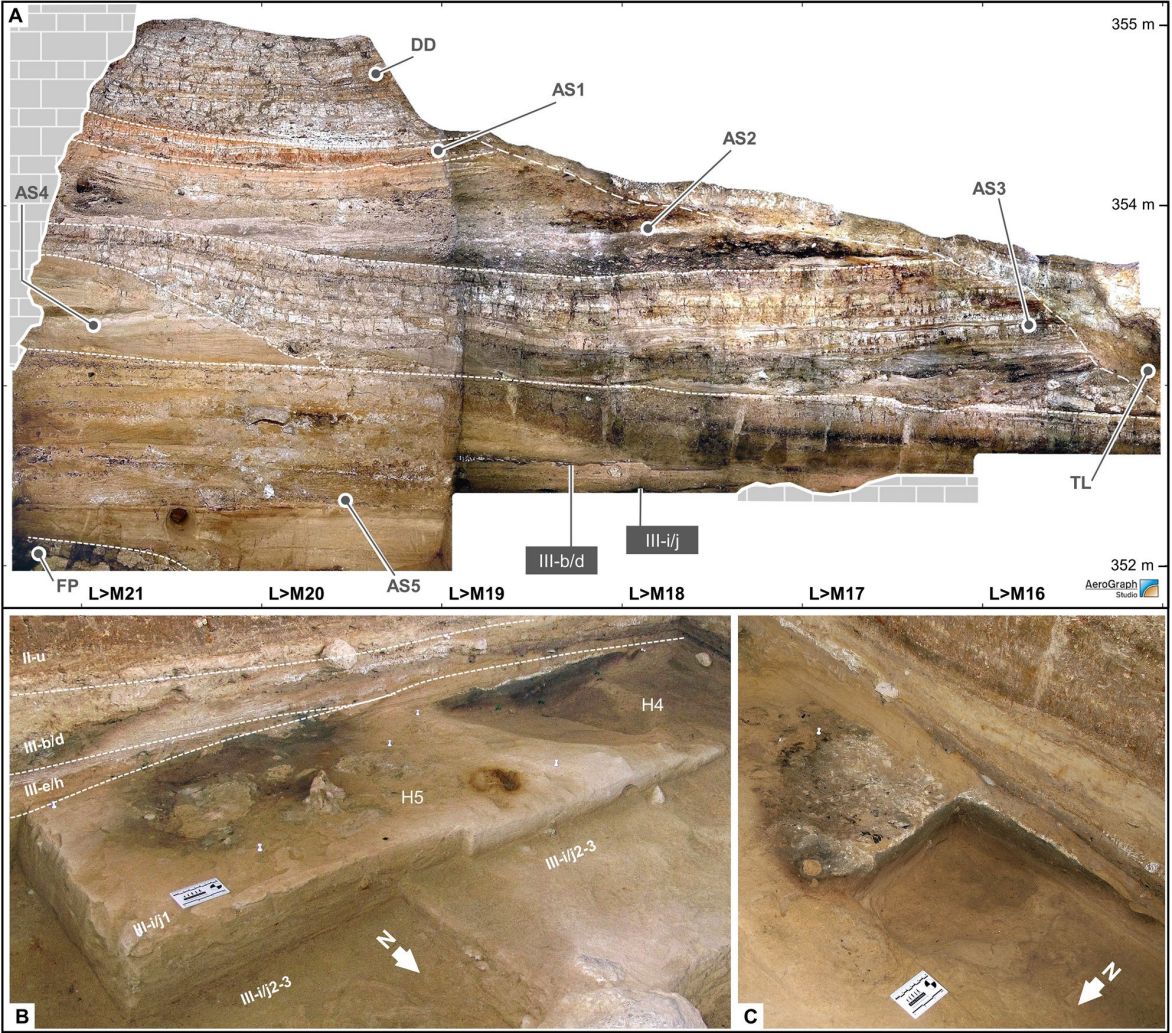
Stratigraphy and hearths. (**A**) The reference profile, with indication of the main stratigraphic subdivisions (elevations in m asl). (**B**) Oblique view of the baulk containing the H4 and H5 hearths during the excavation of layer III-i/j (2012 field season). (**C**) Detail of H4 during the initial phase of its excavation, showing the internal micro-stratigraphy typical of in situ fire features: a white ash lens denoting the occupation surface and overlying a basin-shaped, red-rimmed volume of black sediment denoting the burning of subsurface organic matter.

The bulk of the sedimentary accumulation corresponds to a 3-m-thick Upper Pleistocene fluvial terrace: complex AS (Archaeological Succession) (Fig. 2, Supplementary Text S1.1, Supplementary Figs. S2–S9). Fleeting visits represented by very small assemblages of faunal remains, wood charcoal, and stone tools are found in layer II-l of sub-complex AS2, which dates to the later part of MIS 5a, and in layer I-k of sub-complex AS1, which dates to *c.* 37 ka.

**Figure 3:**
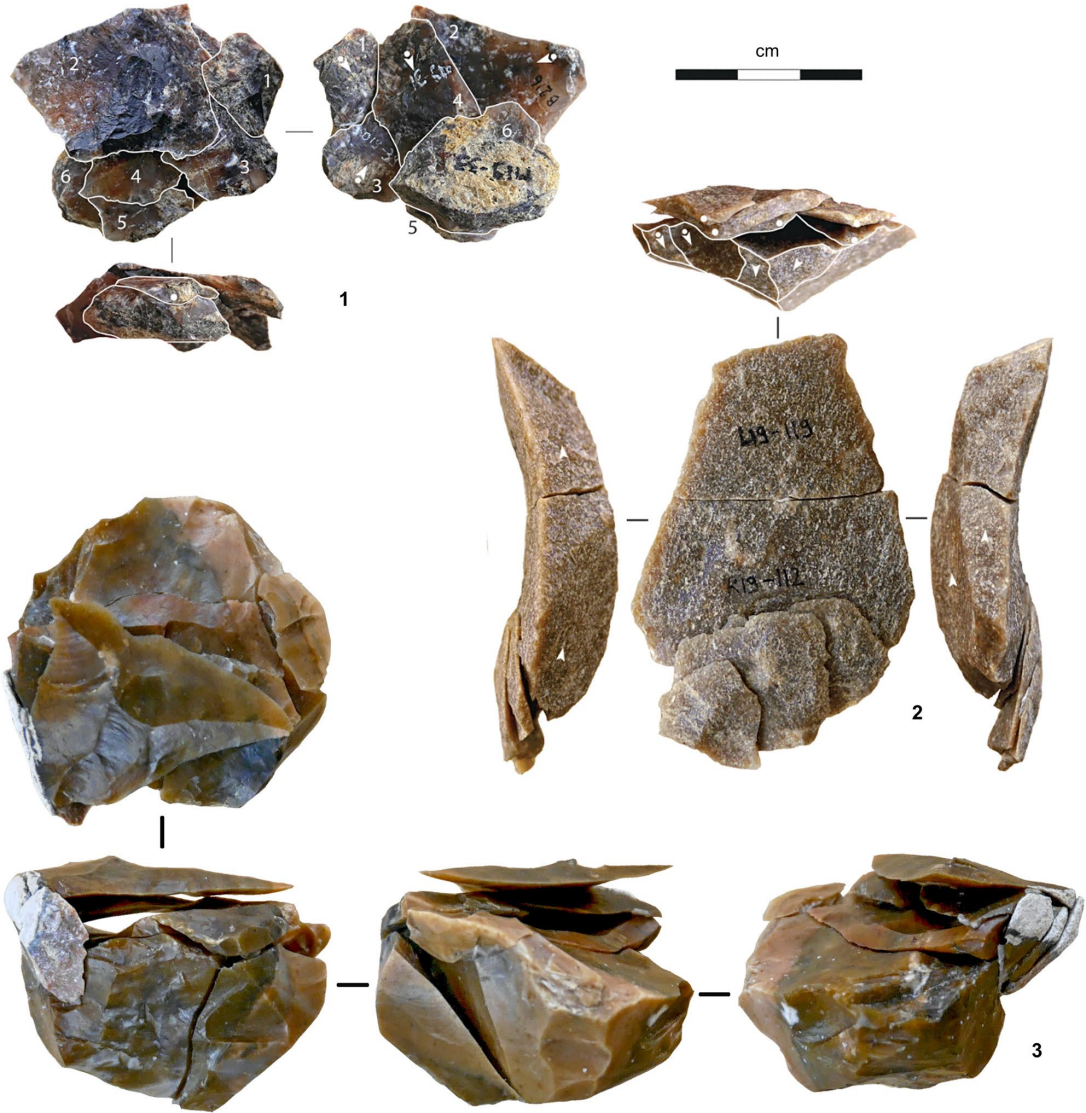
Refitting. **1.** Refit #5 (layer III-b/d; five flakes refitted on a Levallois, centripetal core; chert); **2.** Refit #59 (layer III-i/j, lens III-i/j1; seven items; conjoined broken flake exploited as Kombewa core; chert), **3.** Refit #45 (layer III-i/j, lens III-i/j2-3; nine flakes refitted on a Levallois core).

At the base, in sub-complex AS5, the archeological remains are associated with hearths and form discrete lenses interstratified in sandy, low-energy alluvial deposits that buried them quickly after abandonment and with minimal disturbance, as corroborated by the refitting evidence (Fig. 3). OSL dating coupled with paleoenvironmental constraints place the accumulation of AS5 in the 77.8–85.1 ka (thousands of years ago) interval, at the beginning of MIS 5a and within the almost 7.5 millennia-long GI (Greenland Interstadial) 21. At that time, as revealed by the AS5 charcoal assemblage, dominated by *Pinus halepensis* and including deciduous and evergreen oaks, regional environments were like present-day^35^. Within AS5, the archeological layers are III-b/d and III-i/j, which are separated across most of the excavated area by sterile layer III-e/h. A minor erosional discontinuity that could be followed across the areas excavated in 2011–2012 allowed for the subdivision of layer III-i/j into two lenses, III-i/j1 and III-i/j2-3.

In layer III-b/d, the orientation pattern and the length of refit connections imply a significant amount of syn-depositional displacement of finds along the E-W dip of the stratification, consistent with its thickness increasing along that axis of the deposit; an in situ, albeit superficially eroded fire feature (Hearth H1, excavated 1991) could nonetheless be recognized at the top of the slope, in grid units J-K/20-21 (Supplementary Text S2.1, Supplementary Figs. S10–S14 and S27–S28). Otherwise, the refitting evidence reveals complete stratigraphic separation, with no post-depositional mixing, between (a) the lithic assemblages of III-i/j1 and III-b/d (even in those parts of the trench where intermediate layer III-e/h is missing), and (b) the lithic assemblages of the III-i/j1 and III-i/j2-3 lenses.

The III-i/j1 lens subsumes two different occupations; a thin deposit of laminated sands, detectable in cross-section only and denoting the occurrence of an inundation event, clearly separates its H4 and H5 hearths (Supplementary Text S2.1, Supplementary Fig. S3). However, the finds associated with H4 can be taken to represent no more than negligible background noise because their number is very limited; therefore, the III-i/j1 lithic and faunal remains can legitimately be used as a whole to illustrate spatial structuration and activity records pertaining to the subsequent occupation event, which was organized around Hearth H2 (excavated in 1991) and Hearth H5 (excavated in 2012) (Supplementary Figs. S29–S30). The III-i/j2-3 lens consists of two contiguous clusters, III-i/j2 and III-i/j3. Refitting shows that they represent not the horizontally separated remains of consecutive occupations taking place on different parts of the same surface but separate activity areas within a single occupation event, each organized around a different hearth: H3, excavated in 2011, and H6, excavated in 2012 (Supplementary Text S2.1, Supplementary Figs. S31–S32).

### Technology and use-wear

Across the three units of analysis, chert and limestone are the raw materials used to make stone tools. Limestone dominates in all of them by weight; by number, however, chert represents only 37% of the finds in III-b/d, and 68% and 74% in III-i/j1 and III-i/j2-3, respectively. As known chert sources are at significant distance from the site, this variation bespeaks of a more intensive reduction of the immediately available raw material—limestone cobbles procured in alluvial valley bottoms—during the III-b/d occupation (Supplementary Text S2.2.1, Supplementary Tables S1–S2).

**Figure 4:**
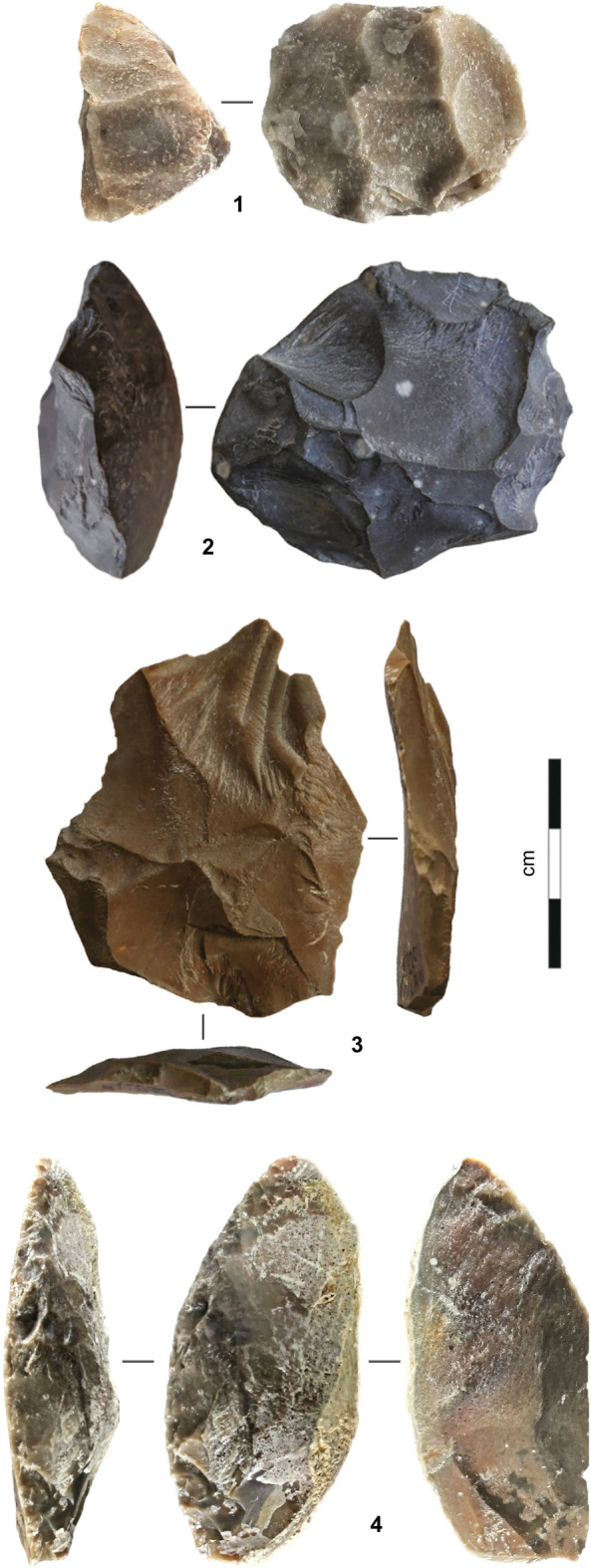
Stone tools (layer III-b/d). **1.** Chert core; **2.** Limestone core; **3.** Levallois flake (limestone); **4.** Sidescraper (chert).

**Figure 5:**
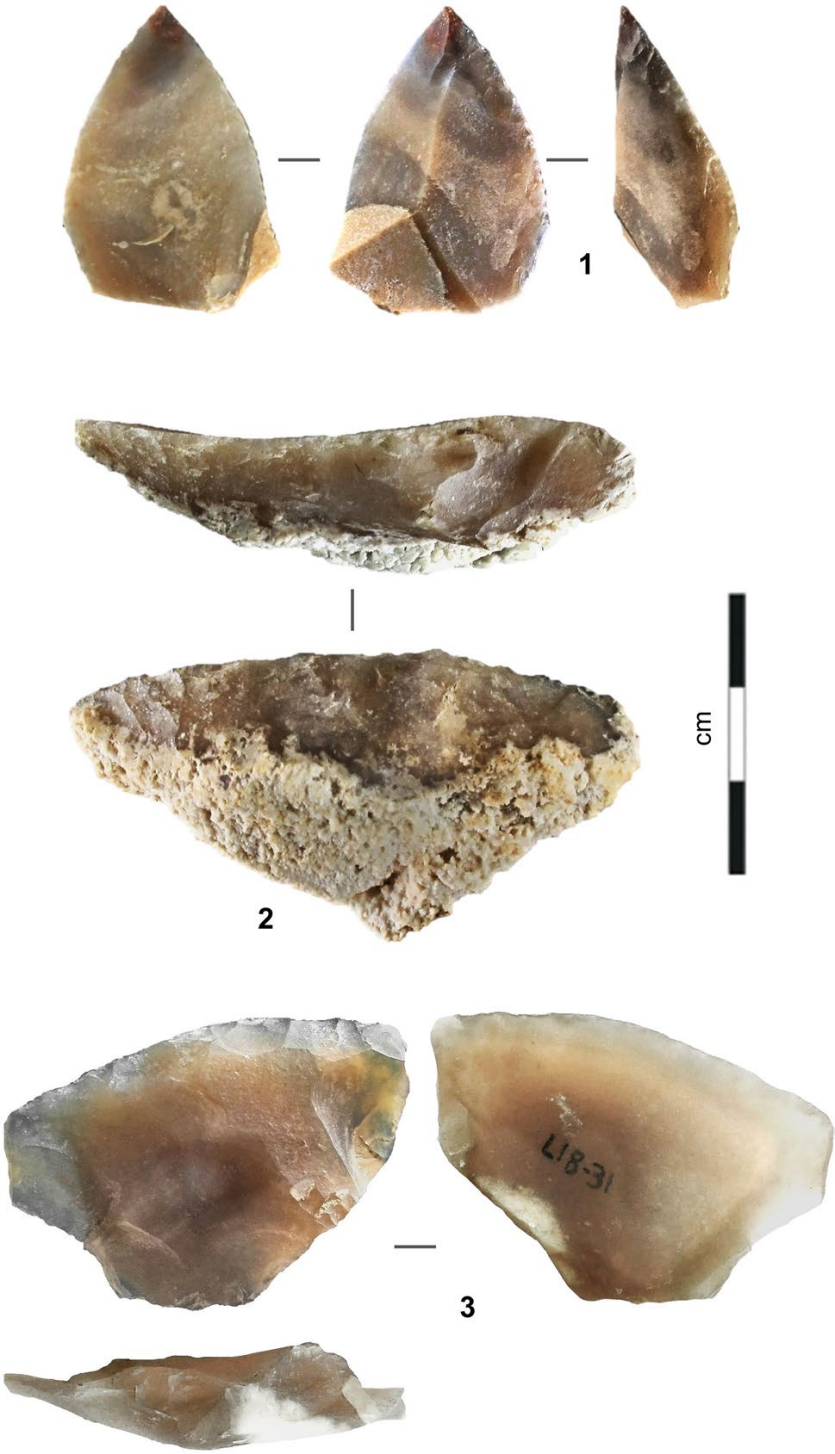
Stone tools (layer III-i/j, lens III-i/j1). **1** Bilaterally pointed tool (chert); **2–3.** Sidescrapers (chert).

**Figure 6:**
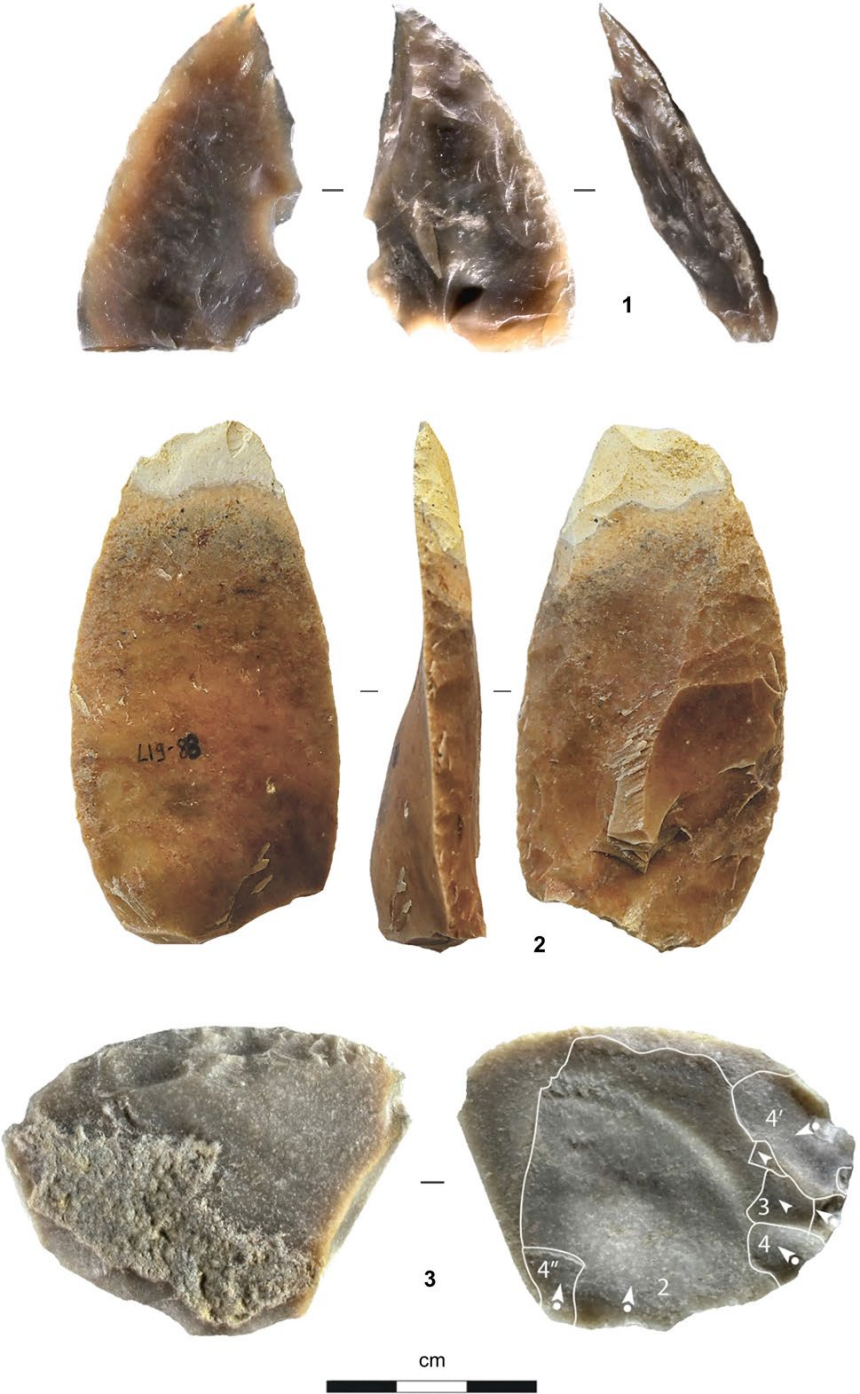
Stone tools (layer III-i/j, lens III-i/j2-3). **1–2.** Sidescrapers (chert); **3.** Kombewa core on broken sidescraper (chert).

From a technological standpoint, Levallois debitage was the primary débitage method. The variation seen across the sequence is minor and mostly explained by raw material economy. Examples of such variation are the exploitation of the ventral surfaces of worn-out tools for the extraction of small blanks, only seen in III-i/j2-3, or the use of limestone to make Levallois flakes, only common in III-b/d (Supplementary Text S2.2.2, Supplementary Figs. S15–S16). The retouched toolkit is mostly made up of sidescrapers, followed by partially retouched flakes; notches and denticulates are rare, and retouched points were found in III-i/j2-3 only (Figs. 4, 5, 6; Supplementary Text S2.2.3, Supplementary Figs. S17–S20, Supplementary Tables S3–S6).

**Figure 7:**
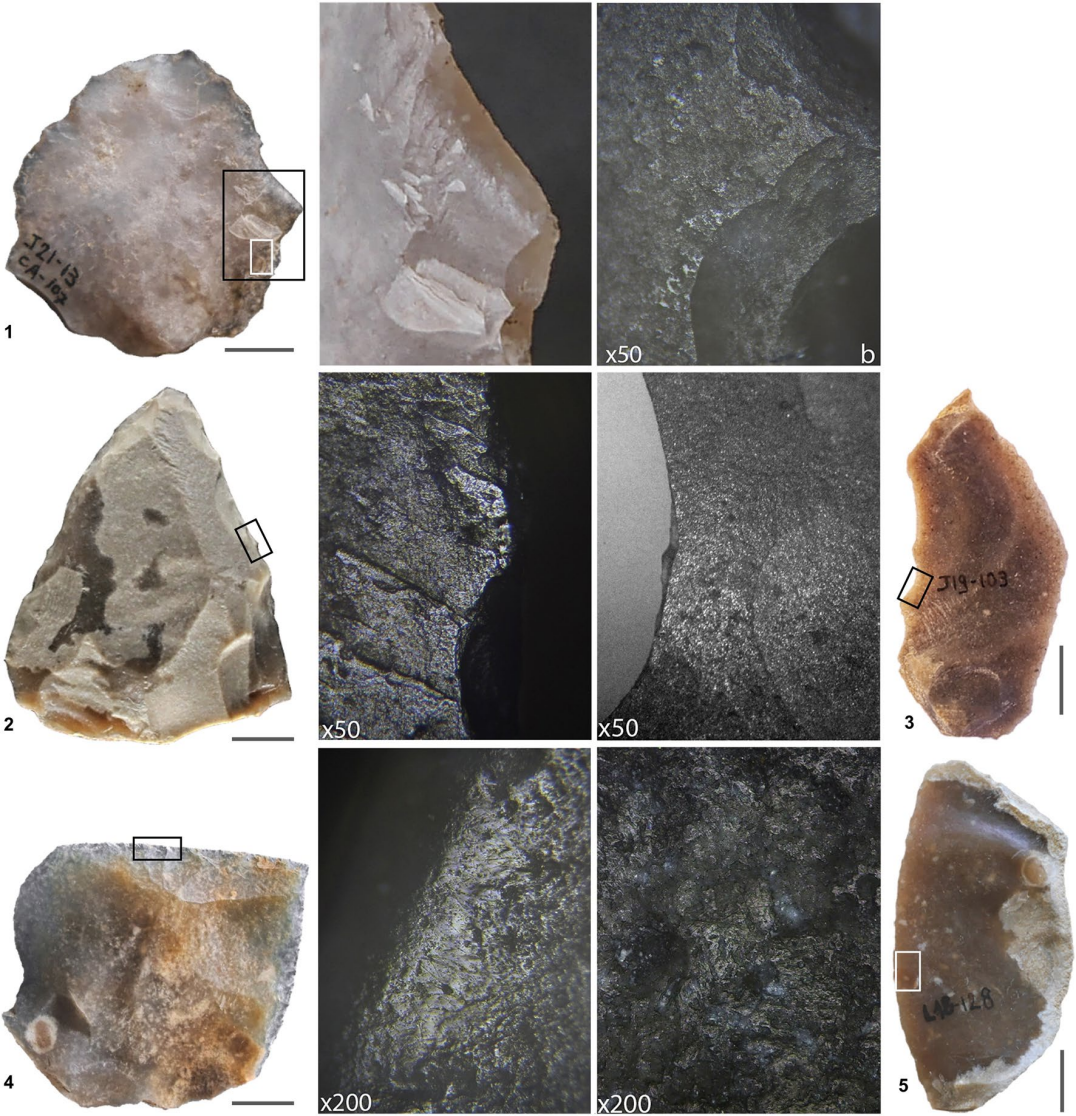
Use-wear. **1**. III-i/j1 Levallois flake (J21-13; chert) with evidence of on-bone work (small rectangle) in relation with the large flexure break; **2.** III-i/j3 sidescraper (O19-189; chert) with evidence of on-wood work; **3.** III-i/j1 elongated Kombewa blank (J19-103; chert) with evidence of meat-cutting work; **4–5.** III-i/j2 sidescrapers with evidence of hide work (left, L19-140, with abrasive; right L18-128, without). Scale bars are 1 cm.

Of the 150 retouched and unretouched blanks whose good macroscopic surface condition warranted further examination and were complete enough, a significant proportion yielded diagnostic use-wear when examined under the microscope (Fig. 7). In III-i/j1 and III-i/j2-3, animal product-processing tools predominate over wood-processing ones, the reverse happening in III-b/d. In the latter, wood-working items represent 48% and filleting items 8% of the tools with documented use-wear, while the corresponding values for III-i/j1 and III-i/j2-3 fall in the 20–22% and the 33–35% range. These differences are statistically significant (Supplementary Text S2.3.1–S2.3.2, Supplementary Figs. S21–S25, Supplementary Tables S7–S8).

The tools used to process dry hide with an abrasive substance are all sidescrapers and they constitute a distinct morphometric ensemble, clearly separated from the others based on the angle of the cutting edge: ~ 58° on average, much wider than among the tools used to process wood or meat, all of which feature < 47° angles. Whether such is also the case with the set of tools used to process fresh hides cannot be ascertained because the sample is too small (only three could be identified). The blanks used to cut meat also form a distinct cluster, as most (> 90%) remained unretouched and feature much narrower cutting-edge angles (~ 29° on average). These data reveal a clear relation between the form of the tool and the last function it was put to prior to discard (Supplementary Text S2.3.3, Supplementary Fig. S26, Supplementary Table S9).

### Spatial structuration

The site was inundated subsequent to the 2012 fieldwork season, and ever since it has been impossible to carry out the additional excavations required to expose the totality of the different living floors (Supplementary Text S1.1.1). Even though the analysis of spatial distributions is hampered by the unavailability of the parts thereof that remain buried, a number of conclusions can be drawn.

**Figure 8:**
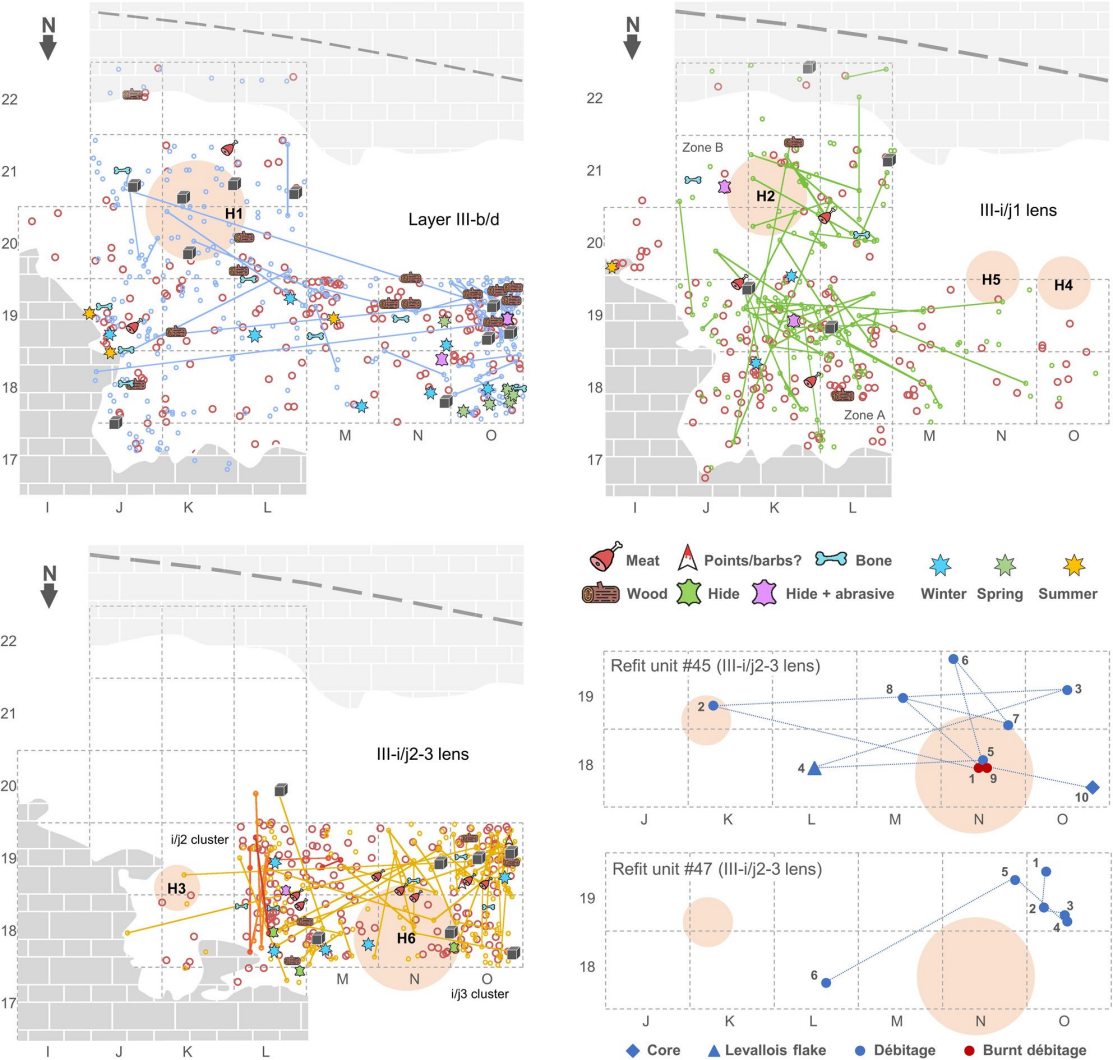
Spatial organization. Schematic plans of the living floors illustrating the distribution of faunal remains (tawny circles) and stone tools in relation to the hearths. Refitting connections are indicated, the grey cubes denote cores on block, and the markers denote the positions of the lithics that bear diagnostic use-wear or of the faunal remains that yield seasonality information. The outline of the bedrock shelf bounding the infill to the North is given as observed at the elevation of the layers’ surface. The outline of the back wall is given as observed at the top of the sedimentary infill, prior to excavation, and, approximately (as inferred from the concave configuration displayed in the J > I, L > M, and N > M stratigraphic profiles), at the layers’ surface (the long-dash line). The horizontal distribution of the items in Refits #45 (two way) and #47 (one way) are shown separately to illustrate the connection between the III-i/j2 and III-i/j3 clusters of the III-i/j2-3 occupation event.

The finds’ dispersion makes it clear that human activity was carried out in hearth-focused spaces (Fig. 8; Supplementary Text S3, Supplementary Figs. S27–S32). In III-b/d and III-i/j1, most remains scatter outwards of the hearths, across the space situated between them and the bedrock shelf bounding the sedimentary infill to the North, while the hearths themselves are positioned at a constant distance (~ 2 m) from the back of the shelter. These patterns are suggestive of the inhabited space being structured into sleeping/resting (against the wall) and work/consumption (external) areas^26,40^. Within the latter, no specialized task areas can be discerned, as revealed by the spatial co-occurrence of items used to carry out the different activities that use wear analysis was able to identify.

A similar organization probably pertained in the case of III-i/j2-3, but certainty is precluded because the inhabited surface was truncated by erosion in row 19 of the grid. From the microstratigraphic configuration of Hearth H6’s charcoal-and-ash spread we can nonetheless infer the lighting in short succession of three different fires, while the associated large limestone Levallois core corroborates that stone tool making was being carried out in its periphery (Supplementary Text S1.1.2, Supplementary Figs. S4 and S7–S8).

The hearth-centric distribution of the remains, particularly evident in the case of the lithics, shows that the spatial structuration was little, if at all affected after abandonment. In the case of III-i/j1 and III-i/j2-3, most refits are short-distance and, apart from the minor disturbance of their ash caps, the fire features are in near-pristine condition. This evidence suggests that (a) the inundation events that buried layer III-i/j’s living floors were gentle (even though entailing localized erosion, as seen in the i/j2-3 lens), and (b) we are dealing with single, short-lived (i.e., no more than a few days long) occupation events. Indeed, lengthier, or multiple visits would have entailed increased levels of trampling caused by the activity and movement of personnel across the inhabited space, implying a much wider scattering of the remains and the erasure of the layers’ fire features (set as they were, on loose beach sands).

With regards to layer III-b/d, it is clear, though, that the event that buried and slightly eroded Hearth H1 entailed some displacement along the slope, explaining the accumulation found in the westernmost part of the trench, in connection with discontinuous, stretched-out lenses of charcoal and black organic matter (Fig. 8; Supplementary Figs. S4 and S27). This syn-depositional disturbance would have erased the spatial signature of specialized activity areas (if any existed, which is unlikely, given the evidence from the underlying, well-preserved occupations); the artefact and faunal assemblages from III-b/d can therefore be considered as a spatially homogenized distribution and, hence, as representative of the range of behaviors associated with the occupation(s) that generated them.

Within layer III-i/j, the refit links show, for both lenses, that the excavated area can be divided in two parts: the A and B zones of the III-i/j1 lens, and the i/j2 and i/j3 clusters of the III-i/j2-3 lens (Fig. 8; Supplementary Figs. S29 and S31). The overlap of the distributions is limited, each is spatially associated with one or the other of the two hearths found in each of those lenses, a few links between the two parts exist in both cases (including two-way refits, i.e., ones that link débitage retrieved in both clusters and extracted in non-consecutive steps of the reduction sequence of the same core, e.g., Refit #45; Fig. 8; Supplementary Text S2.1.2, Supplementary Fig. S31), and the range of documented activities is the same. This structuration suggests that, in each case, we are dealing with the remains left behind by two distinct social units (e.g., two families, or two different teams of hunters) occupying the site at the same time and cooperating in the execution of the different tasks revealed by the technology and use-wear of the stone tools.

### Site function

The study of the fauna^39^ used a layer framework, and so the finds from the i/j1 and i/j2-3 lenses were counted together. The remains (in layer III-b/d, MNI = 13; in layer III-i/j, MNI = 25) are largely dominated by red deer, followed by horse and ibex. They are anthropogenic, as shown by their burning and processing marks. The latter reveal that the different steps of the butchering sequence—e.g., evisceration, skinning, filleting—were carried out at the site. Carnivore modification is negligible (it was observed on four specimens only, i.e., on 0.6% of the two layers’ combined total), but the possibility must be borne in mind that these assemblages subsume the odd bone brought in with the inundation sands upon which the site’s occupation took place; indeed, vertebrate remains—mostly of rabbits and microfauna brought in by their accumulator, the eagle-owl, but also a few instances of washed-in long bones of deer, including articulated limb parts—were retrieved in the archeologically sterile units of sub-complex AS5 (Supplementary Text S1.1.4).

As suggested by the predominance of chert, the size of blanks, and a number of refits, the Levallois flakes in the III-i/j2-3 assemblage (all on chert) are imports, and so are the associated chert cores, which were all introduced in advanced stages of their reduction (Supplementary Figs. S16, S18 no. 1-2, and S19 no. 10). On-site production relied on the exploitation to exhaustion of those cores, complemented by the extraction of small flakes from flake blanks and the sporadic and limited exploitation of the local resource, limestone. These features match the “provisioning of the individual”^41^ type of raw material economy. This conclusion also pertains in the case of the III-i/j1 assemblage, which, however, differs in some details; for instance, Kombewa-type cores are more abundant (debris, cobbles and hammerstones excluded, they are 8 out of a total of 259 items in i/j1 and 3 out of a total of 498 in i/j2-3), and on-site use of the method is documented by a refit (Supplementary Fig. S18 no. 6). In both assemblages, the retouch debris are consistent with the notion that the retouched toolkit is composed of imports used and resharpened on site, given the predominance, or exclusive nature of type 3 of Bourguignon’s typology^42^ (retouch debris whose dorsal surface features scars denoting previous generations of retouch) (Supplementary Text S2.2.3, Supplementary Table S6).

The assemblage from III-b/d presents a somewhat contrasting pattern. Even though some of the blanks may have been imported as “personal gear”^43^, on-site production of Levallois flakes made on the most exploited lithic raw material, the locally available limestone cobbles, is documented (Supplementary Fig. S17). The presence of retouch flakes of types 0–3 shows that the entire retouching process occurred on site, from the shaping of the initial cutting edge (types 0 and 1) to the different phases of retouch (types 2 and 3) (Supplementary Text S2.2.3, Supplementary Table S6). This pattern suggests for layer III-b/d a “provisioning of the place”^41^ type of raw material economy.

The available seasonality data derive from the analysis of the dental microwear and mesowear patterns observed on red deer dentitions and from the faunal assemblage’s foetal/neonatal remains of deer and ibex (no dental cementum analyses were carried out because of the technique’s destructive nature and the small size of the potentially usable sample)^39^. In this regard as much as in terms of use wear, III-i/j1 and III-i/2-3 are very similar (Supplementary Text S1.1.4, S2.3, and S3.2–S3.3). Filleting and hide-processing are the most common tasks, ones that were carried out on the fresh carcasses of deer hunted in wintertime (a summer indicator exists in III-i/j1, but it consists of a loose upper molar retrieved in a peripheral cluster of small, patinated bones and represents background noise; Supplementary Fig. S30). The evidence comes mostly from i/j2-3, but i/j1 shares the same predominance of animal tissue- over wood-processing tools.

The faunal remains from III-b/d are characterized by a high degree of breakage, and feature few (< 1%) cut marks^39^, which accords well with the limited use-wear evidence for meat-cutting tasks in the layer’s stone tool assemblage. The III-b/d seasonality proxies indicate human activity in winter, spring, and summer, but the number of finds is inconsistent with months-long residency; going by the link between winter and filleting *cum* hide processing revealed by the III-i/j occupations, the limited importance of such tasks in III-b/d can thus be taken to indicate that the site was mostly used during warm-weather times of the year, namely during the spring, when green wood, much easier to work, would have been readily available for the making or repairing of various types of equipment (Supplementary Text S3.1).

Among the remains of red deer (in layer III-b/d, MNI = 4; in layer III-i/j, MNI = 11), the meatier bones are underrepresented, but two lines of evidence show that whole carcasses were introduced: the presence of foetal remains, which implies the evisceration of complete carcasses in at least five cases of ibex or deer from layers III-b/d and III-i/j^39^; and the use-wear evidence reviewed above, which shows that fresh hides were processed at the site. This pattern suggests that the most nutritious parts were exported. In this context, the differences observed between layers (lengthier stays, more bone breakage, and less cut marks in III-b/d than in III-i/j) probably concern not the mode of introduction but the mode of preparation of the meat for deferred consumption elsewhere: mostly limited to basic quartering in layer III-b/d, but including much filleting in layer III-i/j (the latter, speculatively but conceivably related to preservation via smoking or air-drying)^44^ (Supplementary Tables S10–S11).

## Discussion

The evidence reviewed above indicates that the different units of sub-complex AS5 record the following sequence of events: (a) the III-i/j2-3 lens contains the remains of a single, short winter visit; (b) the III-i/j1 lens contains the remains of another single, short winter visit (Hearth H2 and Hearth H5) atop an inundation lens that formed during the preceding season (late summer/early autumn) and buried the remains (primarily, Hearth H4) of a sporadic incursion; (c) the III-b/d layer subsumes a small number of short visits spanning winter, spring and summer, over an indeterminate time span, conceivably a single year, but mostly during springtime. In the course of the III-i/j winter events, the hunted game was filleted, the acquired hides and the meatier carcass parts were exported, and the tools left behind by the hunters mostly consisted of imported, ready-made tools and blanks discarded after exhaustion. In the case of the III-b/d primarily spring visit(s), we are dealing with lengthier stays that made a lot more use of locally available limestone in stone tool making, focused on wood-working tasks, and more intensively fragmented the butchered prey.

Likely, the variation in the patterns of carcass processing and the economy of stone tools seen across the Cueva Antón sequence are functionally related to the activity profiles revealed by use-wear analysis, which in turn would seem to be underpinned by seasonality. With regards to the latter, it must be stressed here that the reliability of the inferences allowed by the Cueva Antón data is warranted by the representativeness of the proxies used: the foetal/neonatal remains alone stand for a minimum of five deer or ibex individuals (the five females responsible for their presence), i.e., for at least 23% of the MNI of the two taxa in layers III-i/j and III-b/d combined^39^. This is significantly better than in even recent dental cementum studies of palimpsest assemblages, a case in point being the Mousterian fauna from the Spanish cave site of Covalanas, where, for the two main game taxa, red deer and aurochs/bison, the corresponding percentage is at best 11% (i.e., if, unrealistically, each of the teeth whose dental cementum was readable represented a different individual)^29,45^.

Rather than different settlement-subsistence systems, the units of human behavior recorded in the AS5 sub-complex are therefore best seen as different poses of a single system, one whereby the site was sporadically occupied (i.e., transiently, and then only when the configuration of the alluvial plain allowed human access). Each time, fires were lit and relit as needed, providing a reference for the division of the space between sleeping/resting (inward, along a two-meter-wide space against the back wall) and working (outward) areas. This structuration matches expectations derived from the ethnographic record^26,46^ and contributes strong and direct evidentiary support for similar inferences drawn from the spatial patterns observed at less resolved contexts of later Paleolithic age. Well-known examples of a comparable spatial structure preserved in palimpsests time-averaged over long intervals are: Tor Faraj, in Jordania, the Abric Romaní, in Catalonia, or La Folie, in France^40,47,48^, for the later Middle Paleolithic; and the Abri Pataud, in France, Badanj, in Herzegovina, Klithi and Kastritsa, in Greece, or Bacho Kiro, in Bulgaria, for the Upper Paleolithic^26,49,50^.

Despite the variation in site function and seasonality, lithic technology remained basically the same through the Cueva Antón sequence; when the assemblages are compared, it becomes clear that the differences relate to raw material economy and attendant import–export patterns. These observations shed light on one of the most protracted controversies of Paleolithic archeology, namely the interpretation of inter-assemblage variability in the Middle Paleolithic of western Europe. While late twentieth-century debates emphasized such factors as ethnicity, functionality, chronology, or blank reduction^51–54^, subsequent research has highlighted the importance of the constraints posed by raw material availability in the context of mobility and the procurement of subsistence resources^55–59^—a view that the evidence from Cueva Antón and other recently well-dated and well-studied MIS 5 Iberian contexts provides strong support for.

At Cueva Antón, the factor that explains most variation in assemblage composition across the sequence of occupations preserved in layers III-i/j and III-b/d is the extent to which imported versus locally available raw materials were exploited; otherwise, the débitage methods that were in use (Levallois and, occasionally, Kombewa) and the types of retouched tools that were made and discarded (sidescrapers, denticulates, points) are identical even if site function and season of occupation were quite distinct. The evidence from the succession of palimpsest-type occupations excavated at Abrigo de la Quebrada, in Valencia, and Gruta da Oliveira and Gruta da Figueira Brava, in Portugal,^11,60–62^, all of which span many millennia, if not tens of millennia, is consistent with this conclusion. None of the assemblages recovered at these sites fits the definition of any of Bordes’ Mousterian industry types, and it is the acceptance of such a Bordesian framework that has underpinned most past interpretations of Mousterian variability as driven by culture diversity, change through time, or activity facies.

Based on these data, we suggest that, through MIS 5, the Middle Paleolithic industries of Iberia stand for a rather stable technological system anchored on the use of the Levallois method to extract blanks with the desired morphological characteristics, and that such a system was retained irrespective of raw material, season of occupation, type of activity, duration of stay, or prey that was targeted. Indeed, regional (distribution of raw material sources), situational (residential or logistic type of occupation), and individual (composition of the personal toolkit one moves around with) factors would seem to explain most of the observed variation. These factors determine the extent to which chert items are reduced, recourse is made to the production of non-chert blanks, and alternative débitage techniques are used.

Our conclusions are supported by consideration of the Gibraltar contexts whose age is broadly the same: Upper Area B of Vanguard Cave, and the SSL and LBS members of Gorham’s Cave^11,63,64^. The former is a single, short occupation event that produced a hearth-focused scatter of stone tools, animal bones, and mussel shells akin to Cueva Antón’s living floors, while the latter are typical cave palimpsests averaging out behavior over an indeterminate, large number of visits spanning thousands of years of site usage. Yet, in terms of both the economy and the technology of their stone tools, Vanguard’s single-event and Gorham’s palimpsest assemblages are no different: raw materials are local, on-site production of quartzite blanks using the Discoid method is predominant, and imported Levallois blanks on chert are present. This pattern has been interpreted as revealing an expedient approach to lithic technology, reflecting limited mobility across a small territory^63^. Alternatively, given the similarity in raw material economy, and based on the seasonality signal provided by the Vanguard mussels^65^, we might suggest that the Gorham’s record reflects long-term springtime usage in the context of annual rounds that encompassed much larger territories. Indeed, cherts from sources 80–100 km away are represented through the Quebrada sequence since its late MIS 5 basal levels^62^, suggesting mobile lifeways consistent with the hypothesis that other, coeval cave palimpsests reflect transient, recurrent usage in the context of stable, durable settlement-subsistence systems rather more than long-term residency.

## Conclusion

Behaviorally, the patterns of spatial structuration, raw material procurement, seasonality, mobility, and functional specificity of site usage revealed by the Cueva Antón living floors are indistinguishable from those observed in the archeology of the Upper Paleolithic of eastern, southern, and western Iberia, as documented by the well-preserved, hearth-focused activity areas of mid-Gravettian chronology excavated in the stratified sites of Lagar Velho, a rock-shelter (the EE15 occupation surface), and Olga Grande 4, a Côa Valley open-air site (layer 3 of the sequence)^66–68^. Consistent with the rapidly accumulating evidence that Middle Paleolithic Neandertals possessed a fully symbolic material culture^9,12,13^ and, in comparable environments, exploited the same range of prey as the peoples of the African Middle Stone Age and the European Upper Paleolithic^69^, the settlement-system evidence reported here further contributes to the closing of the behavioral gap once thought to set them apart from recent humans. Our results provide strong support for the notion that models derived from ethnographically documented hunter-gatherers provide appropriate frameworks for the study of Middle Paleolithic/Middle Stone Age peoples, and this irrespective of their classification in terms of the taxonomic categories of Human Paleontology.

## Materials and methods

This study analyzed the totality of the stone tool assemblage produced by the 1991 and 2007–2012 field seasons (N = 2616). Assemblage integrity was assessed based on systematic intra- and inter-level refitting. Spatial analysis was based on horizontal and vertical scatterplots of the distributions of remains, coupled with the extent, orientation and composition of refitting units and refitting connections. Due to the high degree of fragmentation of the faunal assemblage, no systematic search for refits and conjoins could be carried out for the animal bone remains, Technology was interpreted based on refits and attribute analysis, under the premises of the *chaîne opératoire* approach. Use-wear analysis was performed with a Leica Wild M3C stereomicroscope equipped with two wide angle, 10 × /21b lenses and a magnification adjustment knob with five positions for each lens. In addition, we used an Olympus BHMJ reflected-light microscope with a revolving nosepiece and 50 × to 500 × objectives. This microscope features Nomarski-DIC type Interferential Contrast—allowing for high resolution and high definition, akin to that offered by scanning electron microscopy. Statistical analysis of the relationship between use-wear and tool morphology used RStudio 3.5.1, and was based on the following quantitative parameters, taken with a digital caliper: length, width, thickness, and angle of the cutting edge. For information on the methods and techniques used to acquire the evidence that our spatial analysis builds upon, readers are referred to the corresponding publications^32–39^. Additional detail on methods and an extensive presentation of our results are provided in Supplementary Materials.

## Supplementary Information

Supplementary Information.

## Data Availability

The finds from the Cueva Antón excavations and the field documents from the 2007–2012 excavations are in storage at the Museo de Arte Ibérico El Cigarralejo in Mula, where the analyses were carried out and which also provided logistical support. All data are available in the main text or the supplementary materials.
