# Supplementary material for: Organization of residential space, site function variability, and seasonality of activities among MIS 5 Iberian Neandertals
Source: Sci Rep. 2022 Nov 23;12:20221. doi: 10.1038/s41598-022-24430-z (PMC9684422; doi:10.1038/s41598-022-24430-z)
Supplement: Supplementary file 1 — Supplementary Information. [file 41598_2022_24430_MOESM1_ESM.pdf]

scientific reports

Supplementary Materials for

Organization of residential space, site function variability and seasonality of activities among MIS 5 Iberian Neandertals

Marianne Deschamps, Ignacio Martín-Lerma, Gonzalo Linares-Matás, João Zilhão*

* Corresponding author. Email: joao.zilhao@campus.ul.pt

This PDF file includes:

Text S1 to S3

Figures S1 to S32

Tables S1 to S11

Supplementary References

Supplementary Text

S1. Materials and Methods

S1.1. The site

S1.1.1. Stratigraphy and dating

Cueva Antón (Murcia, Spain) is a cave/rock shelter located on the right bank of the Mula River, *ca.* 3-4 m above the stream bed and between 351 and 359 m a.s.l. (above modern sea level) (Fig. S1). The site lies at the entrance to the El Corcovado gorge, which construction of the La Cierva dam submerged in the 1940s. Once, that gorge was one of the shortest routes for animal and human transit to and from the Mula Basin, the limestone plateaus northward and, beyond, the Central Meseta southward of Albacete, where several Middle Paleolithic open-air localities are known (1).

The site's setting explains why the bulk of the sedimentary accumulation corresponds to a 3-m-thick Upper Pleistocene fluvial terrace: complex AS (Archeological Succession) (Fig. S2). This deposit is overlain by the DD (Dam Deposit) complex and in turn overlies the basal FP (Fine Palustrine) complex. The latter is an archeologically sterile, fine-grained deposit of unknown age; the former is made up of up to 1 m-thick silts accumulated during episodes of complete submersion by the dam lake. The succession was first revealed in Zone I, which corresponds to a 3×3 m trench open in the context of a salvage operation carried out in 1991. The other trench opened at that time, Zone II, corresponds to a 2×1 m trench that cut through post-dam deposits only. Excavations resumed in 2007-08 and 2011-12, at which time the Zone I trench was expanded as shown in Fig. S2. In the early autumn of 2012, a massive flash-flood event and the ensuing prolonged submersion of the site caused a major collapse of trench walls and fieldwork had to be suspended indefinitely. Detailed descriptions and discussions of excavation history, stratigraphic layout, site formation and paleoenvironmental background are provided in several previous publications (2–7).

The remains of multiple episodes of Middle Paleolithic occupation are found in the AS complex, which comprises five subdivisions, named AS1 to AS5 from top to bottom. The texture and structure of these sub-complexes vary as a function of how the position of the back wall of the site changed relative to the fluctuating river channel. Fleeting visits represented by very small assemblages of faunal remains, wood charcoal and stone tools are found in layer I-k of sub-complex AS1, which dates to *ca.* 37 ka (thousands of years) ago, and in layer II-l of sub-complex AS2, which dates to the later part of MIS 5a (5). More substantial human occupation episodes — living floors, typically organized around hearths (Figs. S3-S9) — are recorded in layers III-b/d and III-i/j of sub-complex AS5. Based on OSL dating coupled with paleoenvironmental constraints, sub-complex AS5 was deposited in the early part of MIS 5a, during GI (Greenland Stadial) 21, which dates to the 77.8-85.1 ka interval (8).

Over most of the excavated area of AS5, layer III-e/h — an archeologically sterile package of laminated inundation sands capped by a carbonate crust — separates layer III-b/d, which contains the site's Hearth 1 (excavated in 1991), from underlying layer III-i/j, which contains the site's Hearths 2 (excavated in 1991), 3 (excavated in 2011) and 4-6 (excavated in 2012). In the NW parts of grid units M-N/18-19, however, that intermediate unit is missing (Figs. S4-S5). Coupled with the moderate dip of the stratification, this circumstance explains the (minimal) overlap between the vertical scatter plots for layers III-b/d and III-i/j shown by Fig. S10 in rows N and O of grid column 19 (see below).

S1.1.2. Combustion features

Hearth 4 (Figs. S3, S6) is the best-preserved fire feature. At excavation, it appeared as a circular area revealing the stratigraphic outline typical of *in situ* combustion. As observed in the cross-sections cut in the field and corroborated by the analysis of soil micromorphological thin sections (7, 9), the feature is capped by a thin horizontal layer of light-grey silty sediment resulting from the weathering of ash and denoting the bare ground surface upon which the fuel burned. Underneath, there is a red-rimmed, basin-shaped volume of blackened sediments representing not the infill of an excavated feature but the subsurface burning of the alluvial, laminated sands upon which human occupation took place.

Hearth 5 (Figs. S3, S6) was found 1 m to the East of Hearth 4. In this case, the partial erosion of the ash layer and the presence of injection features show that post-occupation flooding entailed underwater turbation and affected to some extent the integrity of the inundated ground floor. This is the pattern typically found in the hearth features of sub-complex AS5: *in situ* preservation, with partial, low-energy disturbance of microstratigraphic patterns caused by the inundation events that buried the archeological remains. Indeed, available photographic records show that Hearth 1 (in layer III-b/d; Fig. S5) and Hearth 2 (in layer III-i/j; Fig. S6) of the 1991 trench are akin to Hearth 5 —singular, *in situ* fireplaces that underwent minor post-depositional disturbance.

Hearth 3 (Fig. S7) was affected by more intensive erosion processes. As a result, half of the original basin-shaped volume of burned black sediment was truncated, and the feature's ash and charcoal content was partly dispersed downslope. These processes explain the well apparent, *ca.* 2 m-long stretch of organic matter and small charcoal particles dripping westward of the feature.

Hearth 6 (Figs. S7-S8) was an extensive, well-delimited accumulation of charcoal, ash, and black-stained sediment (the few blocks that at first sight contribute to the structuration of the area lie atop the accumulation and stand for a roof fall event post-dating the occupation; Fig. S4). Within, small patches of the thin horizontal layer of light-grey silty sediment typical of *in situ* hearth features could be observed. A few (5-8) centimeters below, the surface of the underlying (and archeologically sterile) laminated sands making up layer III-k/l displayed three distinct, separate red spots (designated as 6a, 6b and 6c; Fig. S8). These observations suggest that the "Hearth 6" charcoal-and-ash spread corresponds to the archeological signature of three spaced fires made within the same general area and in short succession. The patchy preservation of "pure ash" accumulations can be explained as the outcome of the human-induced, but limited perturbation caused by the activity involved in the lighting of each one of those fires.

S1.1.3. Vertical and horizontal layout

In 2011-12, three subdivisions, designated III-i/j1, III-i/j2 and III-i/j3, were made within layer III-i/j. The III-i/j1 subdivision concerns a scatter of stone tools and animal bones associated with Hearths 4 and 5, in the upper part of layer III-i/j. This scatter overlies a second one found at the base of III-i/j, across all of the then-excavated area, and wherein belong III-i/j2 and III-i/j3. The two scatters are stratigraphically well-differentiated and each is herein designated as a "**lens**:" the III-i/j1 and III-i/j2-3 lenses.

In cross-section view, Hearth 5 appears clearly separated from Hearth 4 below by a few centimeters of laminated sands (Fig. S3). Based on this observation, we can be certain that the assemblage retrieved in the III-i/j1 lens of 2011-12 is a palimpsest of consecutive occupations separated by at least one inundation event. A continuous record bridging Zone 1 with its 2012 extension is lacking, but Hearths 2 and 5 feature a similar type of limited, localized post-depositional disturbance (Fig. S6). Therefore, it is likely that Hearth 2 occupied a similar stratigraphic position to Hearth 5: at the very top of layer III-i/j, above the laminated sands that buried and preserved Hearth 4. Based on this observation, the finds from layer III-i/j of the 1991 field season were assumed to belong in the III-i/j1 lens.

The basal scatter of finds in III-i/j was also associated with two fireplaces, Hearths 3 and 6. Despite their contiguity and identical elevation, it was noticed, at the time of excavation, that an area largely devoid of finds existed between them; therefore, it could not be excluded that they stood for a horizontal stratigraphy, i.e., for occupations taking place on the same surface but at different times and so, in the field, the corresponding finds were differentiated into a III-i/j2 "**cluster**," for the material associated with Hearth 3, and a III-i/j3 "**cluster**," for the material associated with Hearth 6 (Fig. S7). Eventually, refitting demonstrated that the two clusters were coeval and that the III-i/j2-3 lens represented a singular occupation event.

Note that, along the M-O19>20 profile, essentially corresponding to grid units N-O/19, the structure of the deposit as exposed immediately to the north, around Hearth 6, changed somewhat abruptly and included areas in which lamination was apparent. This pattern was interpreted as reflecting local post-depositional disturbance of the basal part of the III-i/j2-3 lens and so this area was dealt with separately: after the excavation of the III-i/j1 lens and prior to the excavation of the III-i/j3 cluster (Fig. S4). This circumstance explains why, in the field, the very few finds made in N-O/19 at the base of layer III-i/j were labelled "III-i/j2."

Most likely, however, they stand for reworked material originally discarded at the site in the context of the occupation represented by the III-i/j3 cluster.

S1.1.4. Seasonality

The faunal remains from layers III-b/d (NISP = 219; MNI = 13) and III-i/j (NISP = 402; MNI = 25) have been studied (10). The assemblages are largely dominated by red deer, followed by horse and ibex; a few roe deer remains are also present. Rhinoceros teeth and a single bear tooth were also found in III-i/j. The cut marks found on the faunal remains show that several steps of the butchering sequence were conducted on site. Cut marks are particularly well represented in III-i/j; they are much rarer in III-b/d, probably because of the latter assemblage's increased rate of fragmentation. Burning was observed in 6% of the III-b/d bones and in 13% of the III-i/j bones.

These faunal assemblages are anthropogenic. It cannot be excluded, however, that some of their individual components (e.g., the isolated bear tooth) stand for a natural environmental signal related to the dynamics of sedimentary accumulation. In this respect, it is important to bear in mind that vertebrate remains were found through the alluvial sequence, even in those units that lacked any evidence for actual human use of the place. Most such remains were of rabbit and microfauna (rodents, birds), or of their accumulator (the eagle owl), but long bones of deer, both loose and articulated, were also found (in e.g. layers II-p/q/t, at the base of sub-complex AS3, and II-u and II-y, at the top of sub-complex AS5 (4, 10)).

It must also be borne in mind that the deposit preserved inside the cave/rock shelter of Cueva Antón is but the portion of the paleo-landscape that survived the subsequent incision of the river valley and attendant geomorphological transformations: at the time of occupation, the present-day archeological site was part of an extensive, braided-channel plain that was eventually lost to erosion except where the overhang protected it (Fig. S1). Occasionally, depending on the position of the stream channel, the sheltered area that nowadays corresponds to the archeological site was accessible and could be used by humans. Most of the time, it was inaccessible or under water; when such was the case, remains of human and animal activity taking place out in the open, upstream or in front of the site, could nonetheless end up in the excavated sedimentary infill, washed in alongside the sand and gravel accumulated by the floods. As the human occupations recorded in III-b/d and III-i/j took place atop inundation sands, it is therefore entirely plausible that the animal bones retrieved therein include some background noise, i.e., a residual component made up of pre-existing, naturally accumulated material. Thus, the study of any given unit's animal bones, even in the case of those units that are archeologically fertile, must always keep in mind the potential role of alternative agents of accumulation, both biological (the natural predators of the prey species present) and geological (the river).

With regards to seasonality, the faunal assemblages provide us with two sources of data: the remains of foetal/neonatal individuals, and the dental microerosion patterns (10). The foetal/neonatal remains are all artiodactyls, most probably deer (if not entirely; however, only one, a mandible with erupting milk teeth, could be determined to species, *Cervus elaphus*). Under the assumption that deer are borne in May, as they do today in southern Iberia, the foetal/neonatal bones retrieved in layer III-b/d (N = 12) and in lens III-i/j2-3 (N = 3) provide a reliable seasonality signal. With regards to dental microerosion patterns, we have data for ibex, horse, and rhino, but it is only in the case of red deer that a clear seasonality signal can be derived because of their feeding behavior. Even though there is indirect evidence suggesting some susceptibility to annual variation in rainfall (11, 12), the direct evidence provided by excrement suggests that, in Iberia, red deer are predominantly browsers in the winter and predominantly grazers in the summer (13). Restricting our analysis of dental microerosion to this taxon, our sample sizes are as follows: III-b/d, N = 4; III-i/j1, N = 3; III-i/j2-3, N = 2.

Given the above, the available information can be summarized as follows:

- In the III-i/j2-3 lens, we have three foetal bones; these remains indicate winter killing of pregnant females, possibly the adults that, based on the microerosion displayed by two teeth, reveal a browsing diet consistent with winter.
- In the III-i/j1 lens, we only have dental microerosion evidence, which indicates winter in two cases and summer in the third. The latter case, however, consists of a loose upper molar that comes from grid unit

I20, which is peripheral to the distribution of the unit's lithics (see Fig. S30 below; and note that the very small number of bones yielded by this grid unit includes fragments bearing clear signs of alluvial transport). In all likelihood, this remain is background noise, as per the caveat discussed above, and the more so because the information derived from carcass processing, stone tool economics, and lithic use-wear patterns suggests that human usage of the place was akin to that recorded in lens III-i/j2-3 and so that occupation must have taken place at the same time of the year (see below). Under the assumption that, under the climate conditions similar to present revealed by the paleoenvironmental evidence, major flooding would have occurred in late summer to early autumn, as it does today (the *gota fría* season), the evidence from III-i/j1 would represent a winter occupation (Hearths 2 and 5) taking place on sands accumulated during the previous late summer/early autumn inundation event — that which buried Hearth 4 and would have brought in the few remains of a riverside thanatocoenosis represented by the I20 cluster. Given Hearth 4's excellent preservation, it is unlikely that much time elapsed between the occupation it stands for and the subsequent inundation of the site, and so we can surmise that said occupation probably took place in the preceding months, sometime between winter and summer of the year before the occupation represented by Hearths 2 and 5.

- In the III-b/d layer, the foetal/neonatal evidence indicates: winter to late winter in the case of five remains representing at least two individuals; spring in the case of six remains minimally representing one individual; and summer, probably July, in the case of the mandible with erupting milk teeth, which represents a two to three-month-old individual. The dental microerosion data for four adult teeth indicate that we have two pairs representing non-consecutive seasons, one when browsing was dominant — the same time of the year indicated by the winter foetal/neonatal remains — and the other when grazing was dominant — the same time of the year, summer, represented by the mandible with erupting milk teeth.

S1.2. The analyses

S1.2.1. Research questions

Based on the previously acquired data, summarized above, our research addresses site structure and site function. These issues require assessment of the singular or multiple nature of the occupations represented by the different layers, lenses and clusters recognized at the time of excavation. Specifically, the following questions concerning the individualization of occupation events require an answer prior to proceeding to interpretation:

- Does Hearth 6 represent a singular occupation episode indeed, in the context of which three different fires were lit in short succession, or does the presence of three distinct fire *foci* (the 6a, 6b and 6c areas of subsurface reddening) indicate instead a palimpsest of three different visits, all of which organized around the same central point, perhaps because of site-wide spatial constraints?
- If Hearth 6 stands for a singular occupation, does Hearth 3 stand for a different activity area of the same occupation, or does it stand for a different occupation? We know from the seasonality data that Hearth 3/cluster III-i/j2 stand for winter (based on dental microerosion) and that such is also the case with Hearth 6/cluster III-i/j3 (based on the foetal/neonatal data), but, as a whole, does the III-i/j2-3 lens represent a palimpsest of (a) two, if not more winter visits to the site or (b) two spatially segregated activity areas (the individual III-i/j2 and III-i/j3 clusters) of a single winter visit?
- Minimally, the stratigraphic relationship between Hearths 4 and 5 implies that two winter occupations are represented by the material subsumed in the III-i/j1 lens, but does Hearth 2 relate to the occupation represented by Hearth 5 and the associated 1991-excavated finds, or does it represent yet a third occupation (and, if the latter, was it also a winter one)?
- In layer III-b/d only one combustion feature was found, Hearth 1, and the seasonality data indicate occupation in winter, spring, and summer, but was this a single continuous or near-continuous event of months-long site usage, or does this layer stand for a palimpsest of multiple occupations at different seasons of more than one year (and, if the latter, did site function vary with season of occupation)?

To advance our understating of these issues and obtain a complete and more accurate picture of human usage of the site during MIS 5a, we undertook systematic refitting and use-wear analysis of the stone tool assemblages. In the following, we explain the methods used, provide a detailed report of our results, and conclude with a discussion that combines them with the previously available information summarized above (seasonality; spatial patterning of lithic and faunal scatters; number and position of hearth features).

S1.2.2. Collections

A detailed description of the field methods employed is available elsewhere (4–5). During the two excavation phases lithics were piece-plotted in three dimensions and the excavated sediments were dry sieved on site using a 2 mm mesh. A complete flotation column of square I20 was obtained in 2011. The occupation horizons richest in charcoal fragments excavated in 2012 were also entirely floated.

The items collected during on-site dry sieving were inventoried and added to the database in 2018. In order to reduce the number of characters and thusly simplify the marking of these micro-finds, we established a parallel numbering system for small flakes, which let us include them in our systematic refitting sessions.

The lithic assemblages from layers III-b/d and III-i/j contain a total of 2618 finds (Table S1); 675 of these were piece-plotted in three dimensions; 1941 were collected following the dry sieving protocol.

S1.2.3. Refitting

The search for refits and break conjoins among the lithic remains was initially carried out within each layer as it was judged that, excluding very localized disturbances caused by erosion of the small-gully kind, related to sedimentation dynamics, the very good preservation of fireplaces was a strong indicator that whatever post-depositional movements had occurred occur did so in a relatively limited manner. Nonetheless, we also tested the stratigraphic integrity of the lithic assemblages from each of the units recognized at the time of excavation via the systematic search of refitting links between sub-complex AS5's different layers, lenses and clusters.

Refits were inventoried, and the spatial distribution of items was analyzed using two-dimensional projections along the different axes of the grid (Figs. S9-S14). Given their important number (137 of 240 refitted pieces), the items retrieved through dry sieving were also included; to do so, we assigned them approximate coordinates (center of ¼ m² sub-square for X and Y coordinates and average spit altitude for the Z value). In order to better perceive and demonstrate the vertical differentiation of occupations within layer III-i/j, the different items were projected vertically on both the X and Y axes using 50 cm-thick slices (Figs. S12-S13). This protocol was followed in order to avoid cumulating slope effects, which would have resulted in a distorted, vertically dilated representation of the distributions. For these purposes, two piece-plotted flakes from 1991 and five items from 2011-12 labelled "i/j1" in the field that came from the interface with, and refitted with material from i/j2-3 (one core, one flake and three debris; see section S3.1.1. below) were treated as belonging in the latter lens.

To understand the spatial organization of remains and their relationship with the various hearth structures, we produced horizontal planimetric (X-Y plane) projections (Fig. S11) and used rose diagrams and statistical analyses to look into the possible relationship between the preferential orientation of refit units and the dip of the stratification (Fig. S14). Firstly, we used the Rayleigh Test of Uniformity to test the significance of the mean as indicating a preferential orientation distribution (General Unimodal Alternative). We then applied the Kuiper Test of Uniformity to support the significance of Rayleigh's test in those cases when a multimodal distribution was possible. Finally, we applied Watson's Test for Circular Uniformity, which helps to confirm an anisotropic pattern by testing if the sample is homogenous or if there are preferential trends. These tests offer slightly different insights and, in combination, provide cross-validation of the inferences drawn from each. The rose diagrams were produced with OpenStereo (14) and the associated statistics used the Circular library (15) in R (16).

S1.2.4. Economy and technology

Once taphonomic biases were controlled for we carried out a techno-economic analysis of the lithic assemblage aiming at situating the site, both spatio-temporally and functionally, within settlement/activity

cycles via evaluation of import/export patterns, differential raw material management, and degree of tool consumption. We began by reconstructing, to the extent possible, the operational chains represented and therefore identify which operational schemes were used (Figs. S15-S20; Tables S1-S6; for the interpretation of the graphs in Fig. S15, note that the technological category percentages consider both retouch and unretouched blanks; if bearing unambiguous diagnostic markers, flake fragments and small, <2 cm flakes were also considered; otherwise, they were excluded from the calculations).

For several reasons outlined below, the study of raw materials has remained relatively limited, and only generalized categories, such as chert, limestone, or sandstone could be used (Fig. S15; Table S2). While it is true that the study of imported raw materials provides information on the geographical range of procurement territories (regardless of whether such ranges are representative of the movement of people or objects), at the time being extra-local cherts lack characterization, meaning that their precise origins remain unknown. The local chert has, however, been well characterized, though it needs to be borne in mind that chert sources 80 km away yield quite similar material and the two potential proveniences remain of difficult differentiation (17). The limestone that was used for knapping is immediately available in the form of river cobbles.

The technological analysis used both refitting and an analysis of technical attributes indicative of an item's position in a given reduction sequence. These data were collected as they provide information on the phases of reduction executed at the site (18, 19), thereby allowing investigation of how production was spatially segmented within and between sites. Combined with raw material provenance, these data provide further insights into the spatial segmentation of production and the circulation of personal gear (18, 20, 21). The retouched toolkit was studied technologically to assess blank selection, providing information on the morphology of the cutting edges that were desired and their intended cutting angles, and allowing us to situate them within the operational chains employed. When present, retouch flakes, classified with the system proposed by L. Bourguignon (22), were looked into as a proxy for the intensity of on-site resharpening.

S1.2.5. Use wear

Most lithics could be retrieved from the unconsolidated sands that packaged them in almost completely matrix-free manner. Given this fact and the pristine preservation of edges and surfaces, piece-plotted and dry-sieved items were bagged in the field, with their labels, and were stored unwashed. To improve the readability of the traces, they were lab-cleaned for the purposes of the use-wear analysis carried out in the context of this study, using an ultrasound vat.

The objects were analyzed with a Leica Wild M3C stereomicroscope equipped with two wide angle 10×/21b lenses and a magnification adjustment knob with five positions (0.64×, 10×, 16×, 25× and 40×) for each one of three lenses (0.32×, 0.63× and 1.6×). We used episcopic lighting from two cold light beams produced by an Intralux 4000 adaptor. The stereomicroscope is equipped with a Wild 308700 camera and a photographic adaptor for digital reflex cameras and computer interfacing. Depth-of-field issues were fixed using EOS Utility and Heliconfocus. This kit was applied to the study of edge chipping, typically recorded at 6.3× but resorting to greater magnifications when required for detailed observation.

In addition, we used an Olympus BHMJ reflected-light microscope with a revolving nosepiece and 50× to 500× objectives. This microscope features Nomarski-DIC type Interferential Contrast — allowing for high resolution and high definition (23), akin to that offered by scanning electron microscopy (24) — and it is also connected to a digital camera for image recording. Typically, these kinds of microscopes are used at 200×; lower magnifications are used for general scanning of the edges, higher ones for features (e.g., striations) that require additional observational power.

Our use-wear results are presented in Table S7 and Figs. S21-S25.

S1.2.6. Statistics

The results obtained regarding the use of different raw materials (operational chains and their spatiotemporal organization, frequency and diversity of tools, generations of resharpening), in addition to the results obtained regarding the function of tools, constitute the backbone of our interpretations. To enhance our understanding of such task-related patterns of tool selection and tool use we ran several statistical tests on a

set made up of the unretouched blanks and the type-list retouched tools that displayed diagnostic use wear (Tables S8-S9; Fig. S26).

To select the most suitable variables and prevent the simultaneous use of highly correlated ones, we performed the Kaiser-Meyer-Olkin (KMO) factor adequacy test (0.5 cut-off value), and assessed with the Bartlett test whether the resulting correlation matrix was an identity matrix (i.e., one where all residual correlations are zero). To further secure the validity of the dataset for complex multivariate statistics, we calculated covariance homogeneity; we obtained <2 values in all but one case (an activity with low sample size), supporting the suitability of the dataset for our purposes. To visually represent the relationships between morphometric variables and use-wear patterns we did a Principal Components Analysis (PCA). These analyses used RStudio 3.5.1 (16), the libraries in (25–28), and were based on the following quantitative parameters: length, width and thickness of the object, taken with a digital caliper; and angle of the cutting edge, measured after Dibble & Bernard (29).

S2. Stone tools

S2.1. Refitting

S2.1.1. Assemblage integrity

The vertical projection of items and refits confirms the clear separation between the stone tool finds made in layers III-b/d and III-i/j, as clearly demonstrated for row 19 in Fig. S10. The connections between refitted items show a similar lack of post-depositional mixing, as a single inter-layer refit was found and the refits between items from the same stratigraphic unit are numerous (III-b/d: 72 items refitted into 33 intra-layer refit units; III-i/j1: 77 items refitted into 25 intra-lens refit units; III-i/j2-3: 95 items refitted into 33 intra-lens refit units).

The single inter-layer refit concerns a core and a flake in square M18 where the core sat at the surface of III-i/j/1 and the small flake was collected while dry sieving sediments labelled as III-b/d. This connection is simply explained by the fact that the intermediate layer III-e/h was present only in the southern half of the square (it becomes thin and, eventually, non-existent in the northern part, where instead the base of III-b/d makes direct contact with III-i/j). This small flake therefore must belong to III-i/j1, as the core does, and simply represents a small object accidentally removed while taking off the base of III-b/d to expose the surface of III-i/j (in the process, both the sediment resulting from the décapage and its sieved contents were by convention assigned to III-b/d, explaining why this apparent inter-layer link is but an unintended consequence of labelling, not an instance of genuine post-depositional vertical displacement).

In layer III-i/j, 13.5% of the lithic remains were refitted. The horizontal projections show a differential spatial distribution of the three assemblages identified during excavation (Fig. S11). The remains from III-i/j1 are located predominantly within squares J-L/18-22, are associated with Hearth 2, and correspond largely to the 1991 excavation trench. Altitudinally, the distributions of the 1991 and 2007-12 finds correlate perfectly, and several refits between the two collections were made. The remains from III-i/j2 were found principally within squares L/18-19, while those from III-i/j3 were found for the most part within squares M-O/18-19.

In order to investigate the relationship between the three III-i/j assemblages, we conducted vertical projections of the refits on the X (Fig. S12) and Y (Fig. S13) axes, again by 50 cm slices. The projections on the X-axis confirm distinct organizations that follow the general slope (roughly 20° on the East-West axis). The III-i/j1 and III-i/j2 point clouds are superposed, notably in the eastern part of the site (Fig. S12B-C), while the III-i/j2 and III-i/j3 point clouds form vertically contiguous but horizontally distinct distributions (Fig. S12E). Fig. S13 shows the same information on the Y-axis; the superposition between III-i/j1 and III-i/j2 is particularly visible in Fig. S13E, while the vertical contiguity of III-i/j2 and III-i/j3 is just as evident as on the X-axis (Fig. S13F-H).

A small number of refit units link III-i/j items that were field-assigned to different lenses/clusters. This is to be expected because (a) in the absence of a clear stratigraphic marker that the excavation could follow, the separation between III-i/j1 and the underlying clusters could not but be approximative, and (b) the low-energy

inundation event that buried each occupation must have entailed the displacement along the slope of at least a few of the smaller items discarded in the context of that occupation, thereby incorporating such items in the sand deposit packaging the remains of the occupation that came next. For analytical purposes, namely with regards to economy, reduction sequence and production technology, the material included in these mixed provenience refits therefore needed to be assigned to one or the other of the stratigraphically meaningful units, the III-i/j1 and III-i/j2-3 lenses. The color codes used in Figs. S12-S13 to indicate provenience (point plots and refit links) already reflect such a post-excavation reassignment, which was carried out as explained in the following.

Seven refit units (#41, #45, #51, #52, #53, #54 and #65) link 22 finds excavated as III-i/j2 or III-i/j3 to eight finds labelled as III-i/j1 in the field: they are cores (2), flakes (3), and debris (3). One of the cores (L20-30) and two of the flakes (one from L20, lacking piece-plotting information, the other with row 21 as the only provenience information) are from the 1991 excavation; the core plots in a manner that is consistent with the adjacent III-i/j2 cluster as defined in 2011 and such would most certainly have been the case with at least the flake from the same square, had it been piece-plotted too. The other core (J18-87) comes from a ledge on the bedrock situated at the topographic interface between III-i/j1 and III-i/j2; even though assigned to the former in the field, the refit links indicate that it belonged to the latter. The other flake (L19-117) is, at 350 cm below datum, the lowermost item assigned to III-i/j1 in that square, where many items assigned to III-i/j2 in the field were retrieved between 351 and 353 cm. These observations suggest that (a) even though one is justified in correlating with the III-i/j1 lens of 2011-12 the 1991 material from layer III-i/j as a whole, the latter must also include a few items that belong in the III-i/j2-3 lens, especially where square L20 is concerned, and (b) the five items from 2011-12 that refit with material from III-i/j2-3 but had been field-assigned to III-i/j1 belong in the former, not the latter (i.e., they reflect not post-depositional disturbance but rather "excavation error;" in this case, the uncertainty inherent to the stratigraphic assignment of items retrieved at the interface between units that lay in direct contact).

S2.1.2. Spatial structuration

The spatial distribution of remains (Figs. S27-S32) clearly indicates that the occupation surfaces were only partially excavated. For example, the important accumulation of remains and the concentration of refit links in squares O/18-19 of layer III-b/d suggest that an important concentration extending southward to the wall and westward along the slope was only partially sampled during the 2012 excavation season. It is the same for the III-i/j1 lens, as made apparent by the fact that Hearths 4 and 5 are cut in half by the M-O19>20 stratigraphic profile, while the III-i/j3 cluster extended northward, as shown by the truncation of Hearth 6 by the M-O18>17 profile.

With regards to layer III-b/d, no combustion feature appears to be associated with the O/18-19 concentration, which, in accordance with its downslope location relative to Hearth 1, might therefore represent an accumulation of finds displaced by post-depositional disturbance. Alternatively, it could represent (a) a second activity area of the occupation represented by Hearth 1 or (b) a distinct occupation, conceivably related to combustion feature(s) found in unexcavated grid units O/20-21. With regards to III-i/j1, it is noteworthy that the areas north- and westward of Hearths 4 and 5 yielded a limited number of finds, which indicates that the unexcavated parts of the occupation surfaces that these combustion features belong to are in grid units M-O/20-22. Finally, the issue with III-i/j2-3 is whether each of the two clusters differentiated in the field represents a distinct activity area of a single occupation or two distinct occupations of the same surface.

In order to further investigate these alternatives, we need to consider three sources of evidence: (a) the horizontal distribution of the finds across the excavated areas (Fig. S9); (b) the dip of vertical scatter plots and refit links relative to the natural dip of the stratification (Figs. S10-S13); (c) the distances separating the different items included in individual refit units (Figs. S27, S29, S31); and (d) the predominant direction of refit links, as revealed by the rose diagrams in Fig. S14 and statistically validated by the tests made on the distributions of the actually piece-plotted elements. The Rayleigh test shows that the mean direction of refit orientations is representative of the preferential orientation for the three cases considered. The Kuiper test reinforces these results, namely with regards to multimodal distributions, while the Watson test confirms the anisotropic nature of the distributions.

The horizontal distribution of the lithics in layer III-b/d is distinctly bimodal (Fig. S9). Nevertheless, it seems that (a) the concentration around the hearth in K/20-21 presents mostly long-distance connections with the second concentration at the base of the slope (>1 m) rather than short-distance connections around the hearth (Fig. S27), and (b) the majority of conjoins and refits concern two artefacts only. The rose diagram (Fig. S14) presents a preferential E-W orientation, though the N-S one is also significant. These orientation patterns are consistent with the natural slope of the layer, and are parsimoniously explained by post-depositional disturbance of the original distribution. Therefore, we must conclude that the archeologically observed pattern no longer represents an undistorted reflection of that extant at the end of the occupation.

In III-i/j1, a roughly unimodal distribution is visible around the upslope hearth located in squares K/20-21 (Hearth 2), which is in the same position as the overlying layer's Hearth 1 (Fig. S9, S29; in Fig. S9, note that the bubble in N18 corresponds to a total weight of 336 g, of which 332 g represent a single limestone core). Hearth 2 was subjected to visible erosion similar to that which is visible in Hearth 5 at the base of the slope (Fig. S6), and it is likely that both are coeval and their disturbance simultaneous (see above). Two refitting observations support such a notion: firstly, long distance refits connect remains found around Hearth 2 with remains found around Hearth 5; secondly, these refits follow the slope of the unit and are oriented along the dominant orientation (NE-SW) revealed by the rose diagram.

In contrast with III-b/d, most III-i/j1 refit links are short distance, which suggests the presence of a well-preserved spatial structure. Hearth 2 would seem to have been the focal point of the occupation, with Hearth 5 bounding it to the West. In addition, the fact that 85% (623 out of 732) of the III-i/j1 lithics come from columns J, K, L, and M of the grid suggests that the assemblage overwhelmingly relates to the occupation represented by those two hearths. The earlier occupation represented by Hearth 4 may have been very short, leaving no more than a limited number of remains, following a model of exceedingly transient usage akin to that documented at the site by layers II-l and I-k. Located higher-up in the sequence, these layers were extensively excavated but featured stone tool densities of <1 per m²: 34 items over 35 m², in layer II-l, 20 items over *ca.* 54 m² in layer I-k (4, 5). Based on this reasoning, we can infer that (a) even though extending into the unexcavated parts of the III-i/j1 lens in grid units M-N/20-22, the occupation represented by Hearths 2 and 5 was excavated over *ca.* 80% of the surface then occupied (some 22 out of about 28 m²), and (b) the excavation trenches contain the core area over which the remains of the associated human activity were discarded and within which they underwent minor post-depositional displacement.

Along the E-W axis, the surface of the III-i/j2-3 lens abutted the outcropping bedrock to the East, dipped markedly to the West in adjacent columns J-K (13 cm over 1.5 m), and then became nearly horizontal in columns L-O (where the dip is of 15 cm over 4 m). Reflecting this difference, Hearth 3, located in J-K/19, underwent significant erosion caused by the low-energy inundation event that buried the archeological remains, while Hearth 6, located in M-N/18, was much less affected (Fig. S7). That inundation event must have caused a degree of post-depositional downslope displacement, as indeed exemplified by the longest distance refit that we could make (Fig. S31); this refit connects one item of the III-i/j2 cluster found against the outcropping bedrock at the eastern edge of the occupied surface (J18-87, a limestone core; 34.6 g) with two small flakes from the III-i/j3 cluster (O19-194, 3.1 g; O19-203, 7.3 g).

Otherwise, the evidence from the III-i/j2-3 lens is that (a) we are dealing with a distribution that, for all practical purposes, must be considered unimodal (Fig. S9) (b) the confidence interval for the mean orientation of refit connections is much wider than in III-i/j1 or III-b/d (c) the predominating orientation is N-S, across the largely horizontal stratigraphic layout of the lens, not E-W, along the slight, natural dip of layer III-i/j as a whole (Fig. S14), (d) most refits are short-distance and intra-cluster, with a few short-distance links connecting the two clusters (Fig. S31). The tight within-cluster relationship found in III-i/j2-3 is exemplified by a group of four III-i/j2 refit units made on the same raw material and scattered over only some 3 m² in grid units L-M/18-19. In addition, the scatter plot of the III-i/j3 cluster and the distribution of its refit connections define an area that surrounds Hearth 6, directly atop which few lithics were found. These observations lead us to conclude that (a) the three reddish spots (6a, 6b and 6c) identified below the ash-and-charcoal scatter at the center of the III-i/j3 cluster (Fig. S8) reflect the lighting and relighting of fire in the same general area and in the context of a singular occupation taking place around it, and (b) the III-i/j2 cluster represents a distinct activity area, organized around Hearth 3, of that same occupation.

The spatial distribution per order of extraction of the items making up refit unit #45 (Fig. S31) further strengthens the conclusion that the III-i/j2 and III-i/j3 clusters represent the remains of a single occupation. The final extractions and subsequent discard of the core took place in the area surrounding Hearth 6 to the South and West (i.e., in the III-i/j3 cluster). However, two blanks (the second and the fourth in the extraction sequence) were found on the opposite side, in the space between Hearths 6 and 3 (i.e., in the III-i/j2 cluster). This pattern cannot result from the natural processes known to have been in action at the site (namely, post-depositional displacement as a result of inundation) because those two items were found upslope from the knapping area. Therefore, human agency must be assumed. In a two-episode scenario, however, human agency fails to pass the Occam's Razor test. If Hearth 6 stood for the earlier episode, then, to access the area upslope where Hearth 3 is found, people coming to the site at a later time would have trampled Hearth 6 and the associated III-i/j3 cluster of finds, erasing a spatial structure that, instead, is neatly preserved. If the earlier episode were to be that structured around Hearth 3, items 2 and 4 could represent transport by a person from the knapping spot to the use and discard spot, or exchange between the knapper and another member of the group (and item 4, a Levallois flake, is indeed an intended product); in that case, however, the distribution of the other items around a hearth feature that did not exist yet is hard to explain, unless if, by remarkable coincidence, the people who came after lit the fire features making up Hearth 6 in the exact spot where that earlier knapper had been working. Clearly, the parsimonious view of the evidence is that (a) the two clusters result from activities taking place during one and the same visit to the site and (b) the two burnt items retrieved within the area of Hearth 6 reflect actions (knapping, producing splinters; or tossing, of unwanted material) that took place while the fire was alight.

S2.2. Operational chains

The three archeological assemblages, III-b/d, III-i/j1 and III-i/j2-3, have been validated by the spatial and taphonomic studies. This in turn allows us to conduct a comparative technological analysis in order to identify operational similarities and differences.

S2.2.1. Raw material management

The ways that materials were brought to the site for each of the analyzed assemblages represent distinct economic choices. We have observed clear differences between layer III-b/d, on one hand, and the combined lenses of layer III-i/j, on the other.

In layer III-b/d, limestone outnumbers chert (Tables S1-S2 ; Fig. S15), indicating an acquisition strategy whereby local raw materials were more heavily exploited, perhaps because the III-b/d assemblage represents an occupation of different length, or a palimpsest of several occupations. The combined mass of the lithics from III-b/d is three times that in III-i/j2-3 (Table S2). The amount of chert introduced, however, does not exceed 1.5 kg per occupation, underlining that this material was introduced in small quantities; yet, when considering the number of individual artefacts, chert dominates the III-i/j1 and III-i/j2-3 assemblages. When the mass data is treated as percentages (Fig. S15), limestone would appear to have been introduced in similar proportions; yet, by number of individual artefacts, we observe considerable inter-assemblage variation. The true difference shown by these numbers is that limestone use is significant in layer III-b/d and much more sporadic in lenses III-i/j1 and III-i/j2-3.

Finds made of sandstone are comparatively rare and consist mainly of cobbles and rare flake fragments.

S2.2.2. Blank production

S2.2.2.1. Layer III-b/d

Nineteen cores were collected in layer III-b/d. Eight of these present traits consistent with the Levallois concept, both in terms of design and the organization of scars. The recurrent-centripetal method seems to have been principally employed, but one core presents scars that are consistent with the preferential method. The early stages of production sequences exploiting the ventral surface of flakes are visible on three other cores, but these are short sequences that cannot easily be assimilated to a specific concept; they could represent intentional extraction using the naturally convex ventral surface around the bulb or the setting-up of

a Levallois-type reduction sequence. Seven other cores, of which six are of limestone, display only a handful of removals organized in centripetal manner.

Our technological analysis (Fig. S15) identifies clear differences between chert and limestone flakes, mostly concerning the presence or absence of cortex. Chert products rarely preserve cortical surfaces, yet these are quite common among the population of limestone flakes. This is indicative of relatively complete, on-site operational chains for limestone, and of comparatively truncated production chains for chert. Another interesting point resides in the documentation of a Levallois production on limestone. In contrast, the elements that can be associated with toolkit maintenance (e.g., retouch flakes) are mostly of chert, as also are the overwhelming majority of retouched tools. Moreover, chert Levallois flakes are much larger than the removals that can be observed on the cores, suggesting that the larger Levallois products are ready-made imports and that only the smaller ones stand for on-site production (Fig. S16). The latter may have been extracted from used cores whose more productive phases of exploitation had taken place elsewhere in the landscape or, more likely, from complete nodules that were themselves small to begin with.

A single refit group (#5), including five flakes and a core, presents a relatively complete sequence of removals (Fig. S17, no. 1) whose direction is a clear example of the recurrent-centripetal Levallois method. The last refitted element is a small flake (<2 cm). During this last production step, the core eventually broke in two and was discarded.

S2.2.2.2. III-i/j1 lens

The III-i/j1 lens yielded eight cores and four core fragments, of which four fit various Levallois modalities: one is consistent with the recurrent-centripetal method, a second is consistent with the preferential method. Two cores on the ventral surfaces of flakes are also present. Four cores, two in chert and two in limestone, are barely reduced. Lastly, there is a limestone core presenting a multidirectional organization resulting from the accumulation of a number of different sequences of unidirectional production.

When we consider flakes, the differences between chert and limestone appear more pronounced in III-i/j1 than in III-b/d (Fig. S15). Cortical flakes are strongly represented among limestone examples, followed by regular flakes. No Levallois flakes in limestone were identified in III-i/j1. The significant proportion of cortical surfaces among limestone flakes indicates that the exploitation of volumes was initiated on site, while the fact that regular flakes are outnumbered by cortical flakes implies quite short production sequences. For chert, the distribution of technological categories is quite like in III-b/d, though naturally backed and *débordant* flakes are less well represented. The Levallois flakes present in III-i/j1 are, however, similar in size to the last scars visible on the cores, which is suggestive of their being produced on site (Fig. S16).

At first sight, based on the number of retouch byproducts and the percentages in Fig. S15, retouching and resharpening activities appear to have been less important in III-i/j1. However, bear in mind that small flakes are excluded from the technological category graphs in Fig. S15; when the assemblages are considered in their totality (Table S1), the difference is not statistically significant. Indeed, the apparent reduction in the number of retouched tools in III-i/j1 is an artefact of the smaller size of the assemblage; percentage-wise, there are twice as many retouched tools in III-i/j1 than in III-b/d (5.4% versus 2.7%; 7.9% versus 4.6% if debris, cobbles, and hammerstones are excluded), and the difference is statistically significant.

Refit units #50 and #59 demonstrate production sequences that occurred during the occupation of III-i/j1. Refit unit #50 (Fig. S18, no. 5) illustrates its core's last production phase: only two pieces are missing, and these could very well correspond to the target products, ones that were either exported or ended-up in a non-excavated area of the site. Refit unit # 59 (Fig. S18, no. 6) shows the exploitation of the ventral surface of a flake-core where the flintknapper took advantage of the naturally convex surface located around the bulb. Several Kombewa flakes were refitted, yet the flakes corresponding to the setting-up of the striking platform were not found (or refitted), possibly because of their small size. Only two flakes are missing from the ensemble that could be refitted onto the flaking surface itself, one of which, removed from its center, could correspond to a target product that, unlike the other refitted flakes, bore no remains of the ventral side of the core's flake blank.

S2.2.2.3. III-i/j2-3 lens

Nine cores and four core-tools were discovered in lens III-i/j2-3. Five cores have a clear Levallois design, with one demonstrating a unipolar production method and the other four demonstrating the recurrent-centripetal method. Production was initiated on the ventral surface of three flake-cores; the disposition of the removals, though few in number, evokes a Levallois-type production.

Four sidescrapers were recycled as cores (Fig. S18, no. 4; Fig. S20, no. 1-2). Three of these were exploited on their ventral surfaces, again using the natural convexity provided by the bulb of percussion, while the fourth was exploited on its dorsal surface. These recycled tools are the only elements of the toolkit presenting scalariform retouch on one of their edges. A plausible hypothesis for the decision-making process behind such recycling can therefore be proposed: after successive generations of resharpening, resulting in the scalar disposition of the retouch scars, the cutting edges of these tools were likely judged inapt for the task at hand, possibly because of an inadequate working angle or the irregularity of the edges. Consistent with a logic revolving around the conservation of quality materials, these items were recycled as cores. Such economic decisions regarding the conservation of chert were only identified in lens III-i/j2-3; in the other assemblages, discarded tools do not seem to have been recycled.

The differences observed in III-i/j1 with regards to the technical categories of flakes as a function of raw material were also observed in III-i/j2-3 (Fig. S15). Whether cortical or non-cortical, limestone flakes are all regular products. There are no Levallois flakes on limestone, even though a large Levallois core made of limestone was found adjacent to Hearth 6 (Fig. S7). The core is significantly larger than all the others found in III-i/j2-3, which leads us to conclude that it was abandoned during an early phase of reduction. In contrast, several extremely exhausted Levallois cores in chert are to be added to the aforementioned chert tools that were recycled into cores. This pattern would seem to indicate that chert was exhaustively consumed and limestone resorted to sporadically and in complementary manner only (as in III-i/j1 and unlike in III-b/d).

The different proportions of chert flake types are also quite similar to those seen in III-i/j1. Retouch byproducts and retouched tools appear more numerous in III-i/j2-3 than in III-i/j1, but the difference is not statistically significant. The Levallois flakes in III-i/j2-3 show a wider range of variation in length (Fig. S16), and some are much larger than the last scars present on the cores, which suggests that they are imports. However, the very small Levallois flakes, sometimes smaller than 2 cm, are more likely the result of on-site production.

The refit groups illustrate the presence of different production phases. Five refitted sequences demonstrate either the setting-up of surface convexities or the blocks' initiation. A single one (#45; Fig. S18, no. 1) illustrates an end-of-reduction extraction from a recurrent-centripetal Levallois core. This core (Fig. S18, no. 3) is particularly interesting because the operational chain shows a localized platform-preparation phase (refitted flakes 1 through 3), followed by the exploitation of the flaking surface using the part of the platform that had been prepared during the preceding phase (flakes 4 and 5). The flake extracted next, likely a predetermined target product, is absent. Another localized platform preparation follows (flake 7), the exploitation of this core ending with a failed production sequence — flake 8 hinges and flake 9 overshoots, removing a part of the platform — leading to discard. Despite its high degree of exhaustion and resulting small size (27×24×15 mm), the core's volumetric structure was maintained throughout.

S2.2.3. The retouched toolkit

The retouched toolkit is almost exclusively made of chert. There are only five limestone items, four in III-b/d and one in III-i/j2-3. Sidescrapers are dominant in all units, with lateral sidescrapers being the most numerous sub-type (Tables S3-S5). Partially retouched flakes come second. Denticulates and notches are documented by two specimens in III-b/d and absent from III-i/j1 and III-i/j2-3.

In all three assemblages, the blanks for the majority of the tools are Levallois flakes (Tables S3-S5), followed by flakes with cortical backs (in III-b/d) and partly cortical flakes. The majority of the refitted sequences concern the final phases of core reduction. The exceptions are two refit units in III-i/j2-3 that illustrate the starting out of whole-nodule reduction. Despite this, cortical and semi-cortical flakes are heavily represented among the retouched tools. This evidence leads us to conclude that the large cortical flakes

retouched into formal tools are imports that entered the site either as blanks or as ready-made items; their large size would give them long use lives, allowing for the successive sharpening and resharpening associated with intense raw material economization.

The different types of retouch flakes (Table S6 ; Fig. S19, no. 11-15) suggest inter-assemblage variation in the extent to which tools were resharpened or recycled. It should nevertheless be noted that the often sub-centimeter retouch flakes that constitute this line of evidence are more susceptible to fragmentation and post-depositional displacement, meaning that they are more often than not underrepresented. Retouch flakes types 0 to 3 (according to Bourguignon's typology (22)) were found in layer III-b/d, indicating that the entire retouching process occurred on site, from the shaping of the initial cutting edge (types 0 and 1) to the different phases of retouch (types 2 and 3). The retouch flakes identified in III-i/j1 are rarer and concern only type 3 flakes, i.e., flakes whose dorsal surfaces feature scars denoting previous generations of retouch. In III-i/j2-3, a diversity of types of retouch flakes are once again observed, indicating that tools were both shaped, used, and resharpened on site, though type 3 retouch flakes are more numerous, suggesting that several generations of edge resharpening occurred prior to on-site discard. These observations are consistent with the hypothesis that, in layer III-i/j, the retouched toolkit is composed of imports used and resharpened on site.

A final category of tools consists mostly of items that fit within the broad family of hammerstones. For the most part, these consist of retouchers made of small cobbles or the cortical surfaces of limestone flakes, both being rare occurrences in all three assemblages (Fig. S20, no. 6-9). While reporting on Middle Paleolithic retouchers has mostly been concerned with items made of bone, retouchers made of small cobbles have previously been identified in a few sites; such items display concentrations of impact marks that are oblong (rather than the circular ones observed on standard hammerstones used for blank production) and comparable to those observed on bone retouchers (30).

S2.3. Use wear

S2.3.1. The evidence

Out of a total of 378 retouched and unretouched blanks complete or sufficiently complete to warrant use-wear analysis, a hands-on, macroscopic examination determined that the surface condition of about 60% was not good enough to justify further examination. Of the remaining 150 that were thoroughly scanned for wear under the microscope, 55, i.e., more than one third, yielded diagnostic evidence: 26 in layer III-b/d, 9 in the III-i/j1 lens, and 20 in the III-i/j2-3 lens. These numbers translate into, respectively, 21.5%, 9.0% and 12.7% of the universe of potentially identifiable functional tools.

Twenty-eight pieces were used on carcass-processing tasks. Polishes resulting from contact with meat are of limited intensity — little more than a flat, dull, undifferentiated shine spread across the high points of the chert surface's microtopography, without a clearly defined boundary with non-polished areas. When associated with scattered areas of contact with bone or hard matter, such polishes denote use in butchering activities involving separation of meat from bone, explaining why the analyzed tool will record contact with both types of materials (Fig. S21). In the subsequent phase of filleting, contact with bone is no longer observed (Fig. S22). The striations associated with these tasks are of a similar kind: in filleting, parallel to the active cutting edge; in butchering, with a diverse range of orientations, though predominantly parallel too. If the tool is sharp and the job is done in easy and rapid manner, only a minimal amount of friction between the edge and the material being worked on is produced; that is why polishes are not very intense and the identification of tools used in butchering and filleting is often rather difficult. When, as at Cueva Antón, the size and shape of the edges allows for resharpening by retouch (e.g., so as to eliminate the blunting caused by the presence of grease), the recognition of carcass processing wears is additionally hindered; thus, the number of items so classified should always be considered as a minimum.

Eight pieces were used on hide (Fig. S23). Polishes resulting from contact with hide are more compact than those related to the processing of meat and, when more intense, feature a rounded texture. Summarizing an extensive literature (31–34), the operational chain for the processing of hides with stone tools involves the following phases: 1) detachment of the hide from the body; 2) removal of the grease and any remains of meat adhering to the inner side of a fresh hide stretched with stakes or on a frame, which requires a very sharp,

acute cutting edge; 3) optionally, depending on the intended use, depilation (which requires humidification and is often aided by maceration with ash, plant matter, urine, or animal excrement), followed by a brushing or scraping finish; 4) pseudo-tanning (rubbing or scraping of a dried skin using antiseptic materials, which achieves stabilization of the material but demands continuous maintenance), or proper tanning using organic or mineral agents that alter the chemical composition of the material and render it impermeable but rigid, thereby requiring softening via scraping and/or the addition of greasy stuffs. The original hide is thusly transformed in leather, which can be cut with sharp knife-like tools for use in the production of clothes, shoe wear, and a wide range of instruments and containers. At Cueva Antón, three items bearing on-hide use wear relate to the preparation of a fresh hide (step 2), and the remainder to the softening of a hide tanned with an abrasive substance (step 4). The processing of wet hides (step 3) has not been documented, possibly because this activity only leaves a very weak, difficult-to-identify polish (35–38).

Nineteen pieces were used on wood (Fig. S24). The corresponding wear is characteristic. It spreads from the high to the middle elevations of the microtopography of the tool's surface. When use is less intense, the resulting polish is dispersed and presents a dim and greasy shine; when use is more intense, the result is a more compact, softly rounded polish observed over a surface that is well bounded and reflects a bright and luminous light. At Cueva Antón, this type of wear appears on sidescrapers that share a certain morphological semblance and a similar working edge angle. These tools probably were used in surface-smoothing tasks related to the manufacture of shafts, hafts, and handles. The homogeneity of form and wear that characterizes the assemblage they form may reflect use on woods of the same species, in similar condition, or of comparable hardness.

S2.3.2. Stratigraphic variation

The stratigraphic distribution of the stone tools that yielded the use-wear evidence is provided in Table S7.

In layer III-b/d, the treatment of animal products is documented by nine objects (e.g., Fig. S21, nos. 2-3) that were in contact with bone, two that were in contact with meat, and another two (e.g., Fig. S23, no. 4) that were used, with an abrasive, on hide. Wood was identified as the raw material the tool was in contact with in the case of 13 pieces (e.g., Fig. S24, nos. 2-5). In one case (Fig. S25, no. 1), the location and type of the evidence, plus the size and shape of the object suggest that the on-wood wear relates to hafting.

In layer III-i/j, the treatment of animal products is documented by seven objects that were in contact with bone (e.g., Fig. S21, nos. 1, 4), ten that were in contact with meat (e.g., Fig. S22, nos. 1-3), and six (e.g., Fig. S23, nos. 1-3) that were in contact with both fresh and tanned hide. Wood-working is represented by a much smaller number of pieces, only six (e.g., Fig. S24, nos. 1, 6). In addition, this layer also yielded two objects that, in all likelihood, were used as projectile tips, as indicated by (a) their overall shape and size, (b) the absence of use wear, and (c) the presence of sets of diagnostic impact striations in areas of the tool where they cannot have been caused by knapping (Fig. S25, nos. 2-3). A Mousterian point from III-i/j2 (Fig. S20, no. 4) bears on-bone wear evidence; it may also have been a projectile tip, but no macro- or microscopic evidence of impact could be observed and so this item was counted as a butchering tool (and treated accordingly for the purposes of Tables S7-S8 and Fig. S26).

The evidence from layer III-i/j comes mostly from the i/j2-3 lens, but the i/j1 lens shares the same predominance of animal product- over wood-processing tools that sets both apart from the layer III-b/d assemblage: in the latter, wood-working represents 48% of the activities documented by use-wear analysis, as opposed to 22% in III-i/j1 and 20% in III-i/j2-3. Likewise, filleting (as documented by items with meat-only use wear) is represented by 8% of the assemblage in III-b/d but corresponds to 33% of the total of use-worn objects in III-i/j1 and to 35% in III-i/j2-3. These differences are statistically significant (Table S8).

These functional differences appear against the technological background of similarity in operational schemes observed across the three assemblages. Only very fine differences could be discerned, such as the increase in limestone Levallois production in III-b/d or the recycling of exhausted cores into tools in III-i/j2-3. These small and quite specific techno-economic contrasts do not, however, reflect distinct production concepts. From a purely typological standpoint the retouched toolkits also show very little inter-layer difference: in all three assemblages, single-edge, lateral side scrapers dominate by a significant margin. Why

did the same range of lithic raw materials, the same knapping technology and the same types of retouched tools give rise to such a marked inter-layer distinction in use wear-related patterns?

The variation in the activities documented across the three assemblages could result from:

- Sampling bias related to the placement of the excavation window relative to the spatial organization of the occupation itself; because habitation surfaces were only partially excavated it is possible, in III-b/d for example, that activities related to filleting and hide processing took place in areas located beyond the trench walls.
- A true difference in the nature of the occupations, with implications for the different activities carried out during each, potentially reflecting seasonality or even distinct modes of territory occupation and attendant variation in the degree of functional specialization.

Even though sampling bias cannot be completely excluded, a number of arguments militate against such being the explanation for the differences revealed by inter-layer comparison. Recall that (a) the overwhelming majority of the III-i/j1 lithic assemblage relates to the "Hearths 2 and 5" event, (b) we have estimated that *ca.* 80% of the area occupied in the context of that event has been excavated, (c) the III-i/j1 finds that yielded the information of relevance to assess site function (use wear and seasonality) all come from this "Hearths 2 and 5" area (Figs. S29-S30), and (d) the scatter of the III-i/j1 lithic assemblage presents a clear "around-the-hearth", tossed core-bounded pattern fully consistent with the notion that, for all practical purposes, it can be treated as the remains of a singular occupation episode centered on Hearth 2. This evidence strongly suggests that the functional profile one can infer from the III-i/j1 data is unaffected by problems arising out of the potentially biased sampling of a structured space. The same is likely to apply to III-i/j2-3 because its seasonality signal is identical and the p-value obtained when comparing both lenses' use-wear numbers is indicative of no difference at all (Table S8).

With regards to layer III-b/d, recall that the refitting study concluded that the parsimonious explanation of the evidence was that the original spatial structure of the occupation event(s) therein subsumed had been largely lost due to downslope, post-depositional dispersal. Such a process means that the remains originally abandoned within any restricted, possibly task-specific areas that may have existed eventually commingled to form a single, tendentially homogenized scatter. Thus, even if we posit that the occupied surface was sampled by the excavation trench much more partially than is the case for III-i/j1, the probability that the assemblage retrieved is representative of what was going on at the site is, conversely, much higher in III-b/d than in it is in III-i/j1.

Based on these arguments, we can conclude that the significant statistical difference (p-value = 0.025; Table S8) revealed when comparing the occupations in layer III-i/j with those in layer III-b/d has behavioral underpinnings. In short: during layer III-i/j times, the activities carried out at the site that use-wear analysis is amenable to document were mostly of the hide-processing and filleting kind; during layer III-b/d times, they were mostly of the wood-working and butchery kind.

S2.3.3. Form and function

From a typological standpoint, the toolkit appears homogenous, while use-wear analysis reveals a diverse range of activities. In order to better understand the relationship between tools and tasks, we analyzed several simple metric variables as a function of the raw material worked (Table S9; Fig. S26).

The analysis of cutting angles used on different materials highlights some differences. Firstly, the sidescrapers used to work hide tend to have cutting angles that are wider than the working edges of tools used on other materials, and this is particularly the case for those sidescrapers having been used on hides treated with an abrasive substance. On the contrary, sidescrapers that were used to scrape bone and wood tend to have narrower cutting angles. For the cutting of meat, non-retouched flakes were privileged, and these have cutting angles that are quite sharp. Only one retouched piece was used to cut meat, but the cutting angle on this tool is similar to the angles observed on the unretouched flakes used for the same task. No unretouched piece seems to have been used in hide working, however.

The links between sharp angles and the cutting of meat and between blunter angles and use in various scraping tasks (on bone, wood, or hide) are to be expected; however, they underline the robust nature of our technological and use-wear analyses, otherwise supported by multivariate analyses using the variables Length, Cutting Angle, and the Thickness/Width ratio. The KMO test result overall is 0.65, with all three variables scoring >0.6. Cutting Angle's score is the highest (0.71), as could be expected, since it is the most functionally relevant variable. The Bartlett test ($\chi^2 = 21.143$, df = 3) yielded a suitable p-value (9.831877e-05, <0.05). As five of the objects were fragments lacking length or width information, the size of the sample of use-worn tools analyzed was 50. The PCA graph (Fig. S26) confirms that the tools used for filleting and the scraping of tanned hides form rather tight and well-separated clusters that otherwise overlap only minimally with the point clouds formed by the tools used in other tasks. The tools used on fresh hides form a dispersed scatter that it would be too hazardous to interpret because of the small size of the sample. The tools used in wood working and butchery activities share the same space, although wood is the functional category that shows the greatest dispersion.

Lastly, we can clearly state that a relationship exists between certain tool uses and the morphometric characteristics of said tools. While we cannot talk of straightforward standardization, it is clear that blanks and cutting edge types (whether retouched or unretouched) were chosen as a function of the activity being carried out. On sidescrapers there is additionally a strong relationship between the working angle and the task for which the tool was used immediately before it was discarded.

S3. Spatial structure and site function

The integrated consideration of taphonomic, technological, typological, use-wear, and statistical studies let us reach a holistic comprehension of how Cueva Antón's lithic assemblages relate to site function. To advance this issue, we now turn to comparing our results on the lithics with the data available on the faunal remains.

S3.1. Layer III-b/d

The zooarcheological study (10) showed that cut marks were rare in this layer (<1 %), and suggested a link between such rarity and the assemblage's high degree of bone breakage, which would have destroyed many of the marks. However, it is interesting that these cut mark data are coupled with a nearly total absence of use-wear traces related to cutting meat. Given that, because of the disruption of this layer's original spatial distribution, the use-wear sample is representative of the activities that took place during the occupation(s), it appears likely that both lines of evidence, cut marks and use wear, be functionally related.

Stone tools with wood working wear were found across the excavated surface, and the same can be said about those having come into repeated contact with bone (Fig. S27). Based on the distributions, Fig. S27 might also be taken to suggest that filleting took place around Hearth 1, and that the scraping of tanned hides took place in the area of the O/18-19 grid units — and, therefore, that some spatial structure can nonetheless be gleaned from the data. However, because of the seasonality evidence, these patterns cannot be taken to support that the remains stand for a single occupation within which task-specific areas can still be discerned (Fig. S28). If that had been the case, the occupation would have lasted for about four months (late winter to early summer), which is inconsistent with the number of finds. For the stone tools, for instance, such a duration implies an unrealistic daily rate of on-site discard: 1 item per every 2 m² of the excavated area. Given the small, constrained space available for use at the site, such a duration would also have been unfavorable to the preservation of archeologically recognizable activity clusters.

Based on the distributions in Figs. S27-S28, a conceivable scenario is that (a) the lithic scatter around Hearth 1 primarily represents a winter occupation during which some meat was filleted, and (b) the concentration of finds in O/18-19 stands for a spring occupation, one during which tanned hides were scraped and one that was eventually post-depositionally enriched with Hearth 1-related material originally discarded upslope. Minimally, however, at least a third occupation represented by the summer proxies would still have to be contemplated. Unambiguous interpretation of the spatial relationships is therefore not possible and we have to settle for the conclusion that III-b/d is a palimpsest of multiple occupations taking place at different

times of the year, the sum product of which being a pattern whereby wood working and butchering are responsible for most of the documented use wear. If we assume that the association of filleting and hide working with winter suggested by the data from layer III-i/j is of general behavioral significance, then it is possible to propose that the preponderance of butchering and wood working signals an association of such tasks with spring and summer, and that such are the seasons of the year best represented in the III-b/d palimpsest.

S3.2. Layer III-i/j, lens i/j1

From the spatial structure of the lithic scatter (Fig. S29), it could be inferred above that most remains from the III-i/j1 lens relate to the second occupation therein documented (that organized around Hearths 2 and 5). Limited post-depositional downslope displacement, combined with the effects of human agency (e.g., the tossing of bulky stuff), suffice to explain the low-density tail of finds extending into M-O/18-19.

The scatter shows a degree of spatial structure suggesting that, as was the case with III-i/j2-3, we are dealing with two distinct activity areas. Nine refit groups were constituted around Hearth 2 (Zone A in Fig. S29) and twelve in squares J-L/17-19 (Zone B in Fig. S29), whereas only five refit links connect these two adjacent zones. These five refits are chronologically bidirectional, which supports their relative contemporaneity (39). The distribution of cores on blocks around Hearth 2 is consistent with knapping activities taking place in the two zones, on both sides of the fireplace, and use-wear analysis shows that the different activities identified are homogeneously distributed across the entire occupation surface. It would therefore appear that the two distinct areas around Hearth 2 of III-i/j1 are not specialized activity areas. Sample size is small, but the representation of wear types is identical to that seen in i/j2-3 and the season of occupation is also the same, winter (as discussed above, the isolated tooth with a micro-erosion pattern indicative of summer was retrieved outside the area of distribution of the anthropogenic finds and in all likelihood represents environmental background noise; Fig. S30).

S3.3. Layer III-i/j, lens i/j2-3

The lithic taphonomy study allowed us to demonstrate that (a) the III-i/j2/3 lens is minimally affected by post-depositional disturbance (explaining the good preservation of Hearth 6), and (b) the separation between the i/j2 and i/j3 clusters is archeologically meaningful, as each one corresponds to a distinct activity area. These findings are consistent with the observation that the stone tool scatter plots (Fig. S31) display a distribution peripheral to the Hearth 6's charcoal-and-ash spread. This pattern suggests that Hearth 6 was a focal point for activities carried out around the fires and only while they were alight, i.e., in the course of a singular occupation episode (one that, according to the seasonality evidence, took place in winter; Fig. S32).

Filleting is the predominant activity but butchery is also represented, which is consistent with the fact that 7% of the ungulate bones from layer III-i/j as a whole have been found to bear cut marks (10). Uniquely in the sequence, the i/j2-3 lens yielded evidence for the processing of fresh hides. Otherwise, the range of activities documented in each cluster is the same, except for the scraping of tanned hides, represented by a single item in i/j2 (but sample size is small, and there is no statistically significant inter-cluster difference). The parsimonious reading of this evidence is that the clusters stand for the remains left behind by two groups of people that exchanged items while doing more or less the same things.

S3.4. Summary

In layer III-i/j, the numerous lithic remains used in the cutting of meat were found in the same area as the objects used on hides prepared without an abrasive, which suggests occupations strongly linked to processing of the carcasses of freshly hunted animals. Furthermore, this layer's faunal assemblage features cut marks associated with evisceration (cut marks on ribs) and skinning (cut marks on phalanges) (10), and these steps of carcass treatment are the very first in the operational chain of butchery.

Among the remains of deer, the assemblages' best represented taxon, elements from the axial skeleton and from the lower legs are well represented in III-i/j, while bones from meatier parts of the animal seem to be underrepresented (Table S10). This could indicate that carcasses were processed on site and that the most

nutritious parts were exported for deferred consumption elsewhere. As a high number of small bones are present in both the lower legs and the axial skeleton, the relative frequency of bones can be a proxy for body part representation only in the case of those whose number in the skeleton is similar. This is the case with the long bones of the legs, e.g., the metapodials, whose quantitative importance in both layers is highlighted by the data in Table S11. However, whether we use the data in this table or in Table S10, statistical comparison shows that, with regards to the representation of skeletal parts, no significant difference exists between III-b/d and III-i/j.

The limited representation of bones from the more nutritive parts of the carcass can be explained by two alternative hypotheses:

- Such portions were exported.
- The bones from such portions were fragmented to recuperate the marrow and/or crushed and boiled to render the bone grease; it is notable that anthropogenic fracture marks were diagnosed on roughly 23% of bones in III-b/d and 30% in III-i/j.

The zooarcheological study (10) concluded that “the butchery patterns observed on deer and medium-size ungulate specimens document skinning, disarticulation, defleshing, and marrow extraction” and that “this treatment pattern is consistent either with the introduction and on-site processing of complete carcasses or with the piece-meal introduction of portions drawn from all different parts of a carcass.” Our use-wear data show that the introduction of complete carcasses is implied by the treatment of fresh skin documented by three items retrieved in the III-i/j2-3 lens. Given the similar body part representation patterns, such must be the case in layer III-b/d as well. However, the use-wear data also show that a statistically significant difference exists between the two layers in the relative proportion of tools related to butchery (with on-bone wear) and tools related to filleting (with on-meat wear only) (Table S8). It is conceivable, therefore, that a difference exists between the two layers that concerns not the mode of introduction but the mode of preparation of the meat for immediate or deferred consumption: mostly limited to basic quartering in layer III-b/d, but including much filleting in layer III-i/j (the latter, speculatively but conceivably related to preservation via smoking or air-drying).

These different lines of evidence allow us to propose that the remains conserved in III-i/j1 and III-i/j2-3 correspond to short-term winter occupations primarily oriented towards the treatment of animals hunted nearby. The situation is a bit different in III-b/d, which is a partially disturbed palimpsest of multiple short-term occupations that occurred between the end of the winter and the summer. Though it is possible that each of those occupation episodes was functionally distinct, the fact that wood working is dominant suggests a link with spring, an important season for wood growth. This time of the year would be optimal for the making or repairing of various forms of equipment made of wood as it is when green wood, which is much easier to work, becomes readily available.

Our results are strongly suggestive of a correlation between site function and season of occupation. This is consistent with numerous ethnographic examples that demonstrate how the rhythms of hunter-gatherer lifeways are influenced by the changing of the seasons, which underpins strategic decisions on how the territory is used (e.g. (40)). We show that was already the case among the Middle Paleolithic Neandertal populations of MIS 5 Iberia.

Supplementary Figures

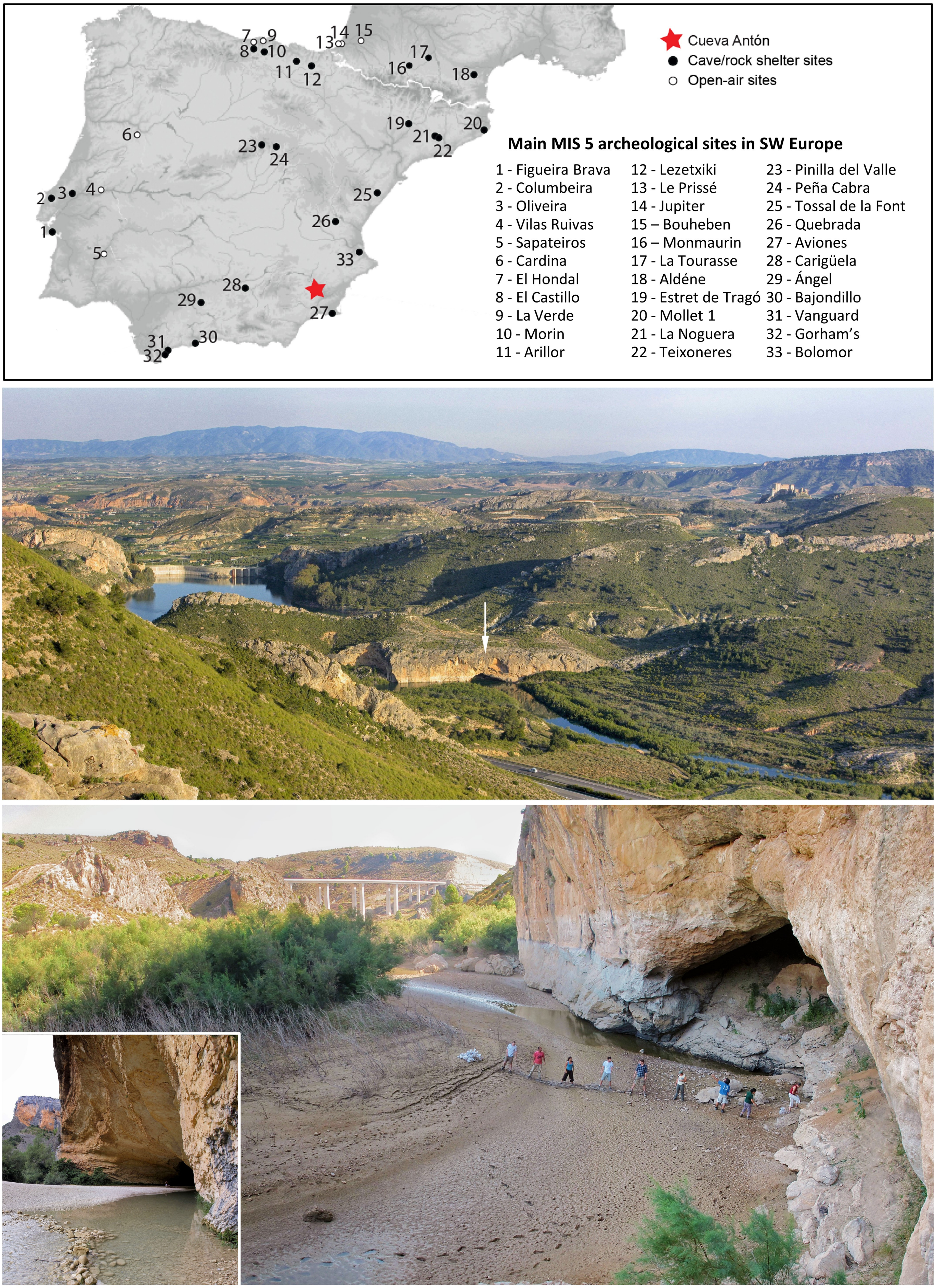

Fig. S1. Geographic context. Top. Location of Cueva Antón and main MIS 5 sites of SW Europe. **Middle.** Overview of the La Cierva dam lake; the arrow indicates the site. **Bottom.** The Mula riverbed in September of 2011, when the dam lake dried out in front of the cave; the inset is a view of Cueva Picamartillo, in the canyon of River Vero (Aragón), a modern analogue for Cueva Antón at the time of occupation.

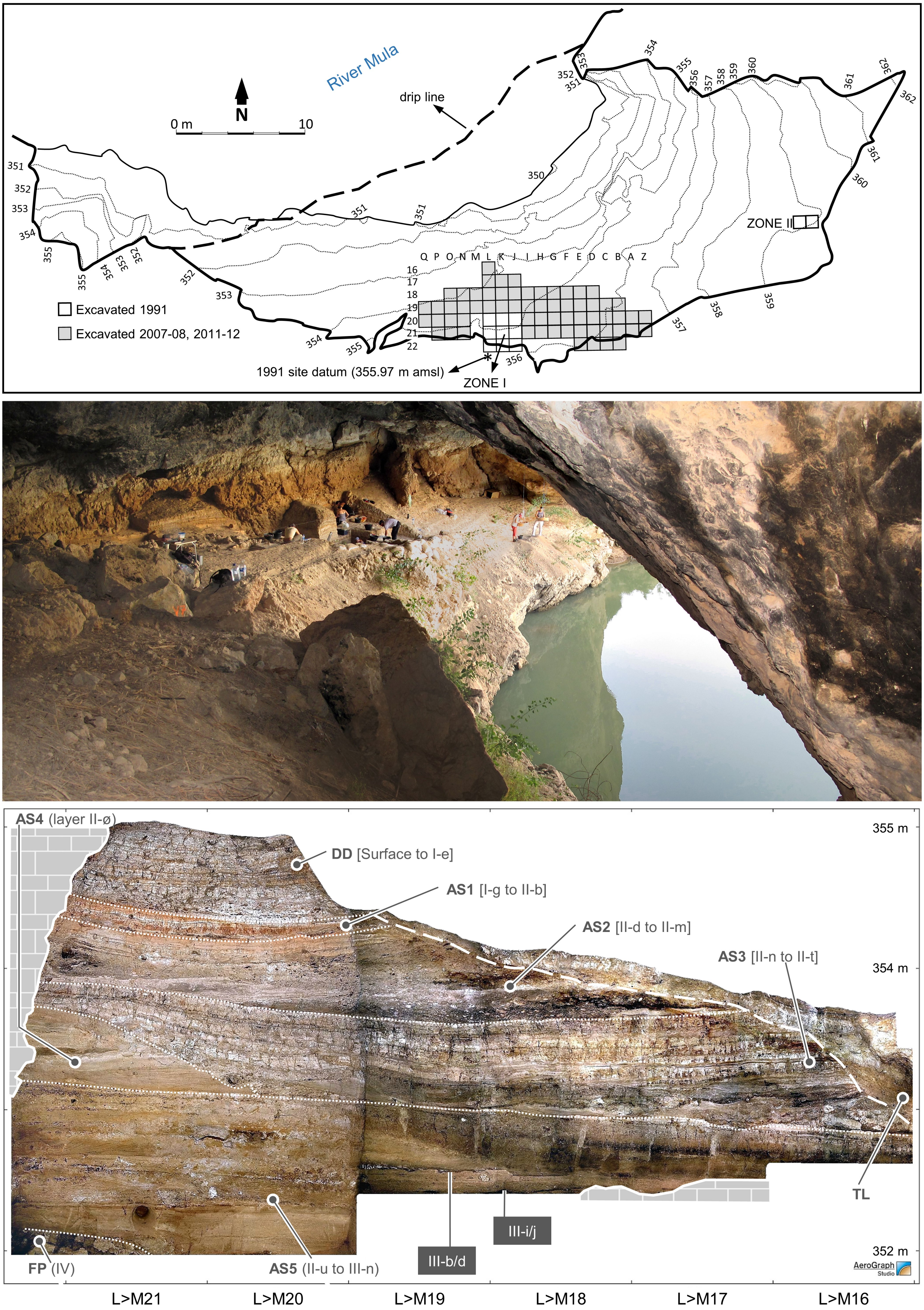

Fig. S2. The excavation. Top. Plan and site grid. **Middle.** Overview of the cave's interior, during the September 2012 field season. **Bottom.** The reference stratigraphic profile. Elevations are in m asl.

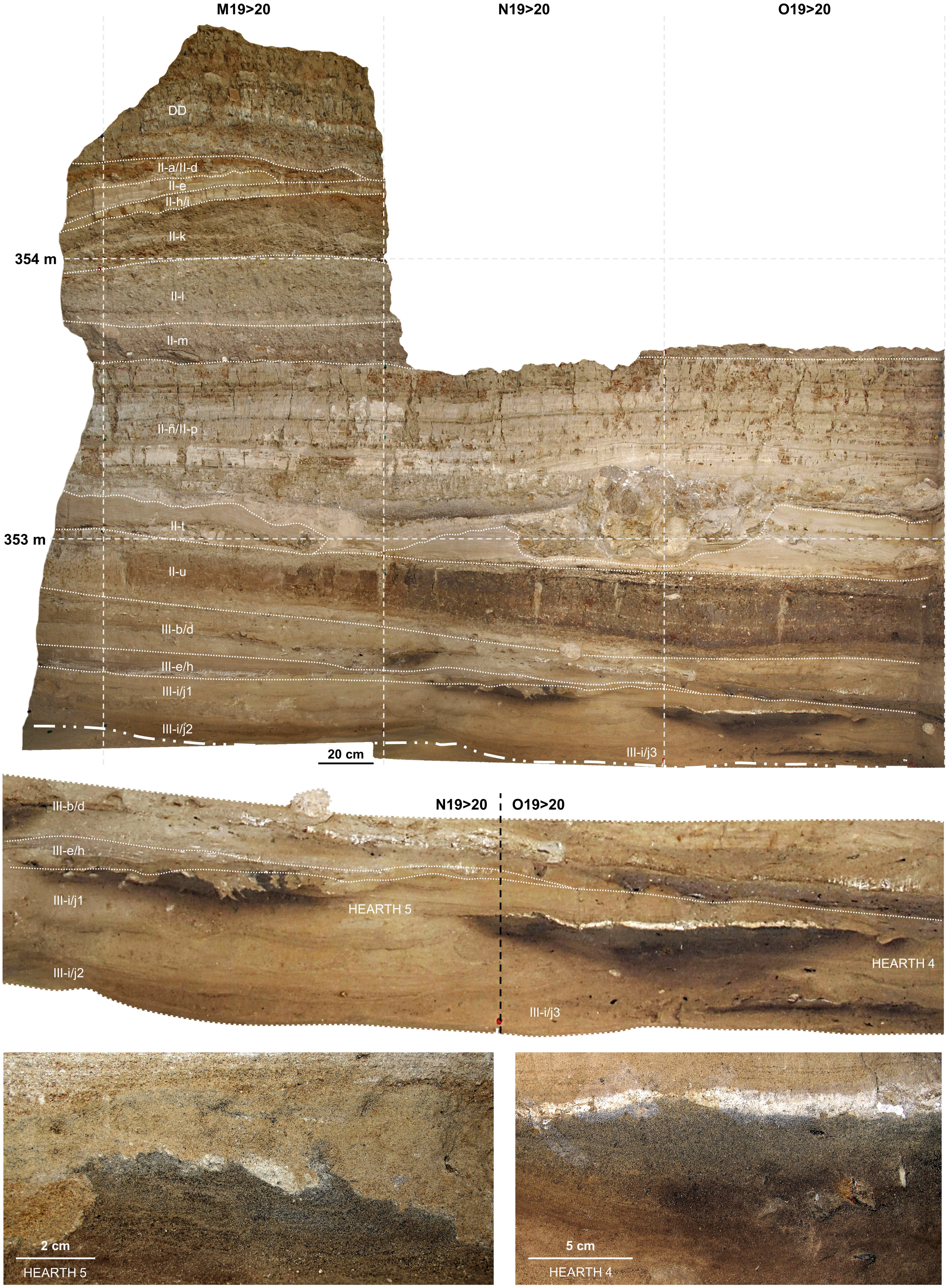

Fig. S3. South profile of the 2012 trench. Top. Orthorectified photo with stratigraphic subdivisions. **Middle.** The lower part of the profile in grid units N-O/19, illustrating the relative stratigraphic position of Hearths 4 and 5. **Bottom.** Field-macrophotography images illustrating aspects of the post-depositional alteration of Hearth 5 (left) and the largely pristine preservation of the microstratigraphy of Hearth 4 (right). Elevations are in m asl.

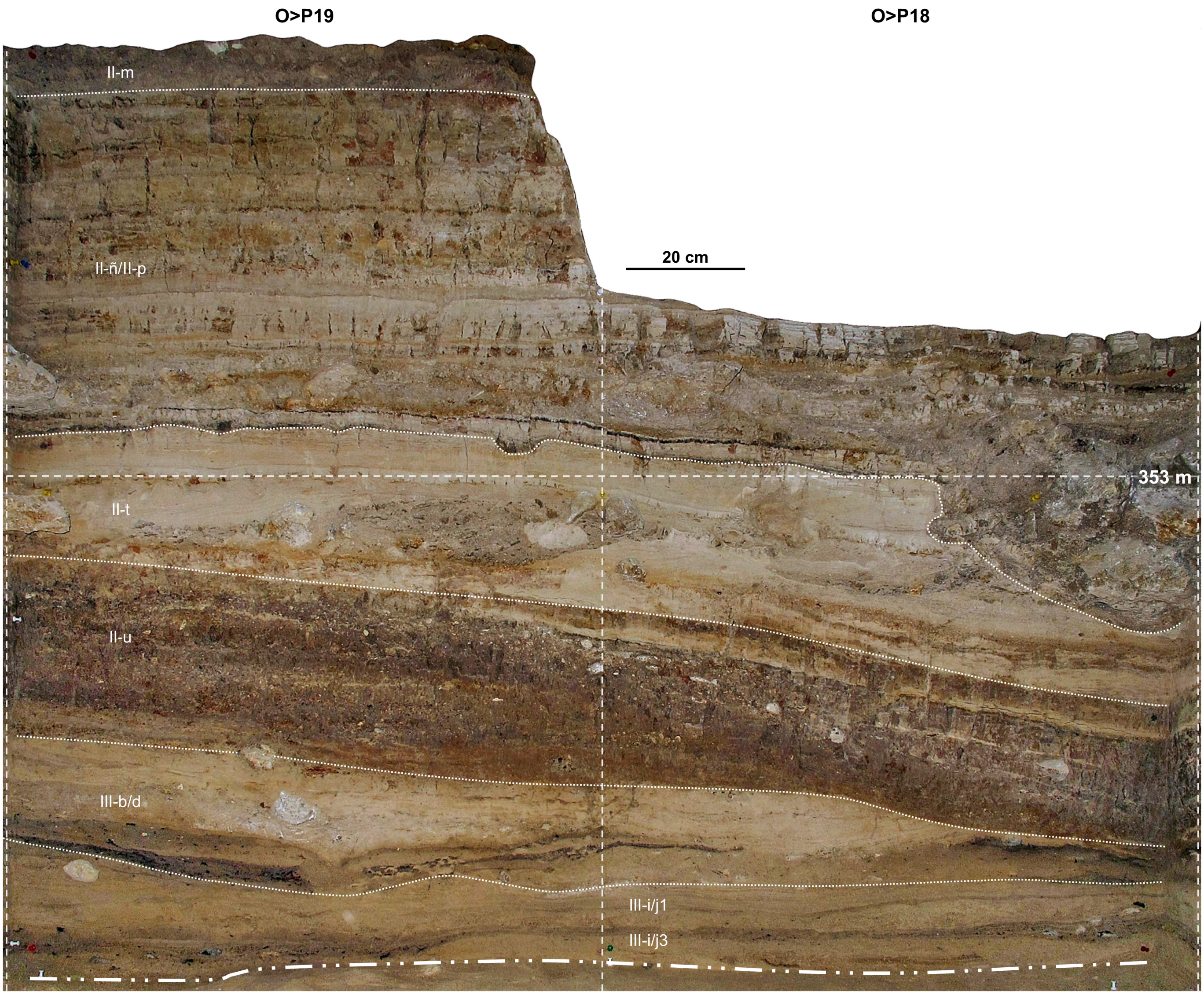

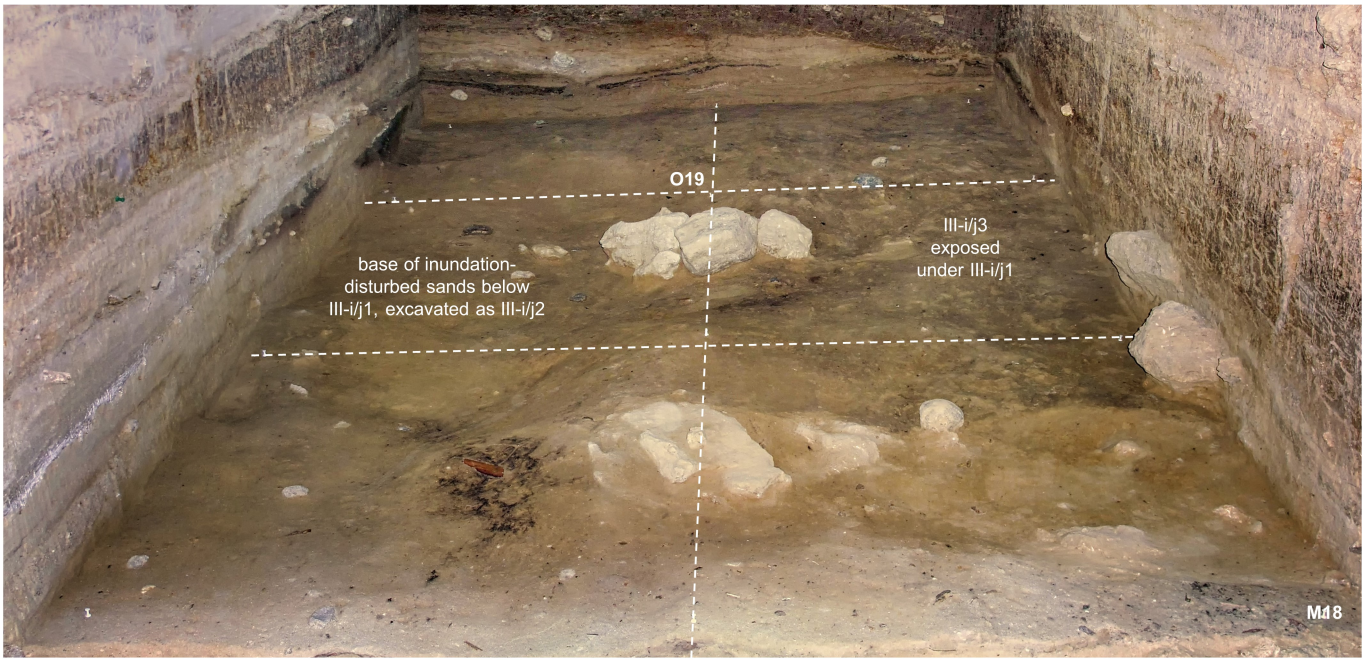

Fig. S4. The 2012 trench (squares M-O/18-19). Top. Orthorectified photo of the west profile, with stratigraphic subdivisions. **Bottom.** Oblique view of the décapage of the III-i/j3 cluster. Elevations are in m asl.

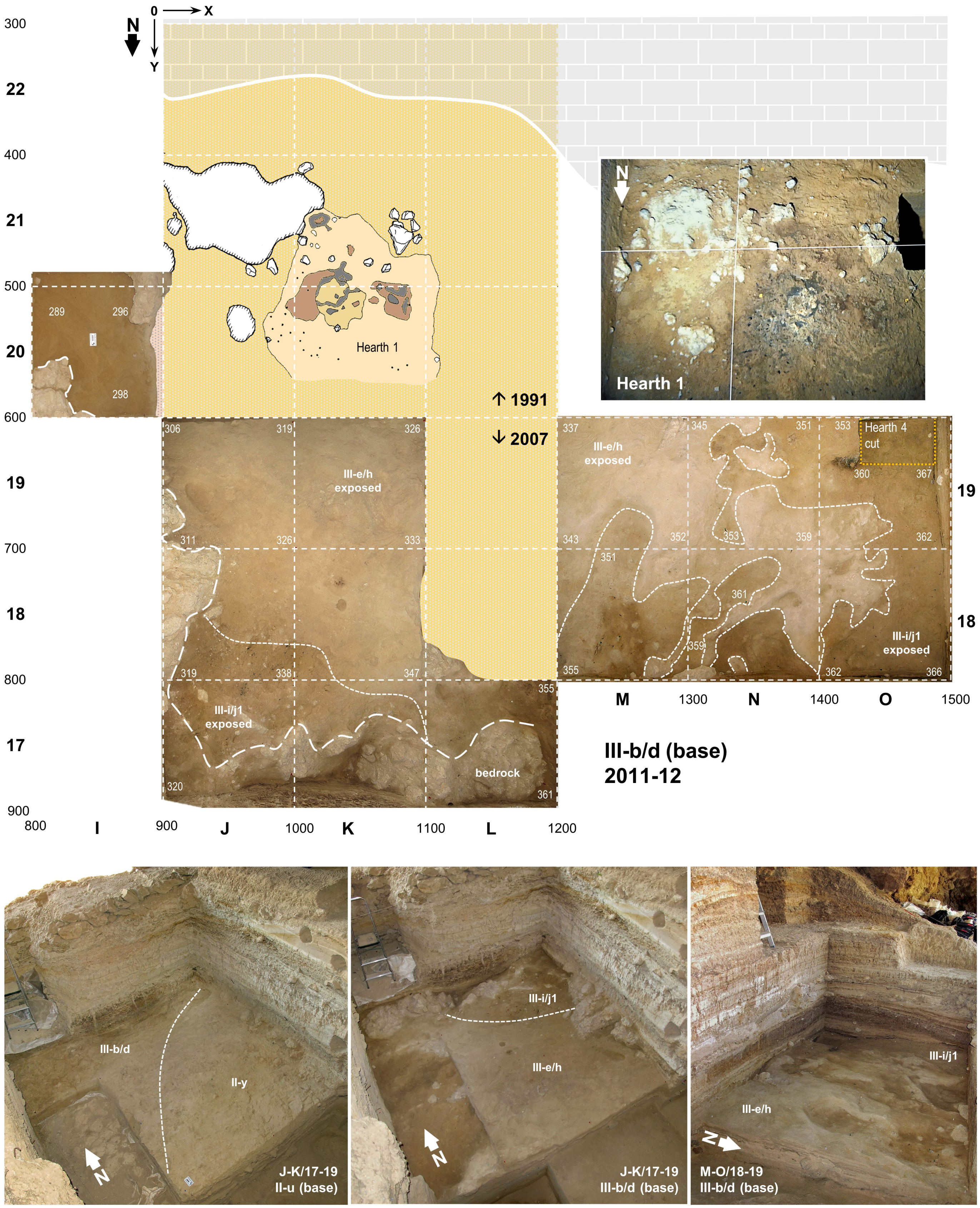

Fig. S5. Excavation of layer III-b/d. Top. Orthorectified view of the 2011-12 décapage with plan of the 1991-excavated Hearth 1 (after Martínez-Sánchez, 1997, modified); the inset is an oblique view of the latter (image courtesy of C. Martínez-Sánchez). **Bottom.** Oblique views of the décapage and excavation of layer III-b/d in J-K/17-19 (2011; left and middle) and in M-N/18-19 (2012; right). The outline of the south wall is given as observed at the top of the sedimentary fill. Elevations are in cm below datum.

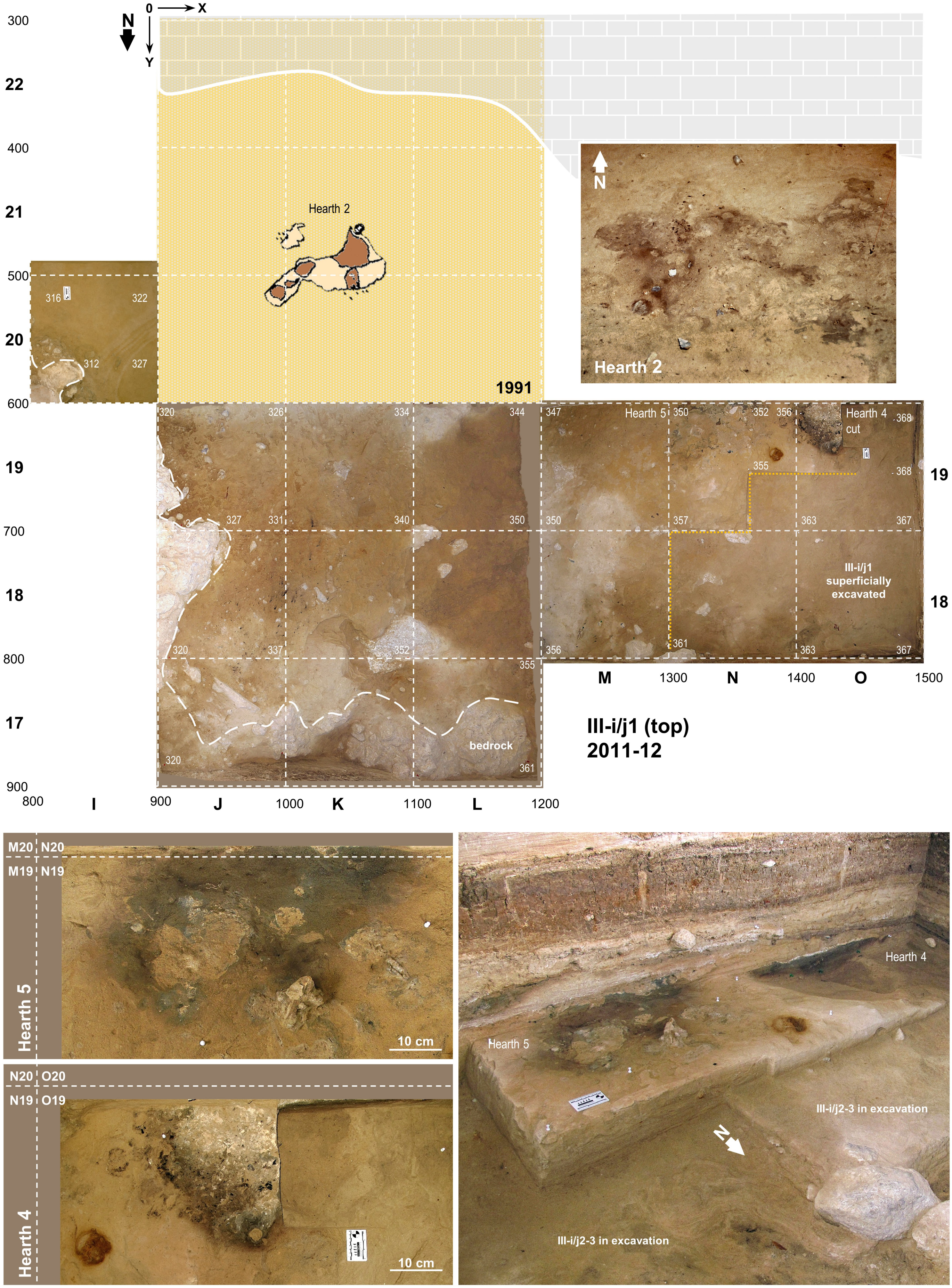

Fig. S6. Excavation of the III-i/j1 lens. Top. Orthorectified view of the 2011-12 décapage with plan of the 1991-excavated Hearth 2 (after Martínez-Sánchez, 1997, modified); the inset is an oblique view of the latter (image courtesy of C. Martínez-Sánchez). **Bottom.** Orthorectified plan (left) and oblique (right) views of the décapage and excavation of Hearths 4 and 5. The outline of the south wall is given as observed at the top of the sedimentary fill. Elevations are in cm below datum.

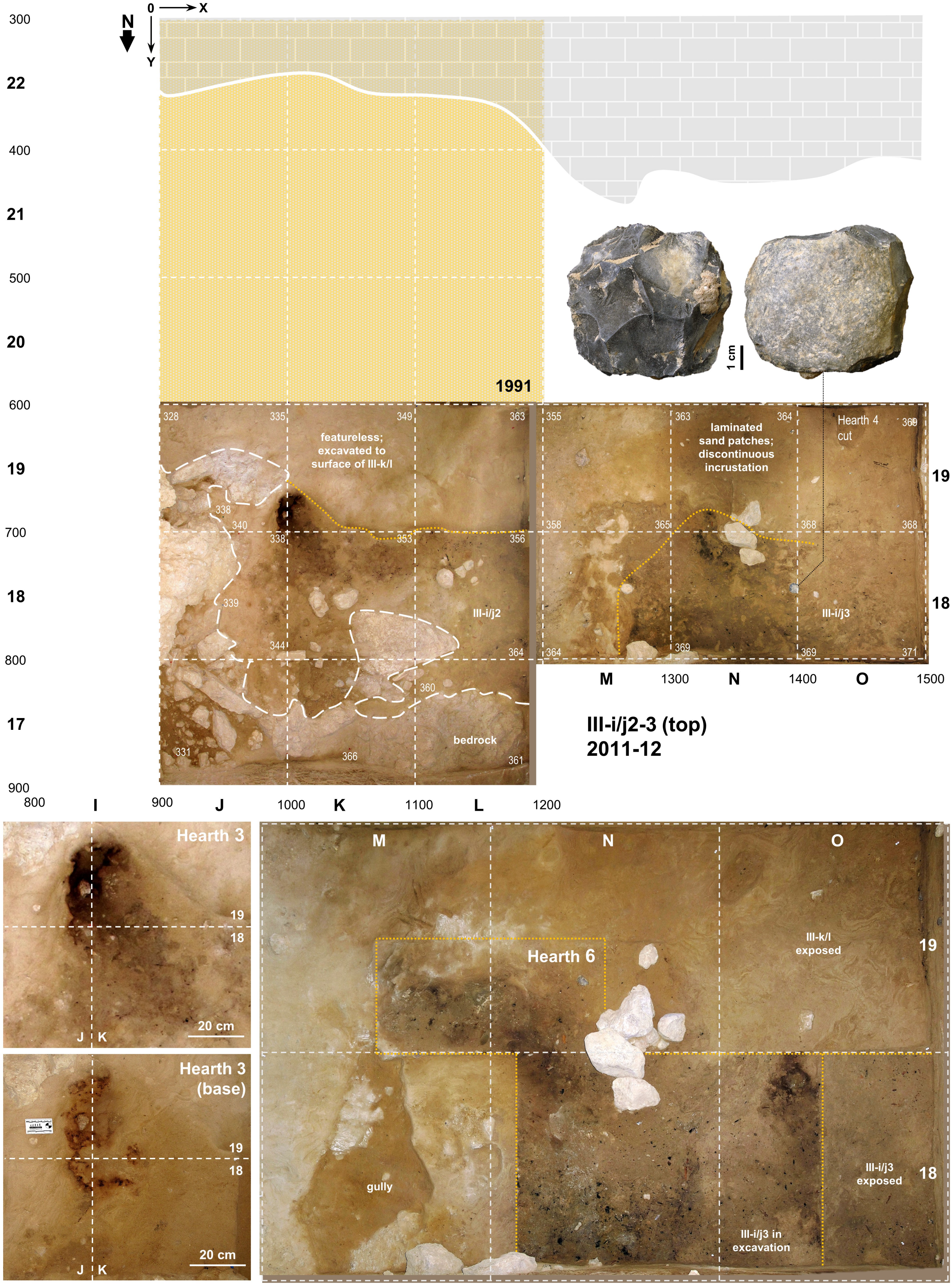

Fig. S7. Excavation of the III-i/j2-3 lens. Top. Orthorectified view of the 2011-12 décapage (the blocks in N/18-19 are roof fall post-dating the occupation; the Levallois limestone core 2012-653 is shown). **Bottom.** Orthorectified, plan views of Hearth 3, as exposed (left, above) and once the ash and charcoal layer was removed to expose the subsurface reddening (left, below), and of the Hearth 6 complex half-way through its excavation (right). The outline of the south wall is given as observed at the top of the sedimentary fill. Elevations are in cm below datum.

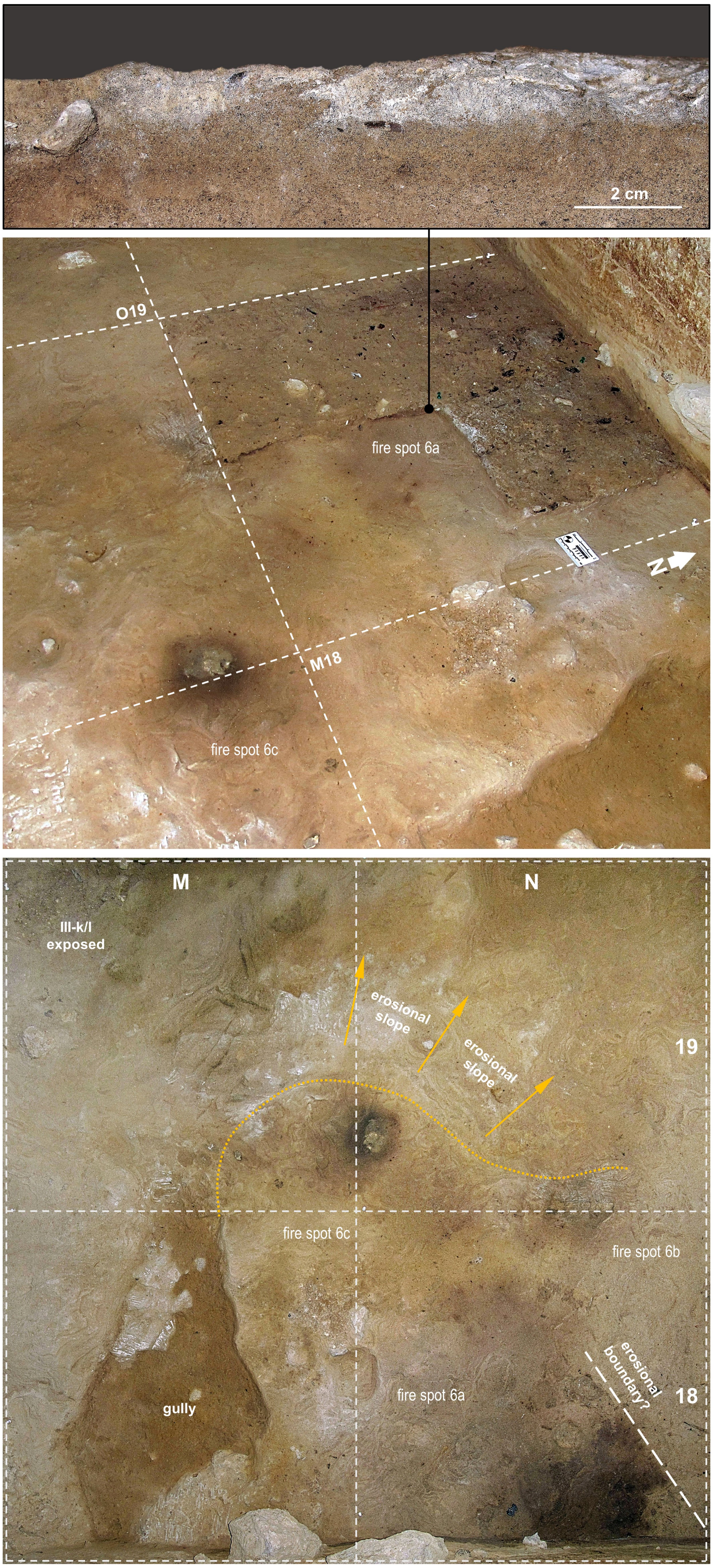

Fig. S8. The Hearth 6 complex. Top. Field-macrophotography detailing the ash-over-burnt-sands microstratigraphy above fire spot 6a. **Middle**. Oblique view illustrating the stratigraphic relationship between the top and bottom of the ash and charcoal concentration defined as "Hearth 6" (note the subsurface reddening apparent once the thin sheet of blackened sediment had been excavated). **Bottom.** Orthorectified view of the base of the Hearth 6 complex, showing the three distinct subsurface fire spots designated 6a, 6b and 6c.

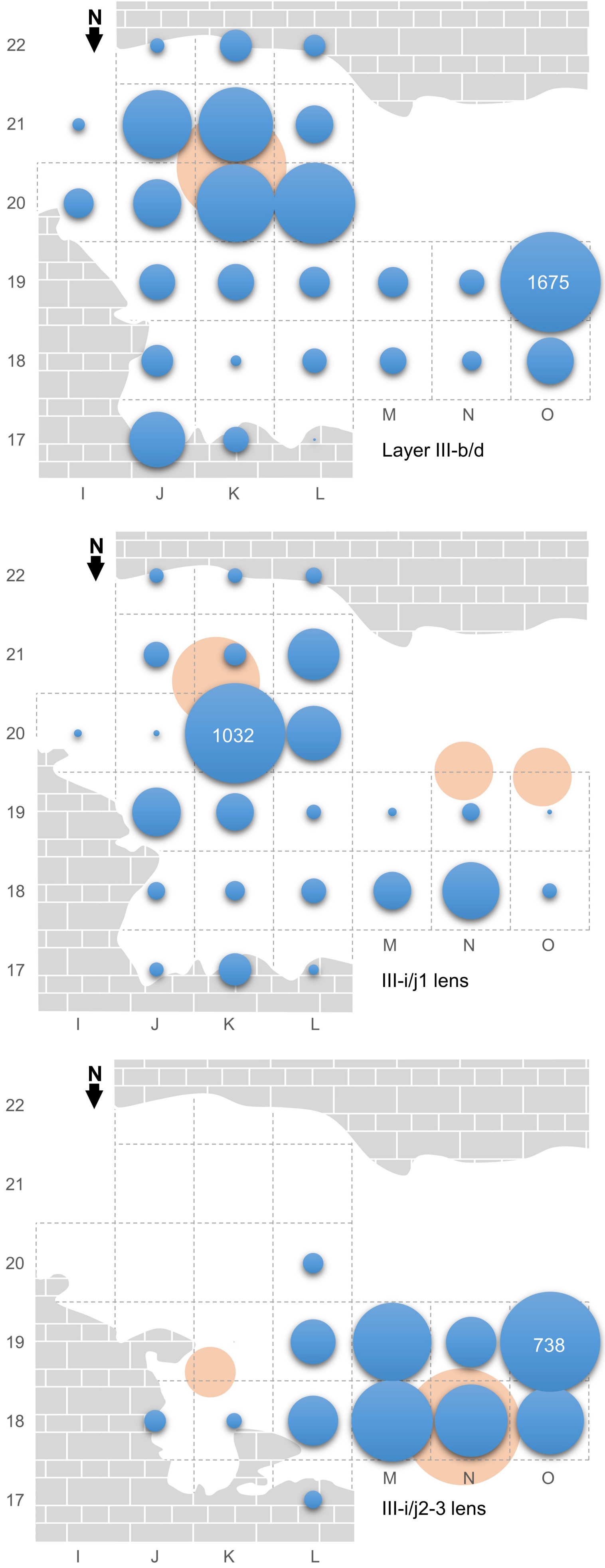

Fig. S9. Spatial distribution of knapped stone tools, by one square meter grid unit and by weight (g). The size of the blue bubbles is proportional to the grid unit's percentage of the total weight of each layer or lens. The highest value is given, and the position of the combustion features is schematically indicated by the orange circles. The outline of the south wall is given as observed at the top of the sedimentary fill. The outline of the north wall is given as observed at the elevation of each stratigraphic unit.

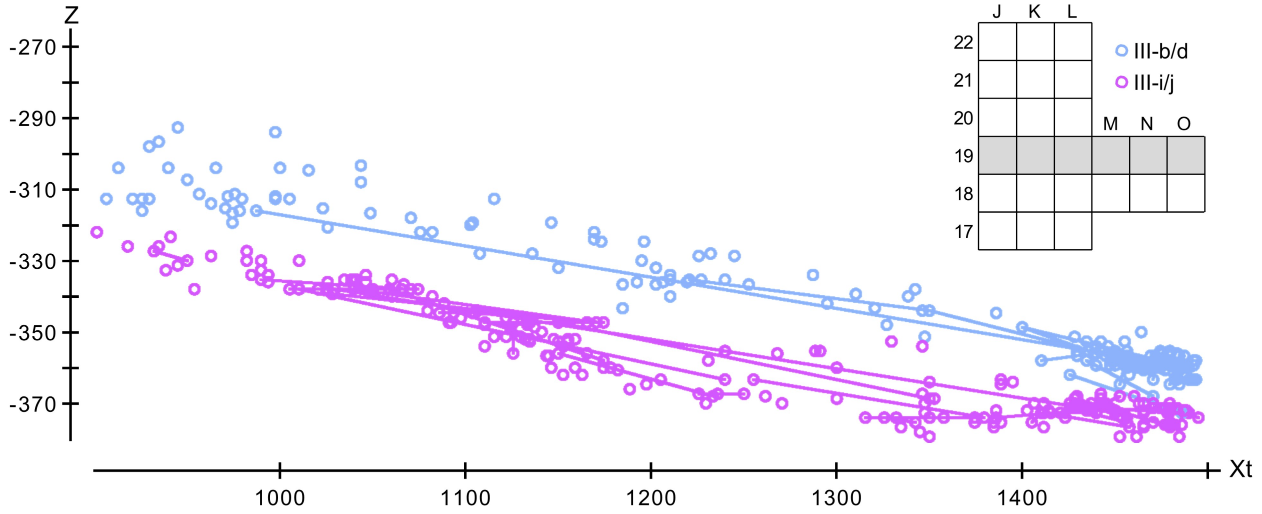

Fig. S10. Vertical distribution of the lithic assemblages from layers III-b/d and III-i/j along row 19 of the grid. The two layers are in direct contact in column O only; otherwise, they are separated by a sterile inundation unit (layer III-e/h) that wedges out in column N. The refit links show that no post-depositional mixing occurred and that the lithic assemblages are stratigraphically intact. The scatter graph features all piece-plotted ltihics (for the purposes of this representation, the sieve items in refit units were also included; they were assigned the [x,z] coordinates of the center of the square meter or quarter-square meter of provenience, with elevation set at the mid-point of the stratigraphic unit's thickness).

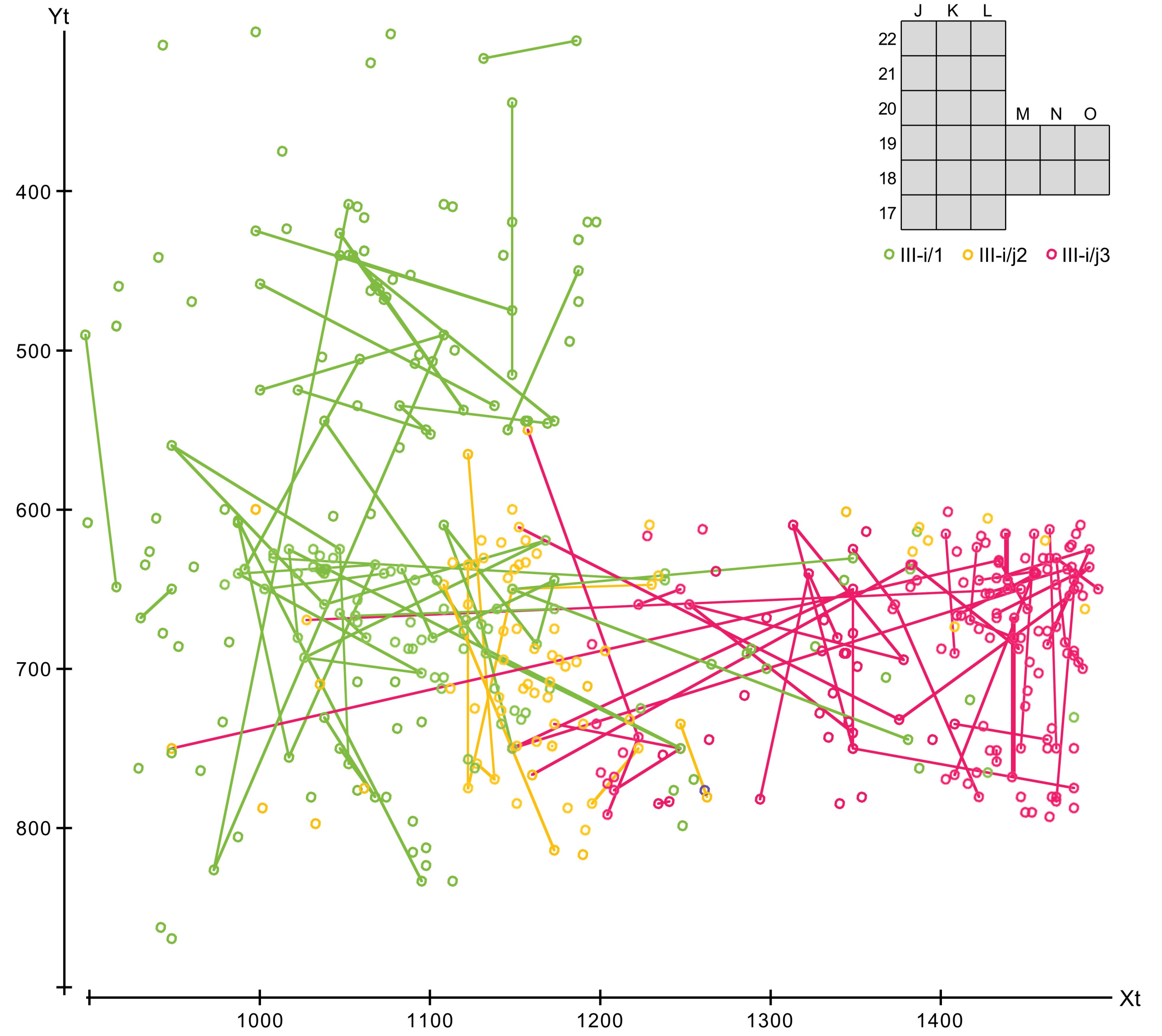

Fig. S11. Horizontal distribution of the lithic assemblages from layer III-i/j. During the 2011-12 field seasons, this layer was found to be stratigraphically structured. Two lenses were recognized, III-i/j1 and III-i/j2-3; the basal lens was further subdivided into two horizontal clusters (III-i/j2 and III-i/j3) that, though at the same elevation, were separated by an empty area. The 1991 finds were assigned to i/j1, except in three cases found to refit with material from i/j2-3. The scatter graph features all piece-plotted ltihics (for the purposes of this representation, the sieve items included in refit units were assigned the [x,y] coordinate of the center of the square meter or quarter-square meter of provenience).

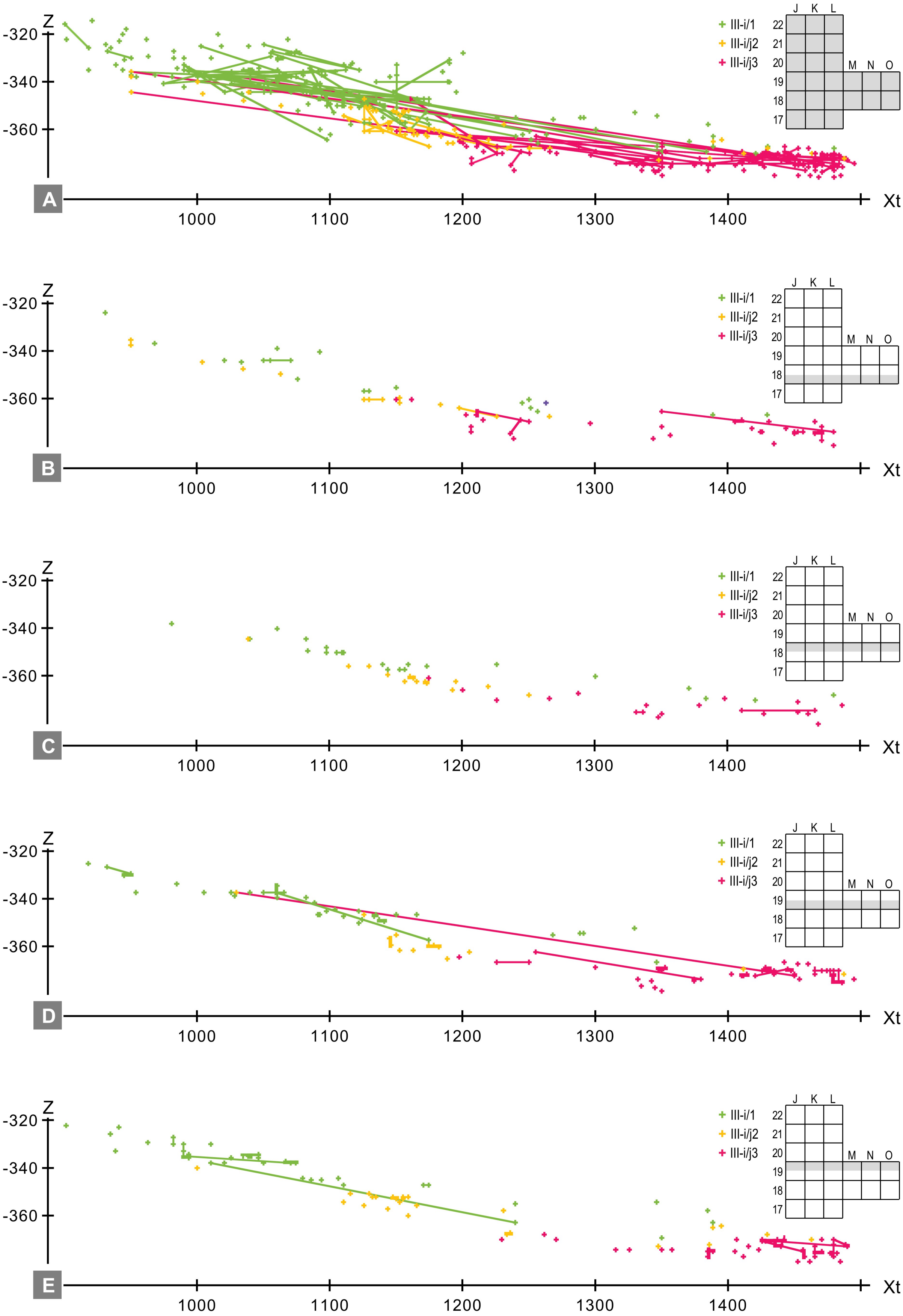

Fig. S12. Vertical distribution along the E-W axis of the grid of the lithic assemblages from layer III-i/j. A. Entire excavated area of the layer. **B.** North half of row 18 only. **C.** South half of row 18 only. **D.** North half of row 19 only. **E.** South half of row 19 only. The scatter graphs feature all piece-plotted ltihics (for the purposes of these representations, the sieve items in refit units were also included; they were assigned the [x,z] coordinates of the center of the square meter or quarter-square meter of provenience, with elevation set at the mid-point of the stratigraphic unit's thickness).

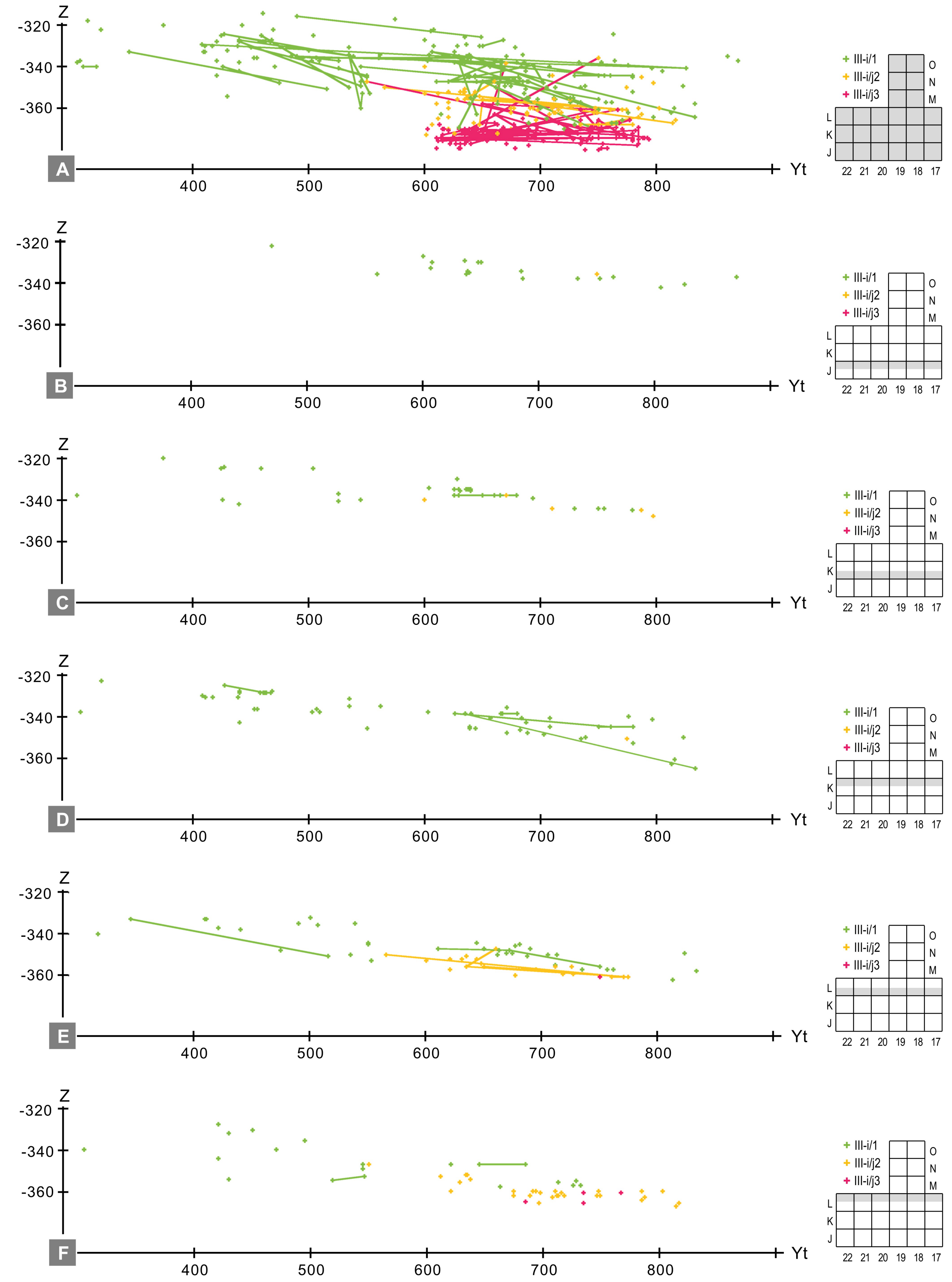

Fig. S13. Vertical distribution along the N-S axis of the grid of the lithic assemblages from layer III-i/j. A. Entire excavated area of the layer. **B.** West half of column J only. **C.** East half of column K only. **D.** West half of column K only. **E.** East half of column L only. **F.** West half of column L only. The scatter graphs feature all piece-plotted ltihics (for the purposes of these representations, the sieve items in refit units were also included; they were assigned the [y,z] coordinates of the center of the square meter or quarter-square meter of provenience, with elevation set at the mid-point of the stratigraphic unit's thickness).

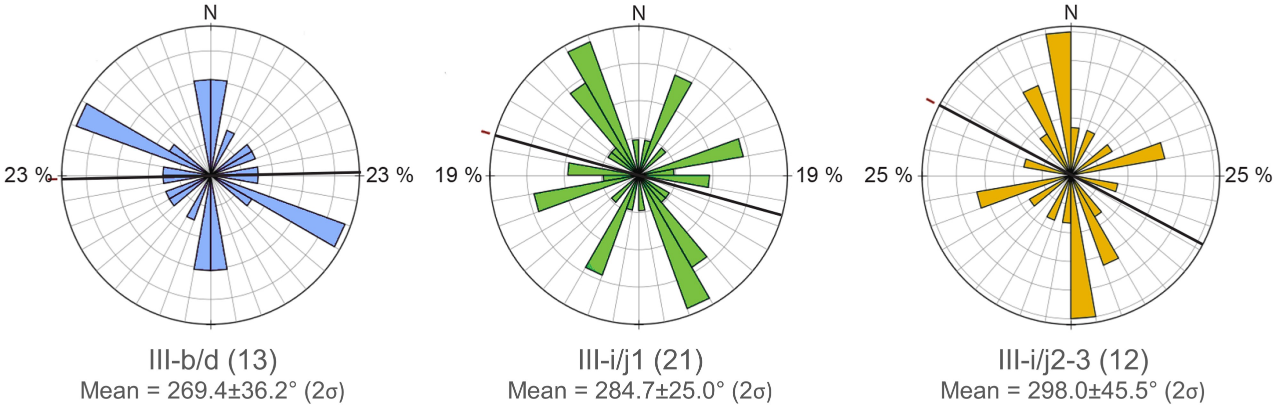

	III-b/d		III-i/j1		III-i/j2-3	
	test statistic	p-value	test statistic	p-value	test statistic	p-value
Rayleigh	0.6007	0.0068	0.6359	0.0001	0.5415	0.0261
Kuiper	1.9631	0.0100<0.025	17.4104	<0.0100	1.8519	0.0250<0.0500
Watson	0.2634	0.0100<0.0250	21.8229	<0.0100	0.2223	0.0100<0.0250

Fig. S14. Rose diagrams and statistics for refit orientations in layer III-b/d and the III-i/j1 and III-i/j2-3 lenses. For each stratigraphic unit, the number of refit units is indicated in parenthesis; only piece-plotted items were considered.

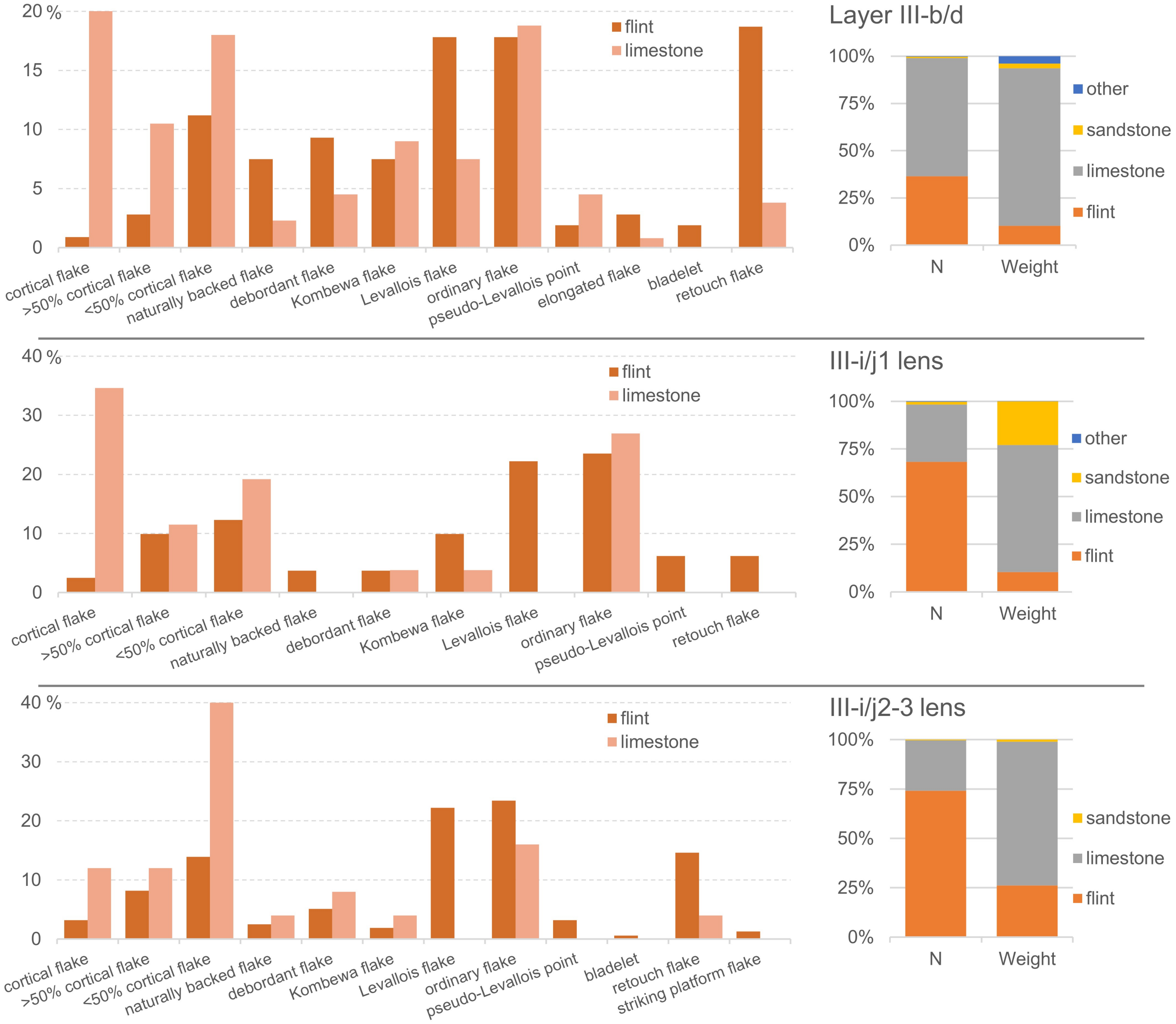

Fig. S15. Technology and raw materials. Relative frequency per stratigraphic unit. For the technology graphs, small flakes (those <2 cm in maximum dimension) and flake fragments were excluded from consideration unless they could be identified to a specific category.

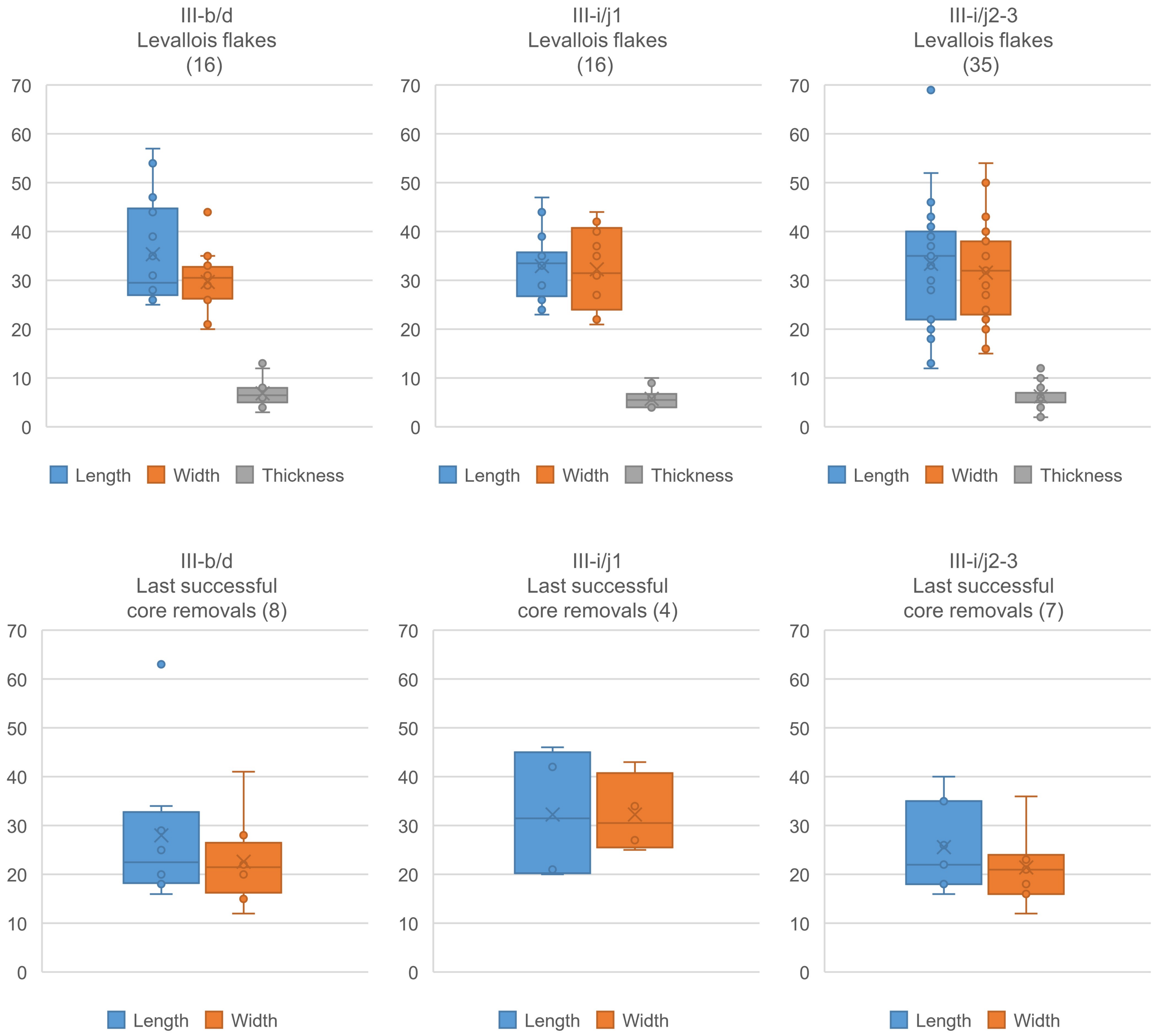

Fig. S16. Size of chert blanks. In the boxplots for each stratigraphic unit, the number of measured items is indicated in parenthesis.

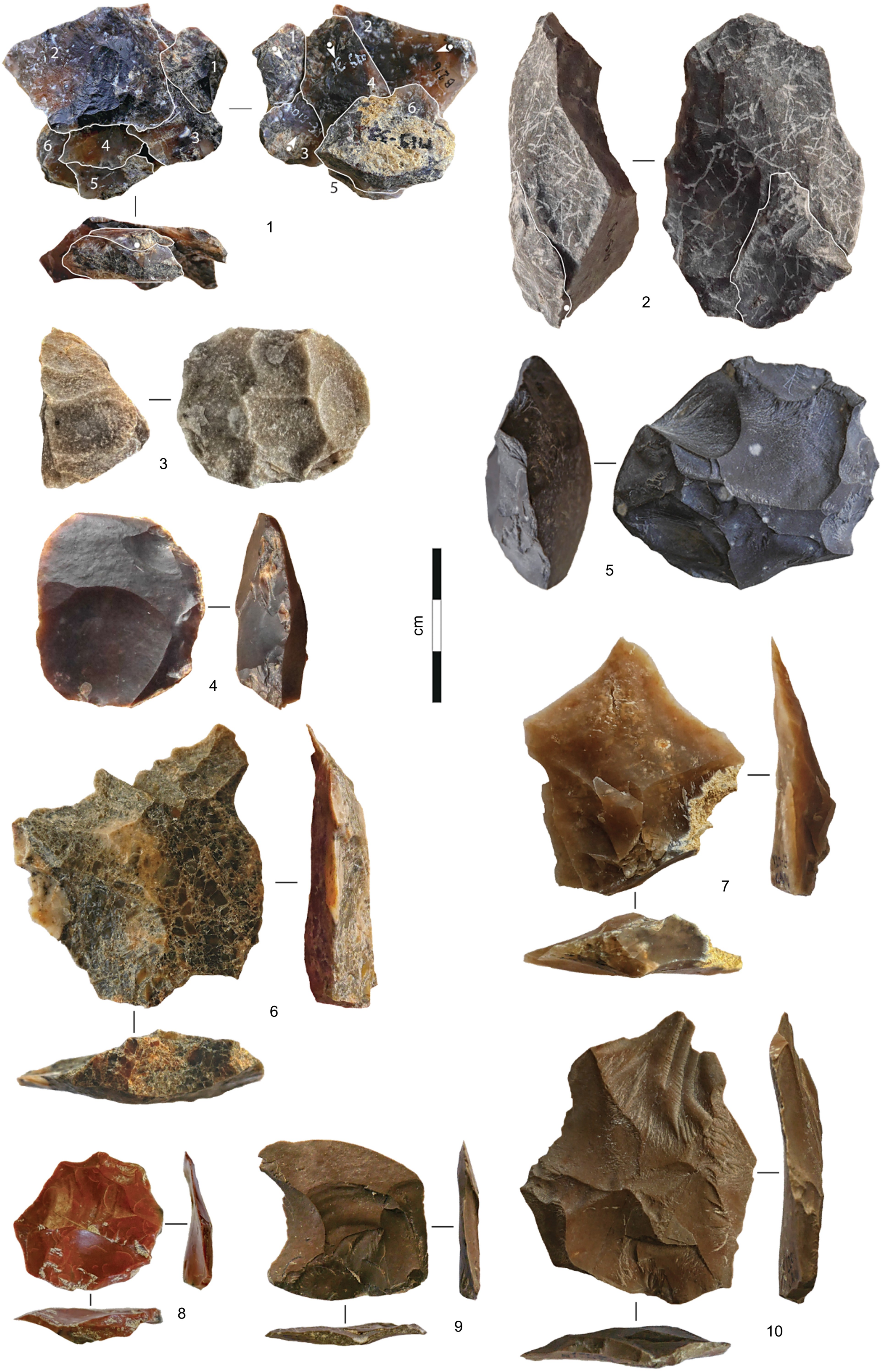

Fig. S17. Layer III-b/d: stone tools. 1. Refit unit (chert). **2.** Refit unit (limestone). **3-4.** Cores (chert). **5. Core (**limestone). **6-8.** Flakes (chert). **9-10.** Levallois flakes (limestone).

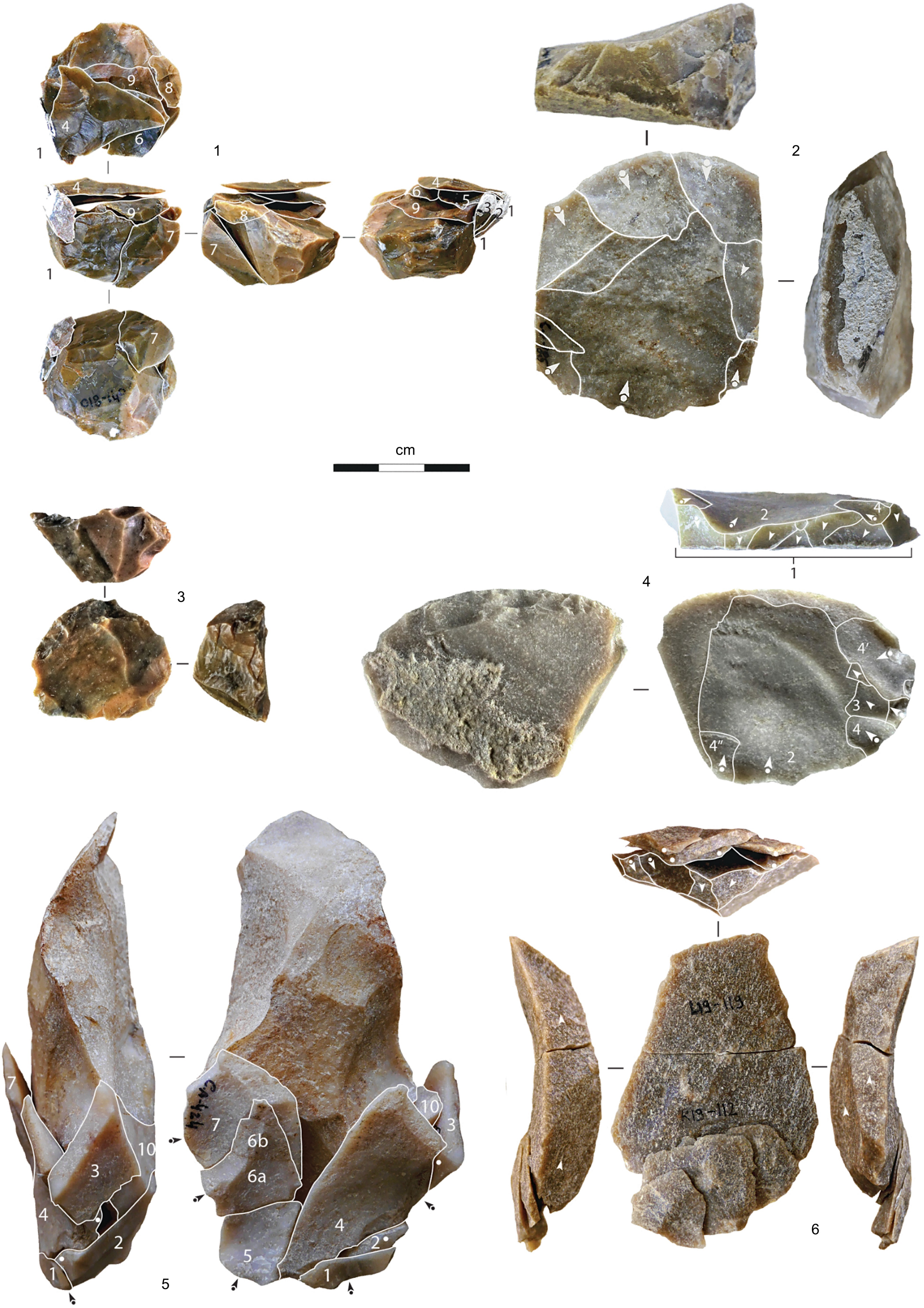

Fig. S18. Layer III-i/j: stone tools (chert). 1. Refit unit #45 (III-i/j2-3); **2.** Levallois core (III-i/j1); **3.** Levallois core (III-i/j2-3) **4.** Kombewa core on a sidescraper blank (III-i/j2-3). **5.** Refit unit #50 (III-i/j1); **6.** Refit unit #59 (III-i/j1).

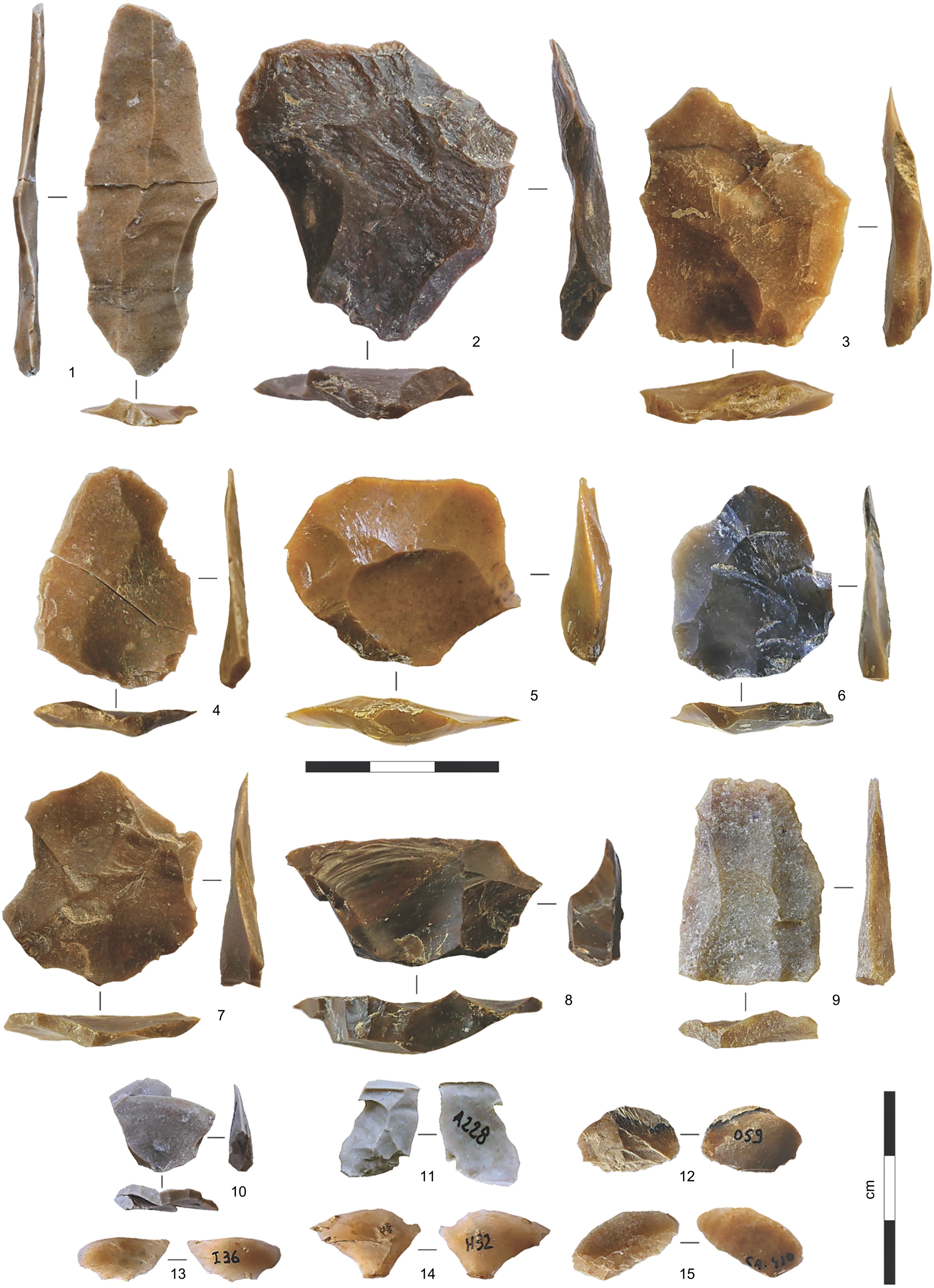

Fig. S19. Layer III-i/j: stone tools (chert). 1-9. Levallois flakes; **10.** Refit unit #56 (two Levallois micro-flakes). **11-15.** Retouch flakes. Numbers 2, 4, 9, 12 and 15 are from the III-i/j1 lens; numbers 1, 3, 5-8, 10-11, and 13-14 are from the III-i/j2-3 lens.

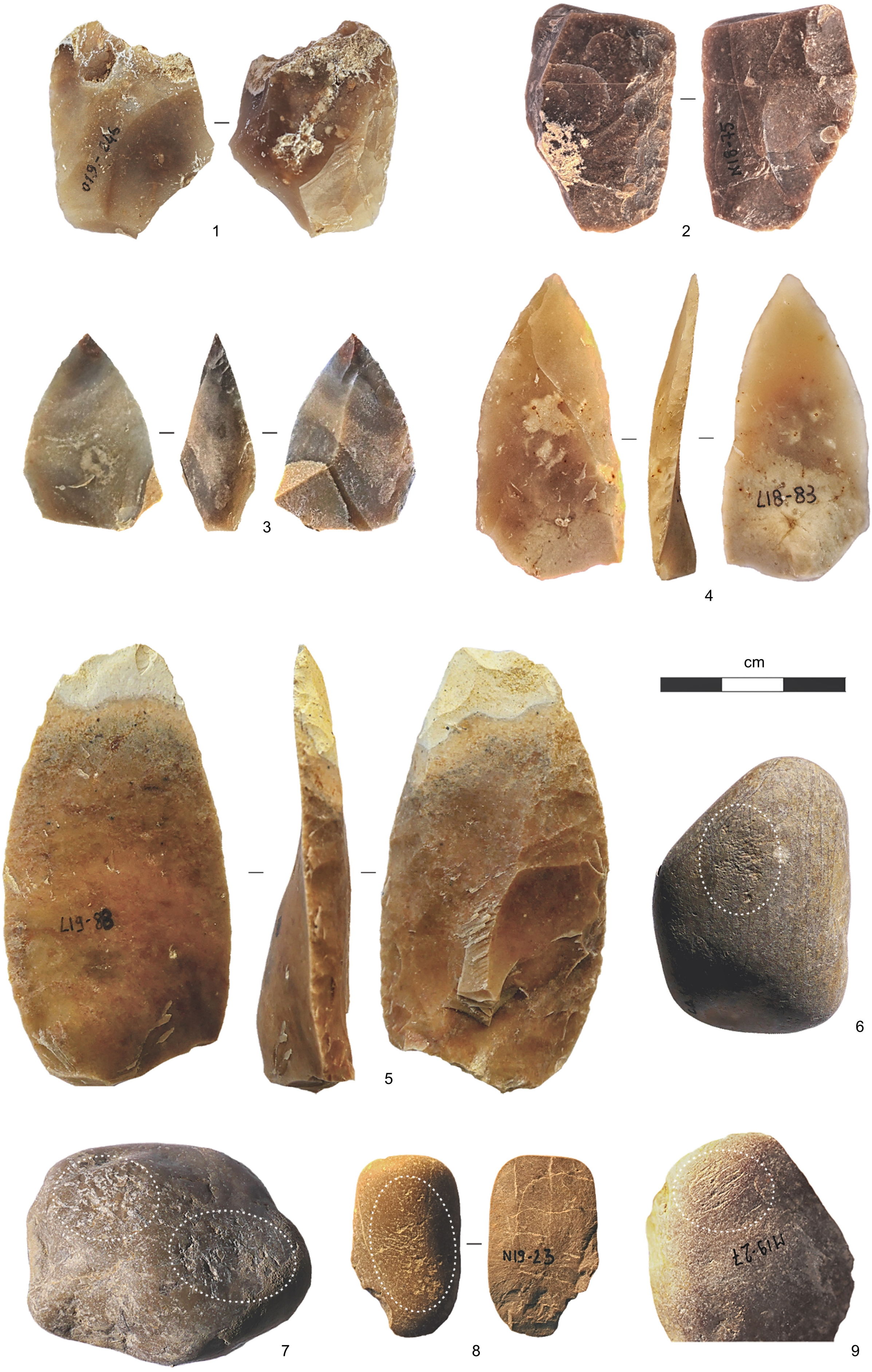

Fig. S20. Retouchers and retouched tools. 1-2. Kombewa-exploited sidescrapers (III-i/j3; chert). **3-4.** Bilaterally pointed blanks (III-i/j1 and III-i/j2, respectively; chert). **5.** Double sidescraper (III-i/j2; chert). **6-9.** Retouchers on small limestone cobbles (6. III-i/j1; 7. III-i/j3; 8-9. III-b/d).

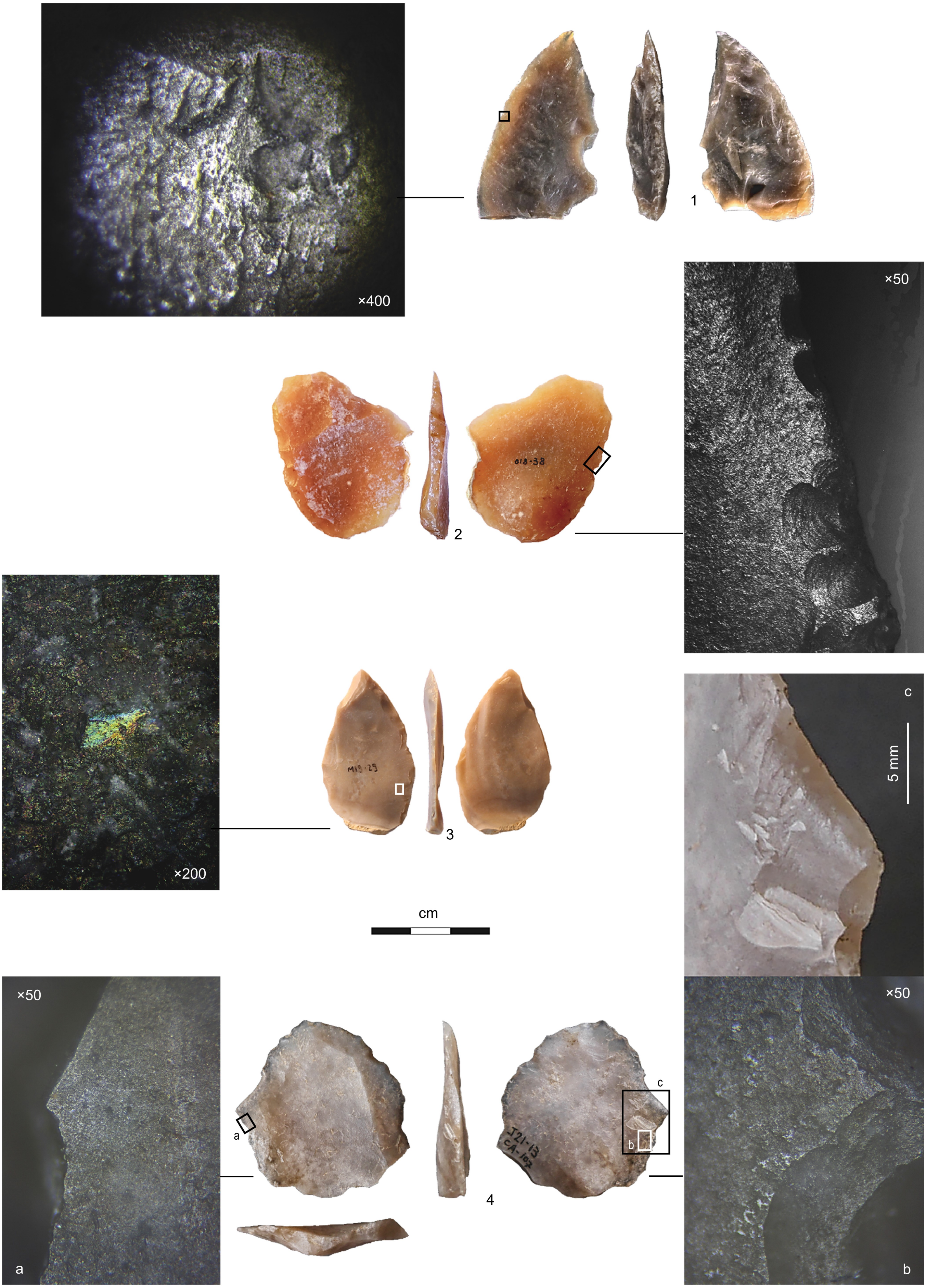

Fig. S21. Tools with on-bone use-wear. 1. III-i/j3 sidescraper (O19-173; chert). **2-3.** III-b/d sidescrapers (O18-38 and M19-29, respectively; chert). **4.** III-i/j1 Levallois flake (J21-13; chert); note the large flexure break (c) intersecting the use-wear evidence (a-b). The rectangles indicate the areas where the illustrated use-wear evidence was recorded.

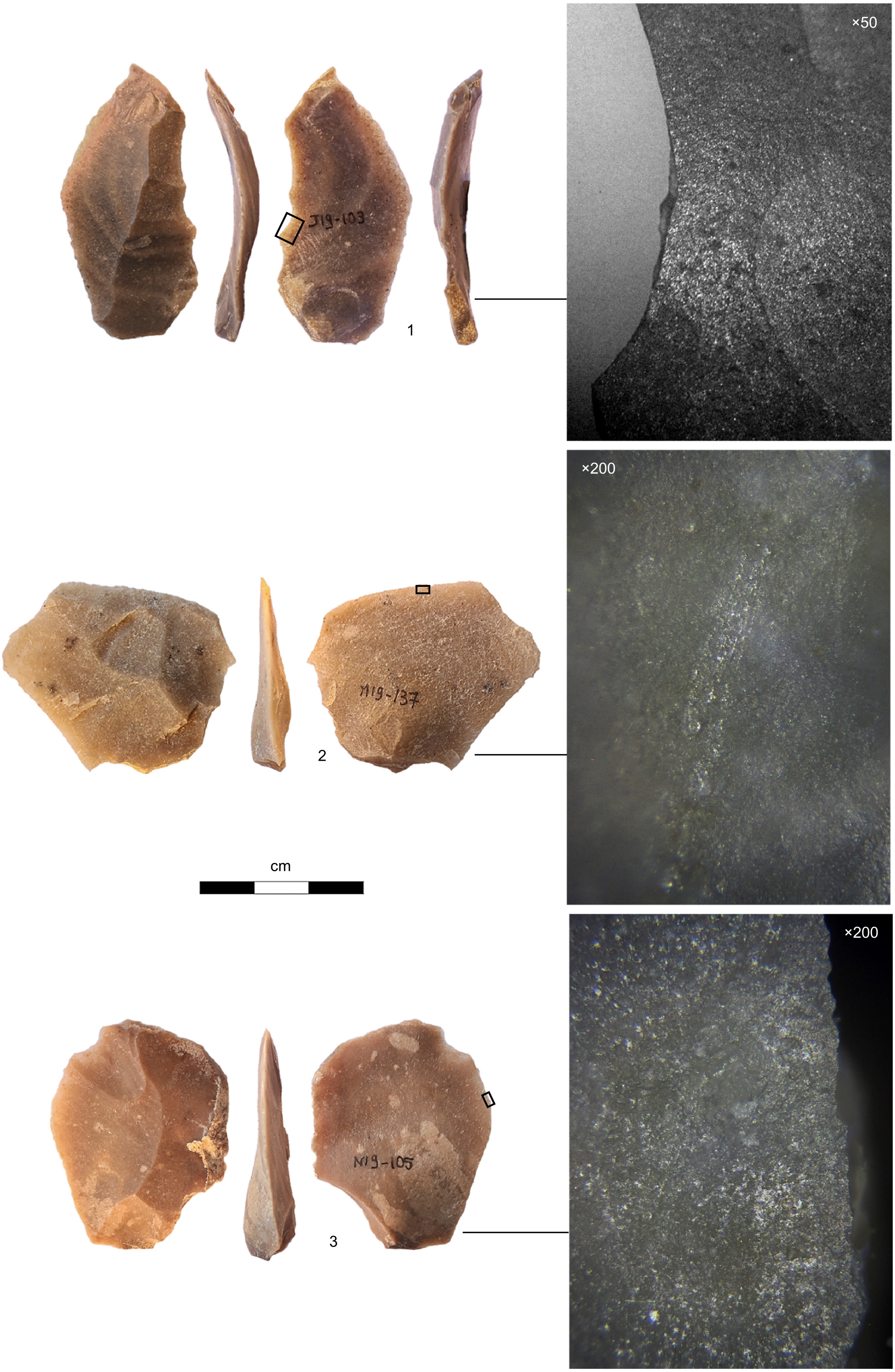

Fig. S22. Flakes with meat-cutting use-wear. 1. III-i/j1 (J19-103; elongated Kombewa blank, chert). **2.** III-i/j3 (M19-137; Levallois blank, chert). **3.** III-i/j3 (N19-105; Levallois blank, chert). The rectangles indicate the areas where the illustrated use-wear evidence was recorded.

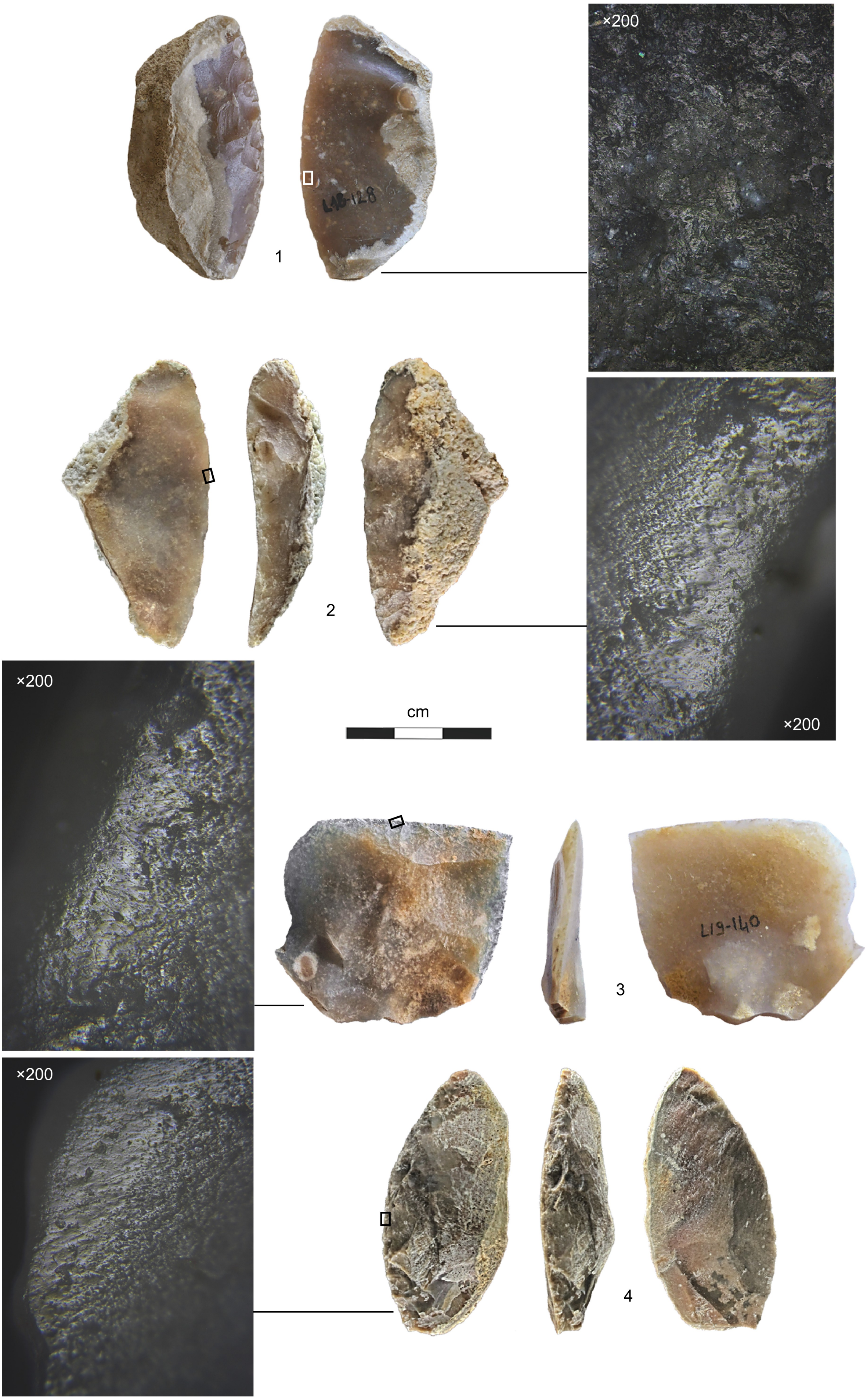

Fig. S23. Sidescrapers with hide (1.) and hide-with-abrasive use-wear (2-4.) 1. III-i/j2 (L18-128). **2.** III-i/j1 (K19-120). **3.** III-i/j2 (L19-140). **4.** III-b/d (N18-12). The rectangles indicate the areas where the illustrated use-wear evidence was recorded.

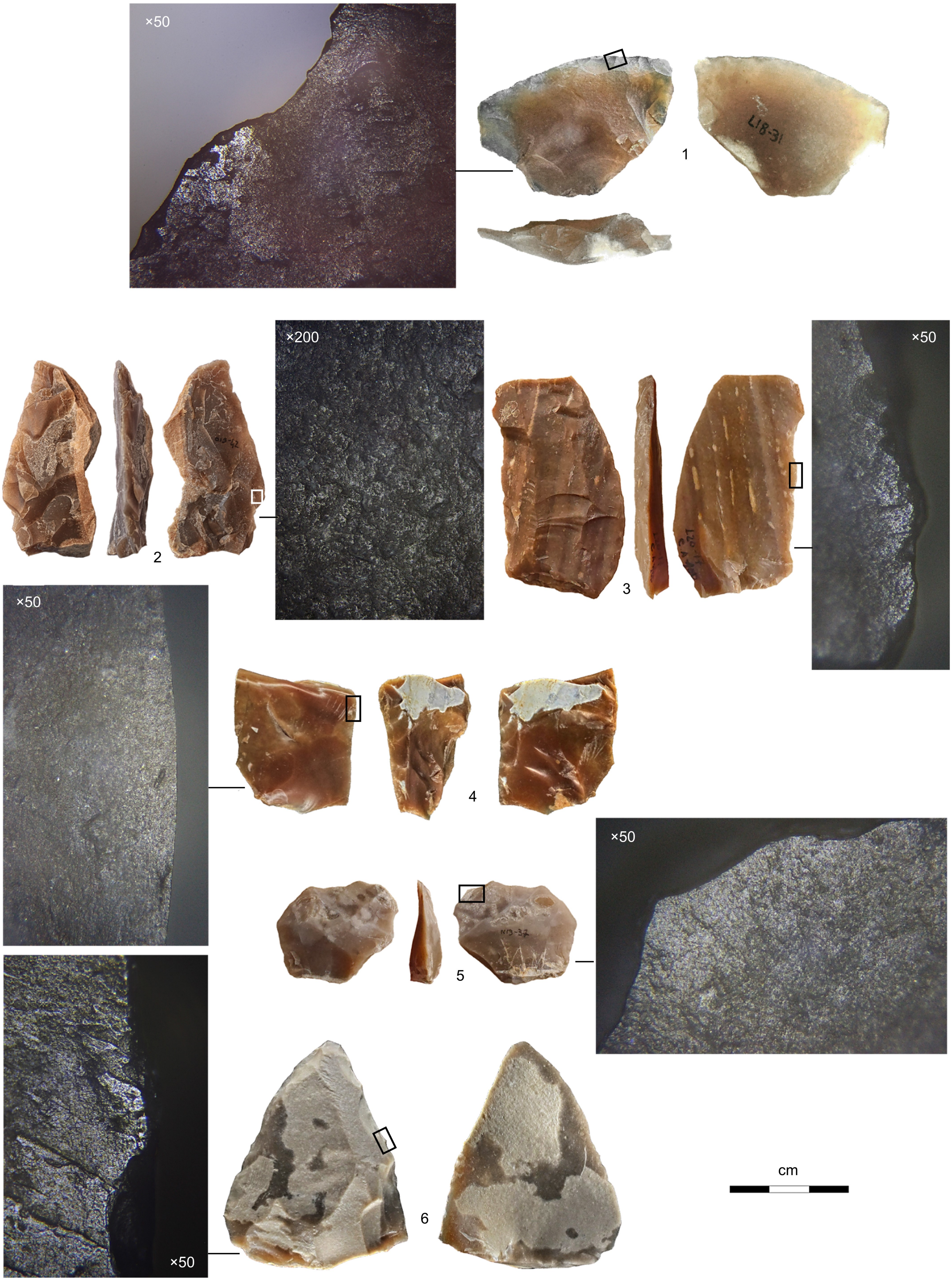

Fig. S24. Tools with on-wood use-wear. 1. III-i/j1 (L18-31, chert)**. 2-5.** III-b/d (O19-42, L20-17, N19-15 and N19-37, respectively; chert). **6.** III-i/j3 (O19-189; chert). The rectangles indicate the areas where the illustrated use-wear evidence was recorded.

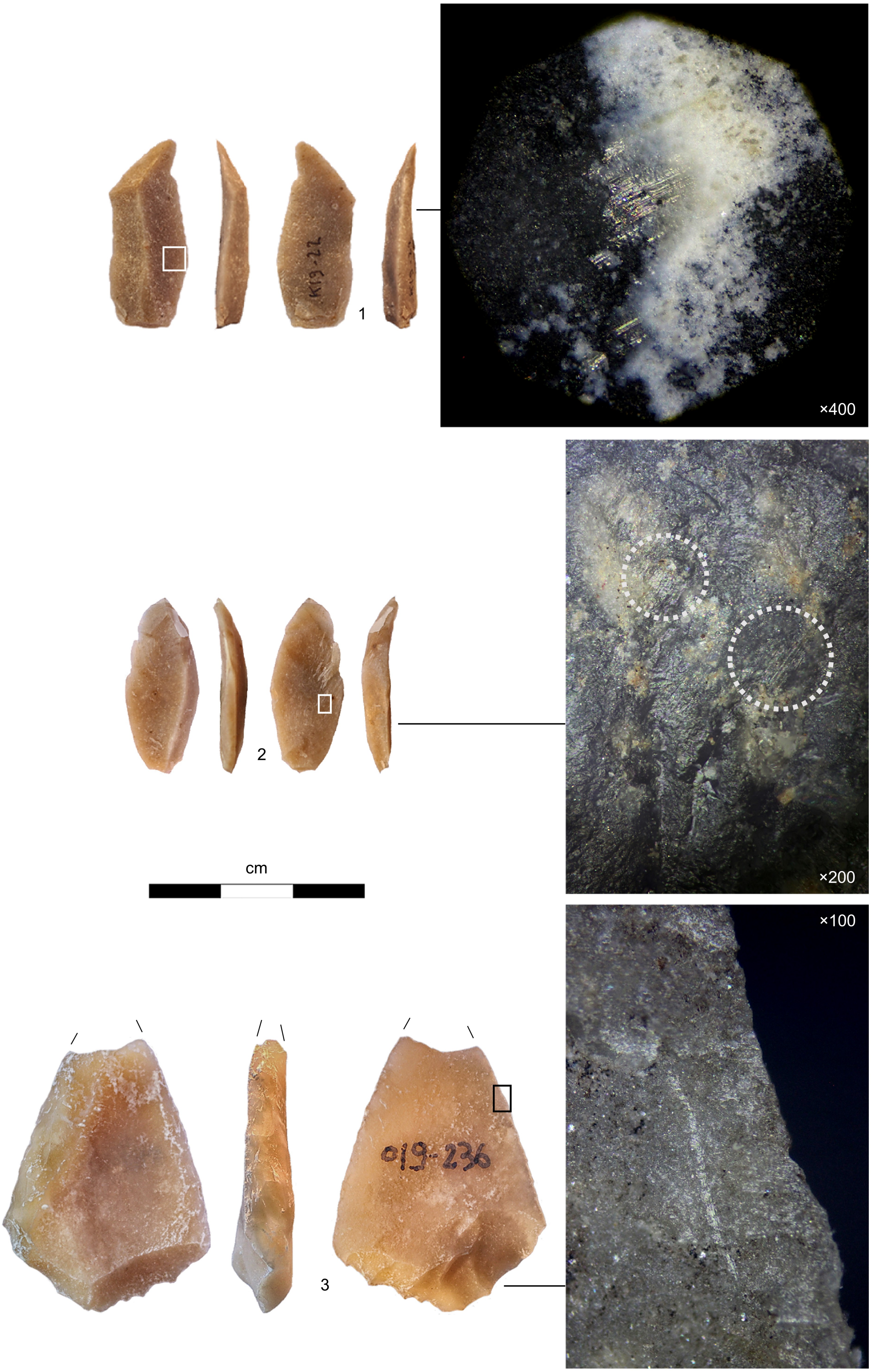

Fig. S25. Tools with projectile use-wear. 1. Small elongated flake (K19-22) from III-b/d with hafting wear. **2-3.** Small elongated flake (O19NE; B-451) and Mousterian point (O19-236) from III-i/j3 with ventral thinning of the base and impact striations. The rectangles indicate the areas where the illustrated use-wear evidence was recorded.

Pairwise comparisons using permutation MANOVA

	Bone	Hide	Hide/abrasive	Meat
Hide	0.391	–	–	–
Hide/abrasive	0.005	0.179	–	–
Meat	0.014	0.011	0.001	–
Wood	0.802	0.241	0.002	0.004

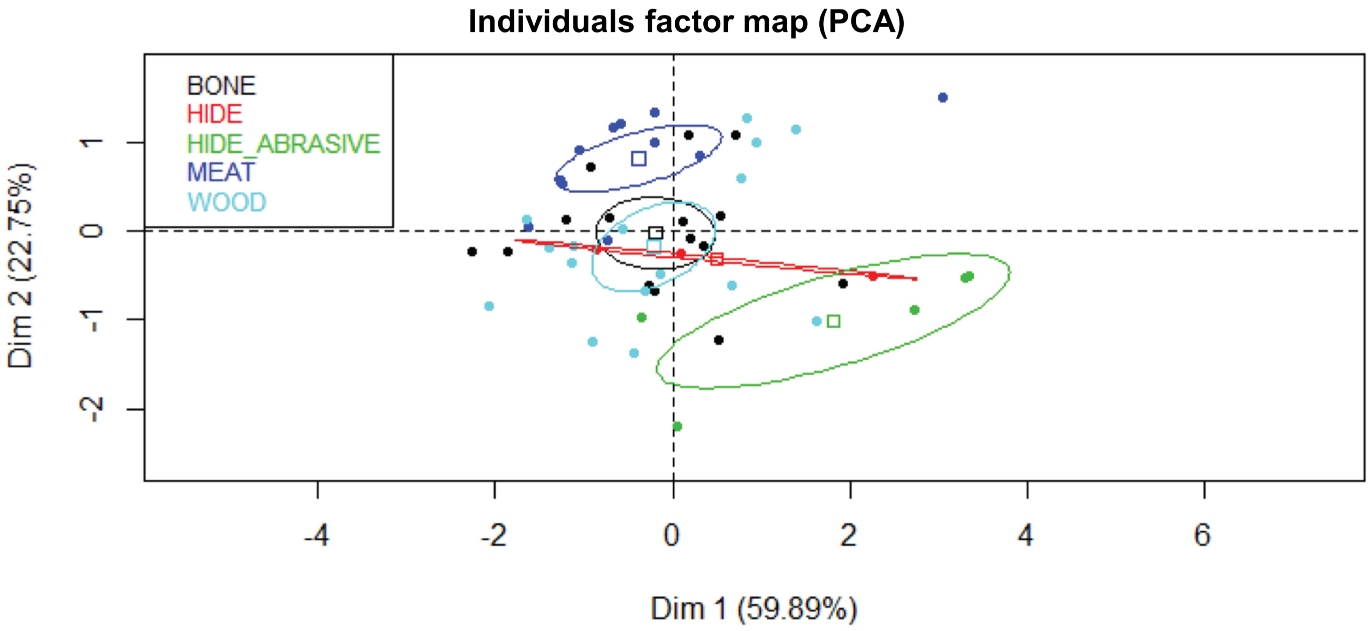

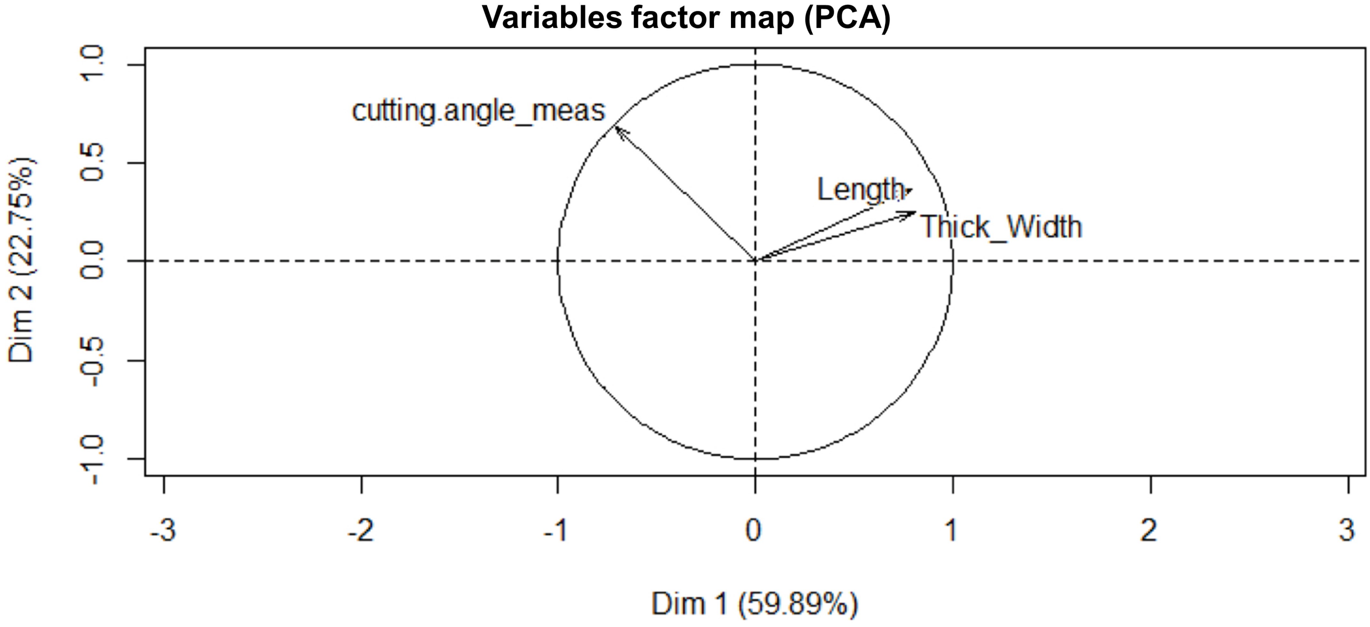

Fig. S26. Relationship between blank metrics and use-wear type: results of Principal Components Analysis. Top. Use wear PCA graph (the ellipses represent confidence levels generated during the PCA analysis using a standard 0.95 value) and associated MANOVA p-values. **Bottom**: factor map for visual interpretation of the PCA graph, illustrating the data points' distribution tendencies based on the contribution of the metric variables (Cutting Angle, Length, and Width/Thickness ratio) to the canonical variables Dim 1 and Dim 2; based on the slope of the lines, Cutting Angle is more relevant for Dim 2, while Length and Width/Thickness ratio are more relevant for Dim 1.

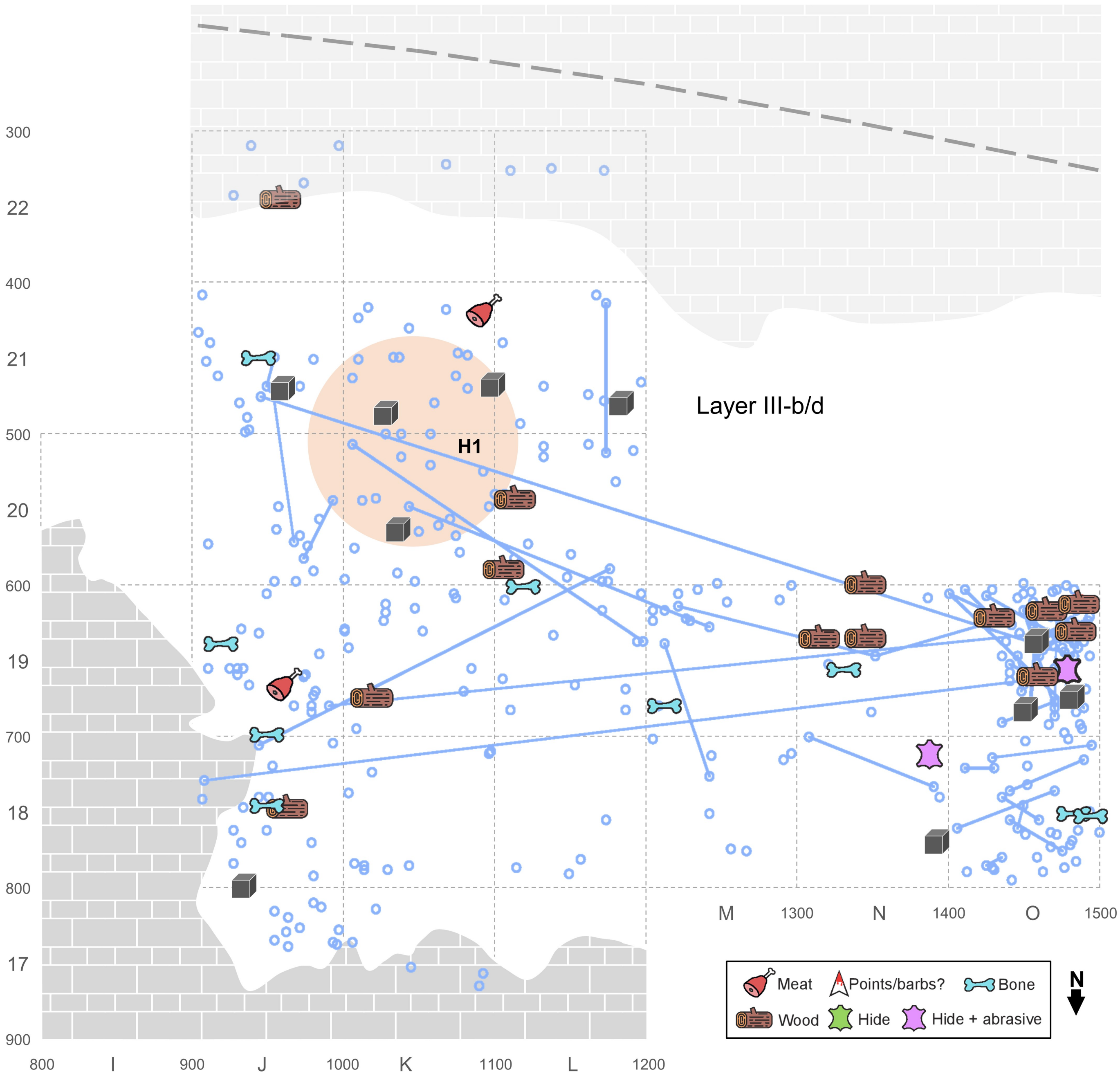

Fig. S27. Layer III-b/d: spatial structure and activities. The grey cubes represent piece-plotted cores-on-block, the circles represent other piece-plotted lithics, and the lines represent refit links (for the purposes of this representation, the sieve items included in refit units were assigned the [x,y] coordinate of the center of the square meter or quarter-square meter of provenience). The position of Hearth 1 (H1) is schematically indicated. The symbols coded by material type or function denote the position of stone tools bearing the corresponding, diagnostic use-wear. The outline of the bedrock shelf bounding the infill to the North is given as observed at the elevation of the layer's surface. The outline of the back wall is given as observed at the top of the sedimentary infill, prior to excavation, and, approximately (long-dash line), at the layer's surface, as inferred from the concave configuration displayed in the J>I, L>M (Fig. S2), and N>M stratigraphic profiles.

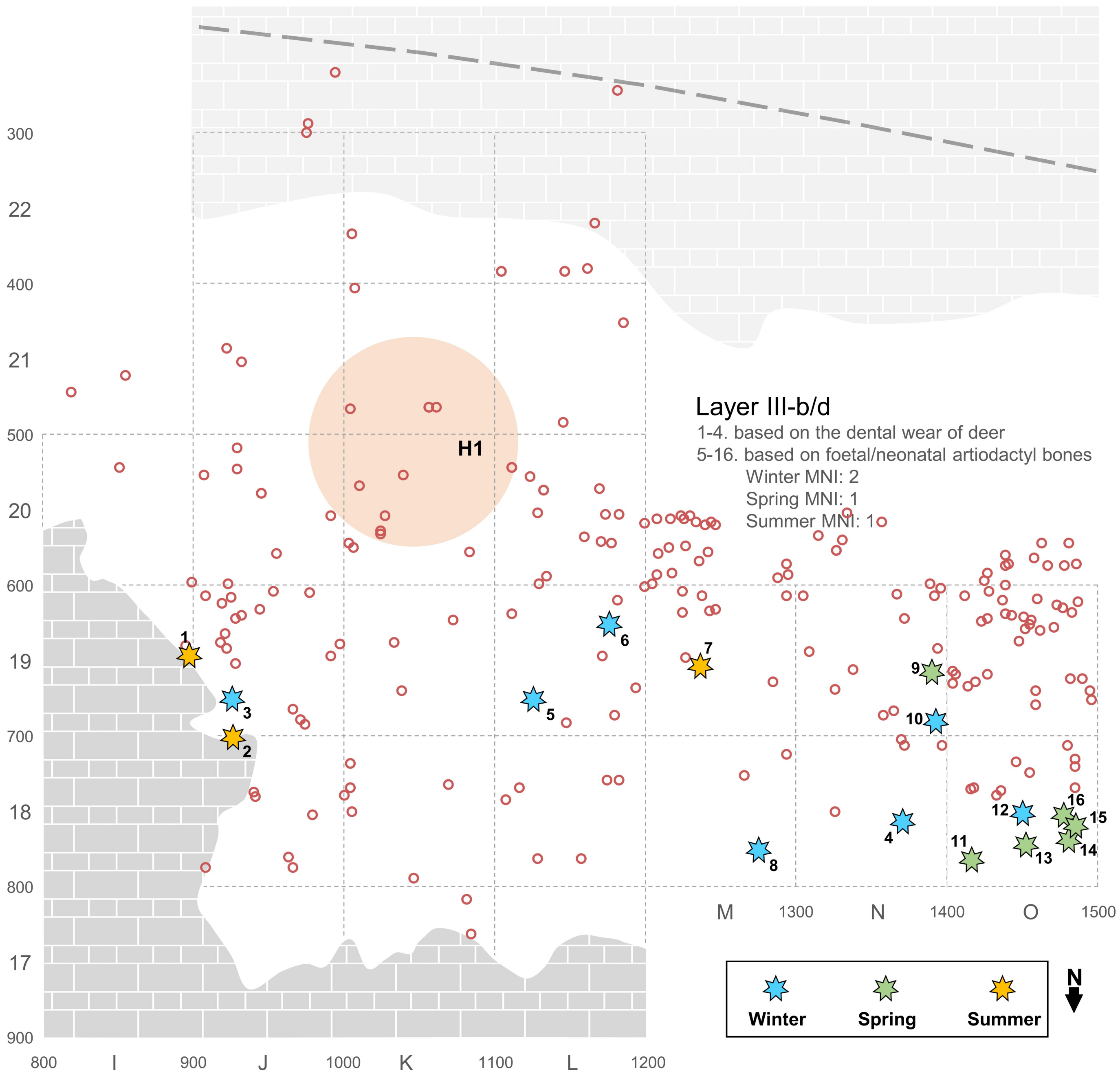

Fig. S28. Layer III-b/d: spatial structure and seasonality. The points represent piece-plotted faunal remains (the sieve items with seasonality information were assigned the [x,y] coordinate of the center of the square meter or quarter-square meter of provenience). The position of Hearth 1 (H1) is schematically indicated. The outline of the bedrock shelf bounding the infill to the North is given as observed at the elevation of the layer's surface. The outline of the back wall is given as observed at the top of the sedimentary infill, prior to excavation, and, approximately (long-dash line), at the layer's surface, as inferred from the concave configuration displayed in the J>I, L>M (Fig. S2), and N>M stratigraphic profiles.

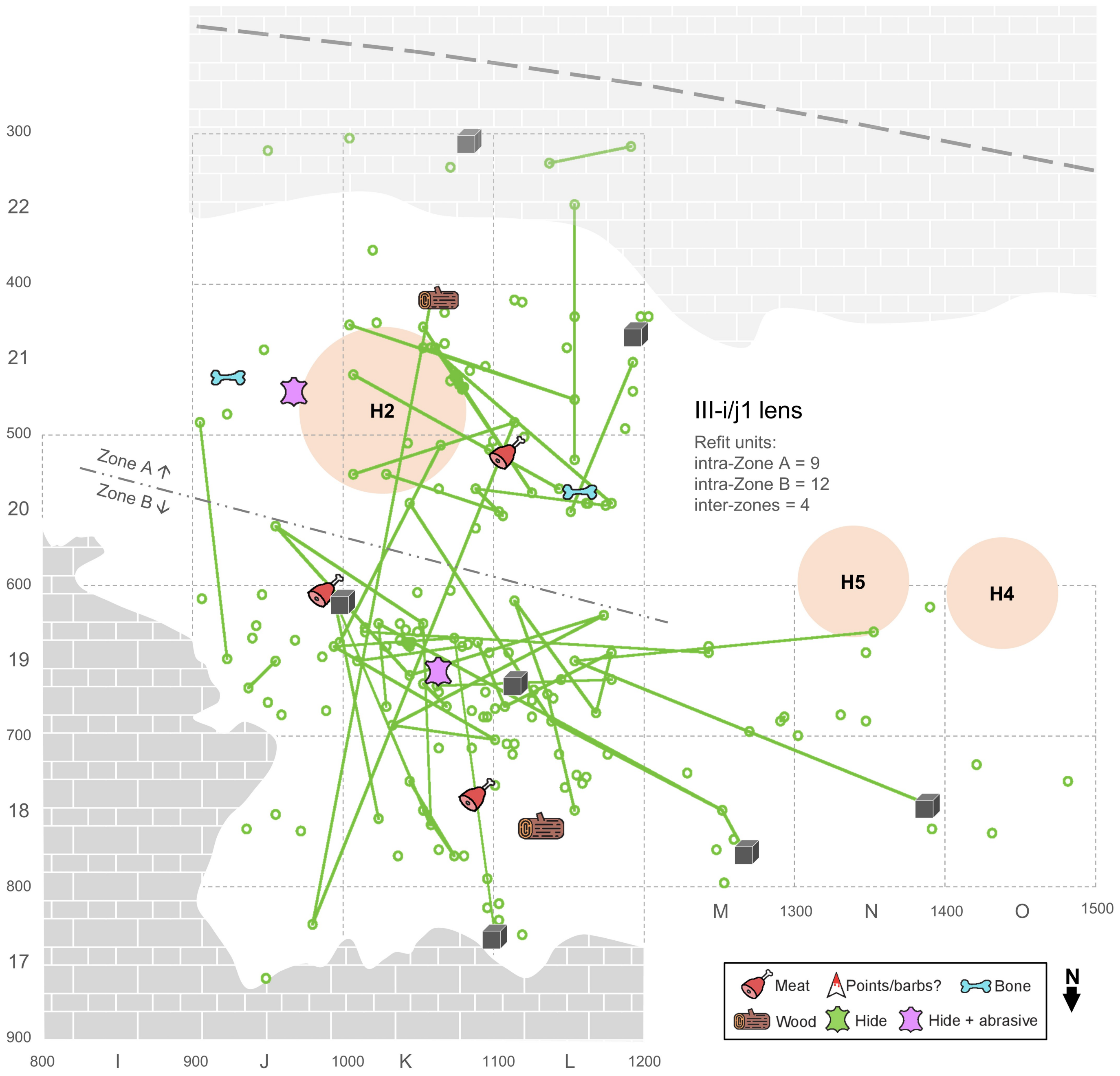

Fig. S29. III-i/j1 lens: spatial structure and activities. The grey cubes represent piece-plotted cores-on-block, the circles represent other piece-plotted lithics, and the lines represent refit links (for the purposes of this representation, the sieve items included in refit units were assigned the [x,y] coordinate of the center of the square meter or quarter-square meter of provenience). The finds from layer III-i/j excavated in 1991 were assigned to this lens, except in three cases found to refit with material from the 2011-12 excavation of the i/j2-3 lens. The positions of Hearths 2 (H2), 4 (H4) and 5 (H5) are schematically indicated. The symbols coded by material type or function denote the position of stone tools bearing the corresponding, diagnostic use-wear. The outline of the bedrock shelf bounding the infill to the North is given as observed at the elevation of the layer's surface. The outline of the back wall is given as observed at the top of the sedimentary infill, prior to excavation, and, approximately (long-dash line), at the layer's surface, as inferred from the concave configuration displayed in the J>I, L>M (Fig. S2), and N>M stratigraphic profiles.

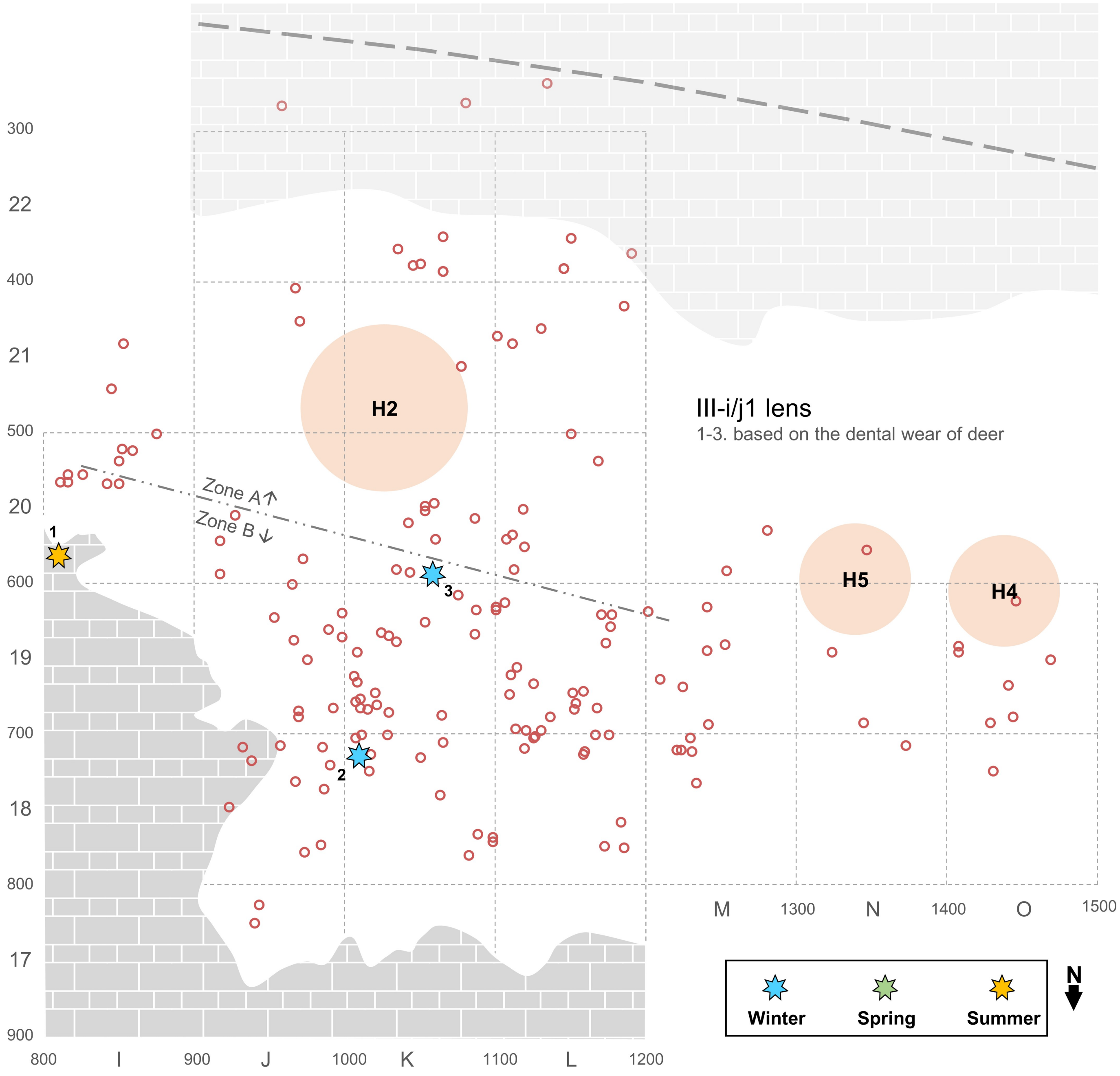

Fig. S30. III-i/j1 lens: spatial structure and seasonality. The points represent piece-plotted faunal remains (the sieve items with seasonality information were assigned the [x,y] coordinate of the center of the square meter or quarter-square meter of provenience). The positions of Hearths 2 (H2), 4 (H4) and 5 (H5) are schematically indicated. The outline of the bedrock shelf bounding the infill to the North is given as observed at the elevation of the layer's surface. The outline of the back wall is given as observed at the top of the sedimentary infill, prior to excavation, and, approximately (long-dash line), at the layer's surface, as inferred from the concave configuration displayed in the J>I, L>M (Fig. S2), and N>M stratigraphic profiles.

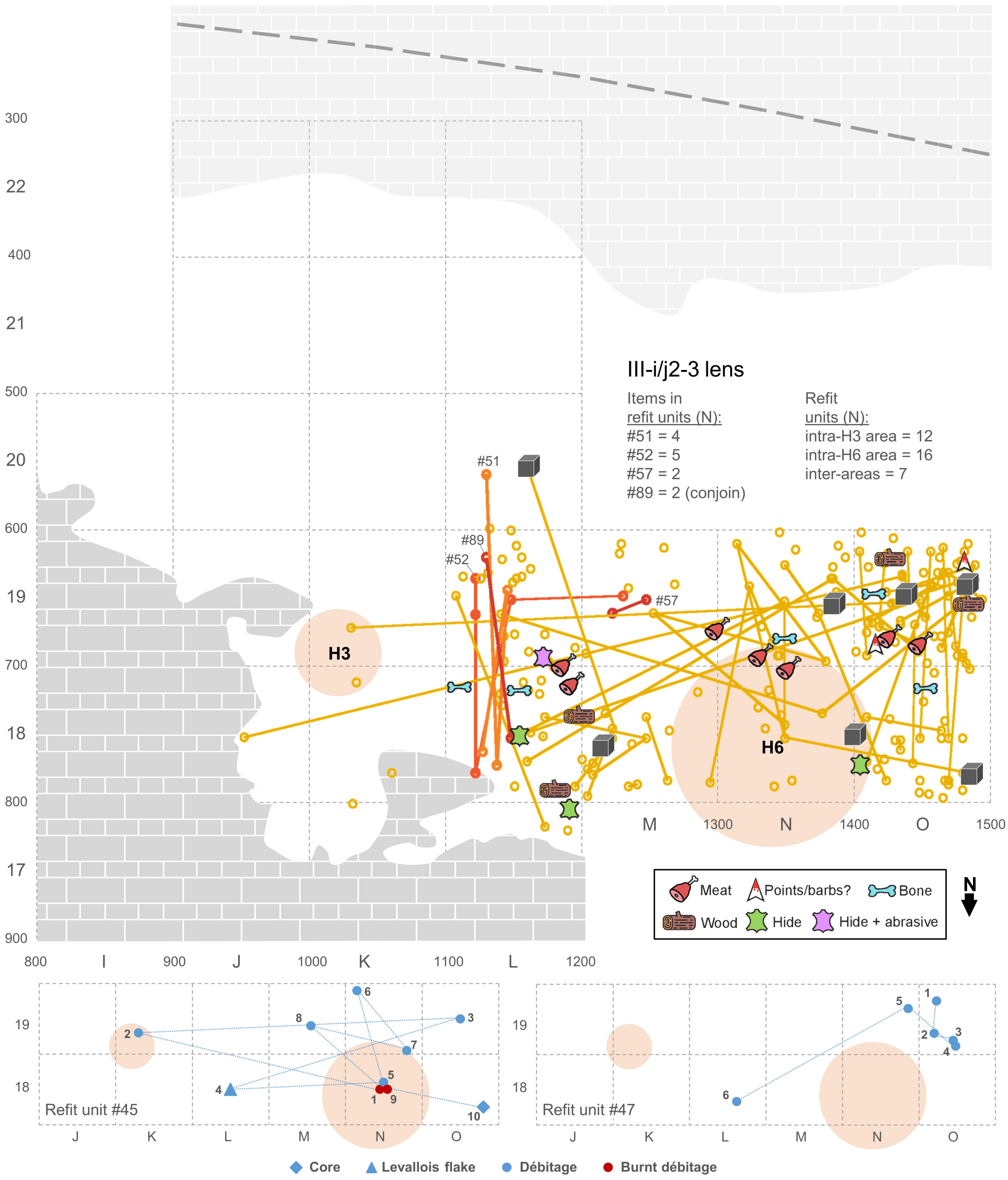

Fig. S31. III-i/j2-3 lens: spatial structure and activities. The grey cubes represent piece-plotted cores-on-block, the circles represent other piece-plotted lithics, and the lines represent refit links (for the purposes of this representation, the sieve items included in refit units were assigned the [x,y] coordinate of the center of the square meter or quarter-square meter of provenience). Two piece-plotted flakes from 1991 and five items from 2011-12 labelled “i/j1” in the field but coming from the interface between the two lenses (one core, one flake and three debris) refit with material from the i/j2-3 lens; they are included in the diagram. The positions of Hearths 3 (H3) and 6 (H6) are schematically indicated. The symbols coded by material type or function denote the position of stone tools bearing the corresponding, diagnostic use-wear. The outline of the bedrock shelf bounding the infill to the North is given as observed at the elevation of the layer’s surface. The outline of the back wall is given as observed at the top of the sedimentary infill, prior to excavation, and, approximately (long-dash line), at the layer’s surface, as inferred from the concave configuration displayed in the J>I, L>M (Fig. S2), and N>M stratigraphic profiles. The plots below represent one-way (#47) and two-way (#45) refit linkages between the two clusters, numbered according to the order of their extraction.

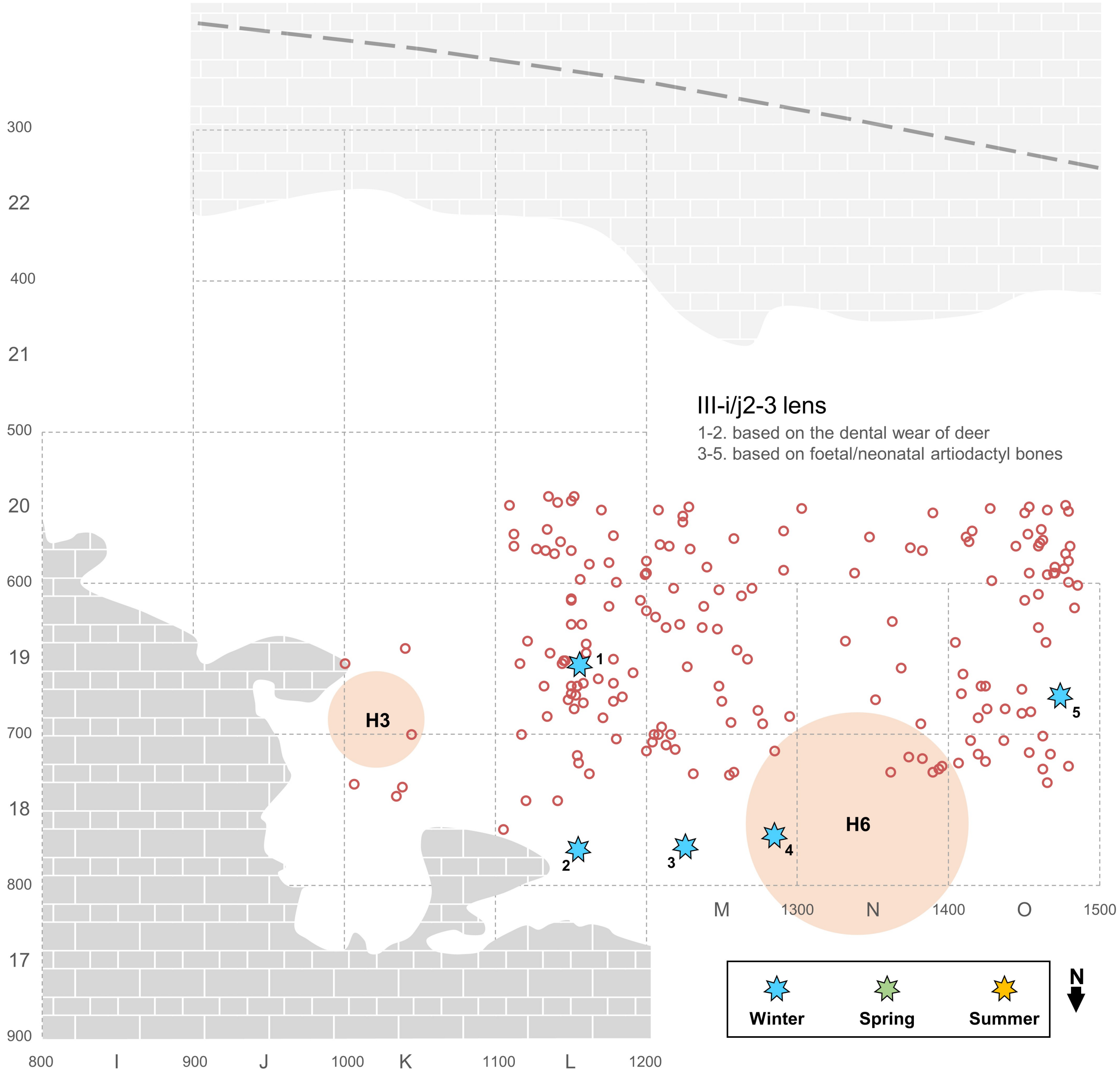

Fig. S32. III-i/j2-3 lens: spatial structure and seasonality. The points represent piece-plotted faunal remains (the sieve items with seasonality information were assigned the [x,y] coordinate of the center of the square meter or quarter-square meter of provenience). The positions of Hearths 3 (H3) and 6 (H6) are schematically indicated. The outline of the bedrock shelf bounding the infill to the North is given as observed at the elevation of the layer's surface. The outline of the back wall is given as observed at the top of the sedimentary infill, prior to excavation, and, approximately (long-dash line), at the layer's surface, as inferred from the concave configuration displayed in the J>I, L>M (Fig. S2), and N>M stratigraphic profiles.

Supplementary Tables

Table S1. Lithic inventory per techno-economic category and stratigraphic unit

	III-b/d		III-i/j1		III-i/j2-3		Total	
	N	%	N	%	N	%	N	%
Core	22	1.64	9	2.20	9	1.04	40	1.53
Débitage	702	52.23	232	56.59	431	50.00	1365	52.18
Tool	36	2.68	21	5.12	35	4.06	92	3.52
Tool-core	–	–	–	–	4	0.46	4	0.15
Resharpening	26	1.93	5	1.22	22	2.55	53	2.03
Debris	516	38.39	109	26.59	350	40.60	975	37.27
Tested cobble	13	0.97	10	2.44	–	–	23	0.88
Hammerstone	7	0.52	5	1.22	3	0.35	15	0.57
Cobble/Cobble fragment	22	1.64	19	4.63	8	0.93	49	1.87
Total	1344	100.00	410	100.00	862	100.00	2616	100.00

Table S2. Lithic raw materials per stratigraphic unit

		III-b/d	%	III-i/j1	%	III-i/j2-3	%	All	%
Chert	N	491	37%	280	68%	639	74%	1410	54%
	Weight (g)	1579.0	10%	1011.5	11%	1400.8	26%	3991.0	13%
Limestone	N	840	63%	123	30%	220	26%	1183	45%
	Weight (g)	12815.6	83%	6400.3	66%	3891.9	73%	23107.0	76%
Sandstone	N	9	1%	6	1%	3	0%	18	1%
	Weight (g)	369.2	2%	2212.9	23%	57.6	1%	2639.0	9%
Other	N	4	0%	1	0%		0%	5	0%
	Weight (g)	594.3	4%	0.1	0%		0%	594.0	2%
Total	N	1344	100%	410	100%	862	100%	2616	100%
	Weight (g)	15358.0	100%	9624.8	100%	5350.3	100%	30331.0	100%

Table S3. Layer III-b/d: tools and tool blanks

	cortical flake	>50% cortical flake	<50% cortical flake	naturally backed flake	débordant flake	Kombewa flake	Levallois flake	regular flake	small flake	flake fragment	Total	%
sidescraper	1	–	3	4	1	2	2	1	–	1	15	44
double sidescraper	–	–	–	–	–	–	2	1 (a)	–	–	3	9
inverse sidescraper	–	–	1	–	–	–	1	–	–	–	2	6
transverse scraper	–	–	1	–	–	–	–	–	–	–	1	1
sidescraper and notch	–	–	–	–	1	–	–	–	–	–	1	3
denticulate/notch	1	1	–	–	–	–	–	–	–	–	2	6
retouched flake	–	–	–	1	–	1	2	–	1	1	6	18
sidescraper fragment	–	–	–	–	–	–	–	–	–	4	4	12
Total	2	2	4	5	2	3	7	2	1	6	34	100
%	6	6	12	15	6	9	21	6	3	18		

(a) alternate

Table S4. Layer III-i/j, III-i/j1 lens: tools and tool blanks

	cortical flake	>50% cortical flake	<50% cortical flake	débordant flake	Levallois flake	pseudo-Levallois point	ordinary flake	flake fragment	Total	%
sidescraper	1	3	–	–	2	–	2	4	12	63
transverse scraper	–	–	–	–	2	–	–	1	3	16
double sidescraper	–	–	–	1 (a)	–	–	–	–	1	5
retouched flake	–	–	1	–	1	1	–	–	3	16
Total	1	3	1	1	5	1	2	5	19	100
%	5	16	5	5	26	5	11	26		

(a) convergent

Table S5. Layer III-i/j, III-i/j2-3 lens: tools and tool blanks

	cortical flake	>50% cortical flake	<50% cortical flake	naturally backed flake	Kombewa flake	Levallois flake	ordinary flake	flake	flake fragment	debris	Total	%
sidescraper	2	–	5 (a)	–	1	3	1	–	2 (b)	–	14	35
sidescraper and notch	–	–	1	–	–	–	–	–	–	–	1	3
double sidescraper	–	–	–	–	–	2	–	–	3	–	5	13
convergent sidescraper	–	–	1	1	–	3	1	–	–	–	6	15
Mousterian point	–	–	–	–	–	2	–	–	–	–	2	5
oblique scraper	–	–	–	–	–	1	–	–	–	–	1	3
transverse scraper	–	1	–	–	–	2	–	1	–	–	4	10
retouched flake	–	1	–	–	–	1	–	–	–	–	2	5
sidescraper fragment	–	–	–	1	–	–	–	–	3	1	5	13
Total	2	2	7	2	1	14	2	1	8	1	40	100
%	5	5	18	5	3	35	5	3	20	3		
basal thinning / core-on-tool (c)	–	–	3	2	–	–	–	1	1	–	7	

(a) two with scalariform retouch

(b) one with scalariform retouch

(c) on ventral or dorsal surface

Table S6. Types of retouch flakes

		III-b/d	III-i/j1	III-i/j2-3	Total	%
Preparation of the edge	type 0	3	–	–	3	5.6
	type 1	11	–	7	18	33.3
Resharpening	type 2	2	–	4	6	11.1
	type 3	8	5	12	25	46.3
	type 5	–	–	1	1	1.9
Undetermined		1	–	–	1	1.9
	Total	25	5	24	54	100.0
	%	46.3	9.3	44.4	100.0	

Table S7. Use wear: materials used on, per stratigraphic unit

	N	%
III-b/d	26	47.3
meat	2	3.6
bone	9	16.4
wood	12	21.8
wood (hafting wear)	1	1.8
hide (with abrasive)	2	3.6
III-i/j1	9	16.4
meat	3	5.5
bone	2	3.6
wood	2	3.6
hide (with abrasive)	2	3.6
III-i/j2-3	20	36.4
meat	7	12.7
bone	5	9.1
wood	4	7.3
hide	3	5.5
hide (with abrasive)	1	1.8
Total	55	100.0

Table S8. χ^2 results: p-values for use-wear differences between assemblages

	inter-units (b/d vs i/j1 vs i/j2-3)	inter i/j lenses (i/j1 vs i/j2-3)	inter-layers (b/d vs i/j)
Meat vs Bone vs Wood vs Hide (a)	0.153	0.997	0.025
(Meat + Bone) vs Wood vs Hide (a) (b)	0.280	0.975	0.081
Meat vs Bone	0.212	0.949	0.034

(a) N = 54 (one piece with on-wood hafting wear excluded)

(b) Meat + Bone = Carcass processing

Table S9. Cutting edge angles per stone tool type and raw material worked: individual measurements (in degrees), means, and standard deviations

sidescrapers					
		hide			
bone	wood	abrasive	other	all	meat
44	44	58	40	–	22
45	50	63	58	–	–
33	35	49	44	–	–
42	46	62	–	–	–
44	44	61	–	–	–
23	32	–	–	–	–
25	40	–	–	–	–
54	34	–	–	–	–
47	35	–	–	–	–
41	50	–	–	–	–
57	38	–	–	–	–
–	35	–	–	–	–
–	58	–	–	–	–
–	38	–	–	–	–
41.4	41.4	58.6	47.3	54.4	–
10.2	7.3	5.1	7.7	8.3	–

unretouched flakes			
bone	wood	hide	meat
25	50	–	21
35	36	–	22
23	23	–	22
25	32	–	22
–	32	–	27
–	–	–	33
–	–	–	45
–	–	–	29
–	–	–	38
–	–	–	27
–	–	–	38
–	–	–	–
–	–	–	–
–	–	–	–
27.0	34.6	–	29.4
4.7	8.8	–	7.7

Table S10. Skeletal representation of deer by layer and general anatomic category (after (10))

	Axial skeleton (a)		Greasy/meat-rich parts (b)		Medium members (c)		Lower members (d)		Other/ Undetermined (e)		Total	
	N	%	N	%	N	%	N	%	N	%	N	%
III-b/d	28	32.6	4	5.7	4	5.7	32	45.7	2	2.9	70	100.0
III-i/j	30	42.9	2	2.3	5	5.8	49	57.0	–	–	86	100.0
Total	58	37.2	6	3.8	9	5.8	81	51.9	2	1.3	156	100.0

(a) cranium, mandible, isolated teeth, vertebra, rib, sternum, and coxae

(b) scapula, humerus, femur

(c) radius, ulna, and tibia

(d) carpal/tarsal, calcaneus, metapodial, metacarpal, metatarsal, phalanx and *sesamoideum*

(e) flat bone, long bone, spongy bone and undetermined

Table S11. Skeletal representation of deer by layer and long bone category (after (10))

	Scapula, Femur, Humerus		Radius, Ulna, Tibia		Metacarpal, Metatarsal		Total	
	NR	%	NR	%	NR	%	NR	%
III-b/d	4	11.1	4	11.1	28	77.8	36	100.0
III-i/j	2	4.9	5	12.2	34	82.9	41	100.0
Total	6	7.8	9	11.7	62	80.5	77	100.0

Supplementary References

1. J. Zilhão, V. Villaverde, The Middle Paleolithic of Murcia. *Treb. Arqueol.* **14**, 229–248 (2008).

2. C. Martínez-Sánchez, El yacimiento musteriense de Cueva Antón (Mula, Murcia). *Mem. Arqueol Reg. N Murcia* **6**, 18–47 (1997).

3. J. Zilhão *et al.*, Symbolic use of marine shells and mineral pigments by Iberian Neandertals. *Proc. Natl. Acad. Sci.* **107**, 1023–1028 (2010).

4. J. Zilhão *et al.*, Cueva Antón: A multi-proxy MIS 3 to MIS 5a paleoenvironmental record for SE Iberia. *Quat. Sci. Rev.* **146**, 251–273 (2016).

5. J. Zilhão *et al.*, Precise dating of the Middle-to-Upper Paleolithic transition in Murcia (Spain) supports late Neandertal persistence in Iberia. *Heliyon* **3**, e00435 (2017).

6. D. E. Angelucci *et al.*, Formation processes at a high resolution Middle Paleolithic site: Cueva Antón (Murcia, Spain). *Quat Int.* **315**, 24-41 (2013).

7. D. E. Angelucci *et al.*, A tale of two gorges: Late Quaternary site formation and surface dynamics in the Mula basin (Murcia, Spain). *Quat Int.* **485**, 4–22 (2018).

8. S. O. Rasmussen *et al.*, A stratigraphic framework for abrupt climatic changes during the Last Glacial period based on three synchronized Greenland ice-core records: refining and extending the INTIMATE event stratigraphy. *Quat. Sci. Rev.* **106**, 14–28 (2014).

9. D. Anesin, Dinamiche formative di due siti di riferimento del Paleolitico nella Penisola Iberica sud-orientale. Analisi micromorfologica dei depositi di Cueva Antón e Cueva Negra (Spagna, Murcia) (2016).

10. M. Sanz, F. Rivals, D. García, J. Zilhão, Hunting strategy and seasonality in the last interglacial occupation of Cueva Antón (Murcia, Spain). *Archaeol. Anthropol. Sci.* **11**, 3577–3594 (2019).

11. J. Carranza, S. J. H. de Trucios, R. Medina, J. Valencia, J. Delgado, Space use by red deer in a Mediterranean ecosystem as determined by radio-tracking. *Appl. Anim. Behav. Sci.* **30**, 363–371 (1991).

12. M. N. Bugalho, J. A. Milne, The composition of the diet of red deer (Cervus elaphus) in a Mediterranean environment: a case of summer nutritional constraint? *For. Ecol. Manag.* **181**, 23–29 (2003).

13. G. Álvarez Jiménez, Ecología y gestión de las poblaciones de cérvidos, ciervo (“Cervus elaphus”), gamo (“Dama dama”) y corzo (“Capreolus capreolus”), en los Quintos de Mora (Montes de Toledo) (Universidad Complutense de Madrid, Servicio de Publicaciones, 2003).

14. C. H. Grohmann, G. Campanha, OpenStereo: open source, cross-platform software for structural geology analysis. *AGUFM* **2010**, IN31C-06 (2010).

15. C. Agostinelli, U. Lund, R package circular: Circular Statistics (version 0.4–93), CA: Department of Environmental Sciences, Informatics and Statistics, Ca’Foscari University, Venice, Italy. *UL Dep. Stat. Calif. Polytech. State Univ. San Luis Obispo Calif. USA* (2017).

16. R Core Team, R: A language and environment for statistical computing. R Foundation for Statistical Computing. Austria: Vienna (2018).

17. A. Lucena *et al.* "Avance al estudio de materias primas de los yacimientos paleolíticos de Rambla Perea (Mula, Murcia, España)" in Solutrean 2017. 3rd International Conference (Universidade do Algarve, Faro, 2017), pp. 43.

18. J.-M. Geneste, “Analyse lithique d’industries moustériennes du Périgord : une approche technologique du comportement des groupes humains au Paléolithique moyen” Université de Bordeaux I, Bordeaux, 1985).

19. J. Pelegrin, C. Karlin, P. Bodu, Chaînes opératoires: un outil pour le préhistorien. *Technol. Préhistorique* **25**, 55–62 (1988).

20. S. Kuhn, Mousterian Lithic Technology: An Ecological Perspective (Princeton University Press, 1995).

21. L. Binford, Organization and formation processes: looking at curated technologies. *J. Anthropol. Res.* **35**, 255–273 (1979).

22. L. Bourguignon, “Le Moustérien de type Quina : nouvelle définition d’une entité technique” (Université de Paris X - Nanterre, 1997).

23. 1. J. F. Gibaja, B. Gassin, "Use-Wear Analysis on Flint Tools. Beyond the Methodological Issues" in Use-Wear and Residue Analysis in Archaeology, J. M. Marreiros, J. F. Gibaja Bao, N. Ferreira Bicho, Eds. (Springer International Publishing, Cham, 2015), 10.1007/978-3-319-08257-8_4, pp. 41-58.

24. G. Pignat, H. Plisson, Le quartz, pour quel usage? L’outillage mésolithique de Vionnaz (CH) et l’apport de la tracéologie in Meso 97 : Actes de La Table Ronde Epipaléolithique et Mésolithique (Cahiers d´Archéologie Romande, 2000), pp. 65–78.

25. S. Korkmaz, D. Goksuluk, G. Zararsiz, MVN: An R Package for Assessing Multivariate Normality. *R J.* **6**, 151–162 (2014).

26. S. Le, J. Josse, F. Husson, FactoMineR: An R Package for Multivariate Analysis. *J. Stat. Softw.* **25**, 1–18 (2008).

27. W. Revelle, psych: Procedures for Personality and Psychological Research, Northwestern University, Evanston, Illinois, USA, https://CRAN.R-project.org/package=psych; Version = 1.8.12. (2018).

28. M. Hervé, RVAideMemoire: Testing and Plotting Procedures for Biostatistics (2019).

29. H. Dibble, M. Bernard, A comparison of basic edge angle measurement techniques. *Am. Antiq.* **45**, 857–865 (1980).

30. J.-B. Mallye, *et al.*, The Mousterian bone retouchers of Noisetier cave: experimentation and identification of marks. *J. Archaeol. Sci.* **39**, 1131–1142 (2012).

31. F. Collin, P. Jardon-Giner, "Travail de la peau avec des grattoirs emmanchés. Réflexions sur des bases expérimentales et ethnographiques" in Traces et fonction: les gestes retrouvés, Colloque internationale de Liège (Université de Liège, Liège, 1993), pp. 105–117.

32. B. Hayden, “Investigating status with hideworking use-wear: a preliminary assessment” in Traces et fonction: les gestes retrouvés, Colloque internationale de Liège (Université de Liège, Liège, 1993), pp. 119–130.

33. M. Calvo Trias, La memoria del útil. Análisis funcional de la industria lítica de la Cueva del Parco (Alós de Balaguer, la Noguera, Lleida). *Semin. Estud. Recer. Prehistòriques* (2004).

34. A. Laborda Martínez, Análisis funcional de los buriles de la cueva de Zatoya (Navarra). *Cuad. Arqueol. Univ. Navar.* **18**, 111–157 (2010).

35. C. Gutiérrez, Traceología. Pautas de análisis experimental. *Temas Arqueol.* **4** (1996).

36. J. J. Ibáñez-Estévez, J. E. González Urquijo, “La organización espacial de la producción y uso del utillaje de piedra en Berniollo” in *Análisis Funcional. Su Aplicación a Sociedades Prehistóricas.*, British Archaeological Reports, International Series., I. Clemente Conte, R. Risch, J. F. Gibaja, Eds. (Archaeopress, 2002), pp. 173–185.

37. C. Hincker, Matière et métier. Le travail des peaux et du cuir chez les Touaregs de l’Ouest (Mali) in *Actes Des XXII Rencontres Internationales D´Archéologie et D´Histoire D´Antibes*, F. Audoin-Rozuzeau, S. Beyries, Eds. (APDGA, 2002), pp. 99–112.

38. C. Chahine, Évolution des techniques de fabrication du cuir et problèmes des conservation in *Actes Des XXII Rencontres Internationales D´Archéologie et D´Histoire D´Antibes*, F. Audoin-Rozuzeau, S. Beyries, Eds. (APDGA, 2002), pp. 13–29.

39. M. De Bie, Benefiting from refitting in intra-site analysis: lessons from Rekem (Belgium). *BAR Int. Ser.* **1596**, 31 (2007).

40. L. Binford, *Nunamiut Ethnoarchaeology* (Academic press, 1978).
